# A new and convenient synthetic way to 2-substituted thieno[2,3-*b*]indoles

**DOI:** 10.3762/bjoc.11.112

**Published:** 2015-06-11

**Authors:** Roman A Irgashev, Arseny A Karmatsky, Gennady L Rusinov, Valery N Charushin

**Affiliations:** 1 Postovsky Institute of Organic Synthesis, Ural Division of the Russian Academy of Sciences, Ekaterinburg, 620990, Russia; 2 Ural Federal University named after the First President of Russia, B. N. Yeltsin, Ekaterinburg, 620002, Russia

**Keywords:** aldol-crotonic condensation, isatin, Lawesson’s reagent, methyl ketones, Paal–Knorr reaction, thieno[2,3-*b*]indole

## Abstract

A short and robust approach for the synthesis of 2-(hetero)aryl substituted thieno[2,3-*b*]indoles from easily available 1-alkylisatins and acetylated (hetero)arenes has been advanced. The two-step procedure includes the “aldol-crotonic” type of condensation of the starting materials, followed by treatment of the intermediate 3-(2-oxo-2-(hetero)arylethylidene)indolin-2-ones with Lawesson’s reagent. The latter process involves two sequential reactions, namely reduction of the C=C ethylidene double bond of the intermediate indolin-2-ones followed by the Paal–Knorr cyclization, thus affording tricyclic thieno[2,3-*b*]indoles.

## Introduction

**Figure 1:**
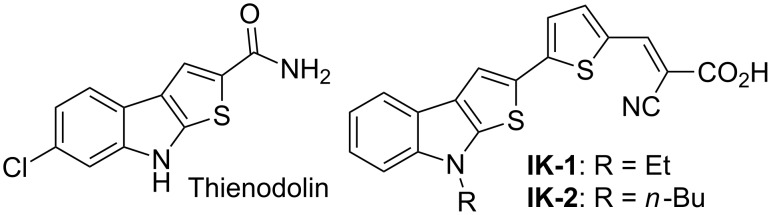
Natural and synthetic derivatives of thieno[2,3-*b*]indole.

8*H*-Thieno[2,3-*b*]indole is a fused heterocyclic system, which has attracted a considerable attention of researchers, mainly due to the fact that thieno[2,3-*b*]indole derivatives exhibit a wide range of biological properties, and can be regarded as promising compounds for agricultural or pharmacological applications. For example, the alkaloid thienodolin [1], isolated from the fermentation mixture of *Streptomyces albogriseolus* and characterized by Kanbe et al. [2–3], has shown to exhibit a plant-growth-regulation activity (Figure 1). Furthermore, it has been reported that some thienoindoles are therapeutically agents for treating diseases of the central nervous system [4], potential inhibitors of acetylcholine esterase and butyrylcholine esterase [5], compounds of this series exhibit as well anti-tuberculosis [6] and anti-inﬂammatory activities [7]. Another promising area for the use of thieno[2,3-*b*]indoles, as electron-rich heteroaromatics, is the design of photo- and electroactive compounds which can be applied in organic optoelectronic materials. Indeed, we have recently reported the synthesis of novel push–pull dyes **IK-1,2** based on the thieno[2,3-*b*]indole ring system, as a donating part of dye-sensitized solar cells [8] (Figure 1). It should be noted that thieno[2,3-*b*]pyrrole and thieno[3,2-*b*]pyrrole ring systems have been incorporated in the structures of various fused polycyclic compounds (heteroacenes), which have been used as efficient hole-transport materials for organic light emitting diodes (OLEDs) or field effect transistors (OFETs) [9–15].

Taking into account that the overwhelming majority of compounds for organic electronics are constructed from π-conjugated linear or angular fused aromatic and heteroaromatic units [16–19], the development of convenient synthetic ways to structures bearing several linked (het)aromatic units appears to be a very important issue. It means that synthetic methods should be based on a minimum number of steps (one-pot procedure as the perfect case), while the starting materials are supposed to be easily available.

**Scheme 1:**
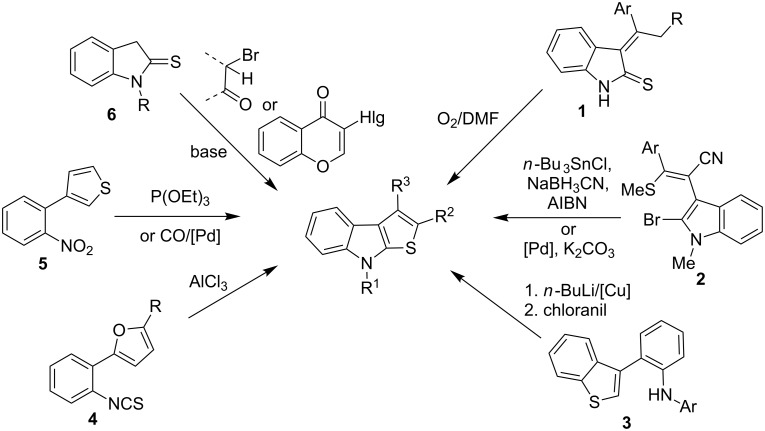
Synthetic routes to thieno[2,3-*b*]indoles.

A number of synthetic routes to thieno[2,3-*b*]indoles have been described in the literature, including oxidative cyclization of indolin-2-thiones **1** [20], radical or palladium catalyzed cyclization of 3-(2-bromoindol-3-yl)acrylonitriles **2** [21–22], intramolecular CH/NH-coupling in benzo[*b*]thiophenes **3** [23], AlCl_3_ catalyzed recyclization of 2-(2-isothiocyanatophenyl)furanes **4** [24], reductive cyclization of 3-(2-nirtophenyl)thiophenes **5** via nitrene intermediates [25–26], and condensation of 3-unsubstituted indolin-2-thione **6** with aliphatic α-bromoaldehydes, α-bromoketones [27] or 3-halochromones (Hlg = Cl, Br) [28] under basic conditions (Scheme 1). However, all synthetic methods mentioned above are based on using functionalized indoles, thiophenes, furans or chromone precursors, which require several steps to be prepared.

## Results and Discussion

**Scheme 2:**
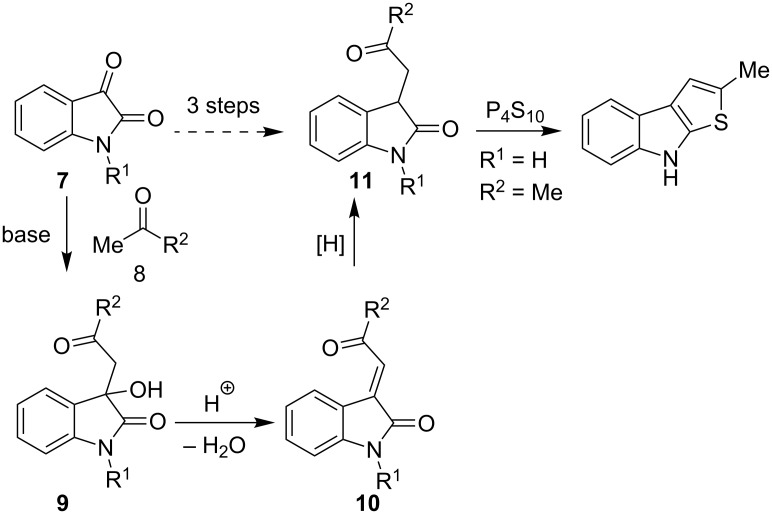
Synthesis and thionation of indodin-2-ones **11**.

In this paper we wish to report a convenient, short and robust approach to 8-alkyl-2-(het)arylthieno[2,3-*b*]indoles from 1-alkylisatins and the corresponding acetylated (hetero)arenes which are easily accessible reagents, including commercially available ones. It is well known that the reaction of isatins **7** with methyl ketones **8** leads to the formation of aldol-type adducts **9** under catalysis with mild bases, such as secondary or tertiary amines. These adducts can be dehydrated easily by acidic agents to form crotonic condensation products, namely 3-(2-oxo-2-(hetero)arylethylidene)indolin-2-ones **10**, which can undergo reduction of the C=C double bond in the presence of Na_2_S_2_O_4_ [29], H_2_/Pd(C) [30], or Me_3_P–H_2_O [31] (Scheme 2) into the corresponding indolin-2-ones **11**. Compounds **11** bearing the fragment of 4-oxobutyramides (1,4-dicarbonyl derivatives) can be cyclized into thieno[2,3-*b*]indoles by using the Paal–Knorr reaction with such thionation agents, as P_4_S_10_ or Lawesson’s reagent. This four-step route to thieno[2,3-*b*]indoles via the formation of indoline-2-ones **11** from isatins and methyl ketones has previously been realized [32–33]. In particular, preparation of 2-methyl-8*H*-thieno[2,3-*b*]indole from unsubstituted isatin and acetone in 15% yield has been reported [32] (Scheme 2). Although it seems to be a very harmonious strategy, it has hardly a significant preparative interest, since the target compounds are formed from isatins **7** and ketones **8** in four steps in low yields.

**Scheme 3:**
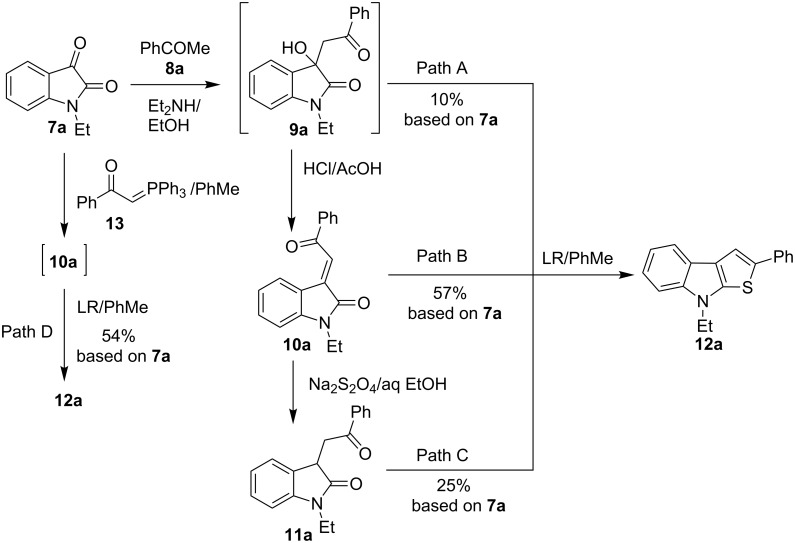
Synthetic paths to thieno[2,3-*b*]indole **12a**. LR = Lawesson's reagent

**Figure 2:**
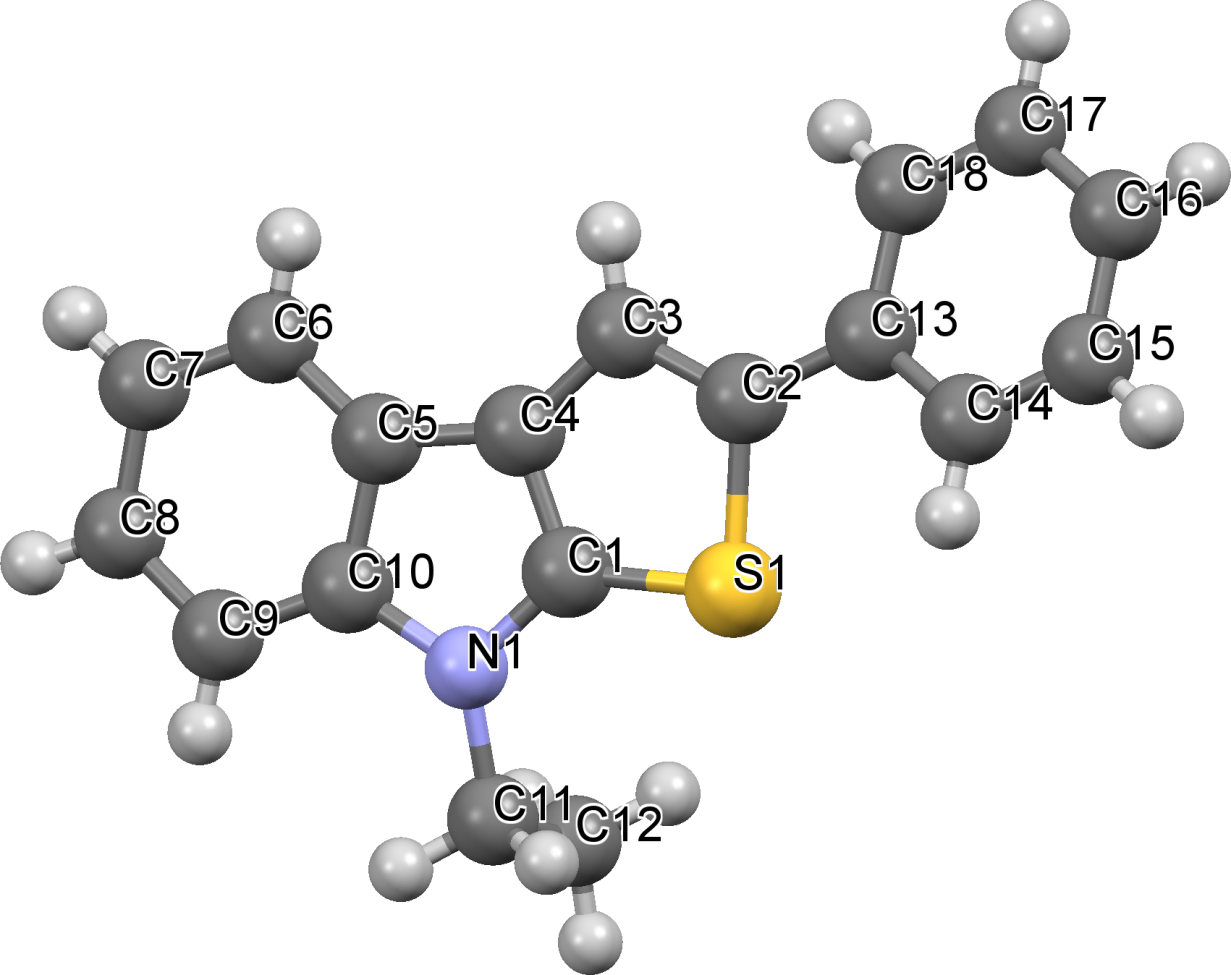
*Mercury* [34] representation of the X-ray crystal structure of **12a**. Thermal ellipsoids of 50% probability are presented.

The formation of model thieno[2,3-*b*]indole **12a** from 1-ethylisatin (**7a**) and acetophenone (**8a**) has been studied in details, as the first step of our research (Scheme 3). The conventional path [32] (Path C) leading to the aldol adduct **9a** (which was further used without purification in all our experiments), its dehydration into **10a**, reduction of the latter to indolin-2-ones **11a**, and finally cyclization of **11a** by action of Lawesson’s reagent (LR), resulting in the formation of the desired product **12a** in an overall yield of 25%. We have found that treatment of compound **10a** with Lawesson’s reagent in toluene solution under reflux for 1 h leads to **12a** as well; however the best overall yield of 57% has only been reached (Path B). The evidence for the structure of **12a** has been obtained unequivocally by X-ray crystallography analysis, thus supporting the data of ^1^H and ^13^C NMR spectroscopy (Figure 2).

The Lawesson’s reagent appears to act firstly as a source of hydrogen sulfide to reduce the C=C double bond in compound **10a**, and secondly, as the thiation agent to form thieno[2,3-*b*]indole **12a** by means of the Paal–Knorr reaction. Compound **9a** has also been treated with the Lawesson's reagent in toluene to give the title product **12a** via intermediacy of **10a** in a low 10% yield, based on the starting isatin **7a** (Path A). Also the one-pot synthesis (Path D) of compound **12a** has been realized through treatment of isatin **7a** with (phenacylidene)triphenylphosphorane **13** and subsequent cyclization of the intermediate **10a** according the Path B. The yield of the target product **12a** obtained by the one-port procedure proved to be close to that of **12a** deriving from the Path B. Path D requires the more expensive phosphorane derivative **13** which is formed by pre-functionalization of acetophenone (**8a**), and this approach can be regarded as an alternative synthetic route just in some specific cases. Thus, the two-step approach to convert isatins **7** into thieno[2,3-*b*]indoles **12** via intermediacy of **10** (Path B) has been selected as the most effective and convenient one.

**Scheme 4:**
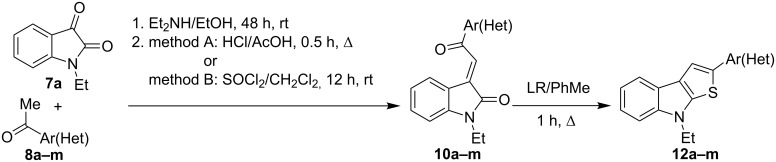
Two-step synthesis of 2-(hetero)aryl substituted thieno[2,3-*b*]indoles **12**.

Thereafter, a series of thieno[2,3-*b*]indoles **12a–m** bearing both electron-rich and electron-deficient (hetero)aromatic fragments at C-2 have been prepared in good to moderate yields via the two-step synthetic procedure (Path B, Scheme 3) from isatin **7a** and the corresponding acetylated (hetero)arenes **8a–m**. Dehydration of the initially obtained aldol-type adducts into 3-(2-oxo-2-(hetero)arylethylidene)indolin-2-ones **10** has been carried out in acetic acid solution with addition of hydrochloric acid (method A), or in CH_2_Cl_2_ solution with an excess of SOCl_2_ (method B), when compounds **10** failed to be obtained by method A (Table 1, Scheme 4). It should be noted that thieno[2,3-*b*]indole derivatives **12e,f** bearing 4-CN- or 2-NO_2_-phenyl substituents at C-2 have been prepared in high yields from the appropriate indolin-2-ones **10e,f** by treatment with Lawesson’s reagent under the current reaction conditions without displacement of CN- or NO_2_-groups (Table 1, entries 5 and 6).

**Table 1:** Yields of thieno[2,3-*b*]indoles (T[2,3-*b*]I) **12** and their precursors **10**.

Entry	Keton **8**	(Het)Ar	Method for **10**	Indolin-2-one **10**	Yield of **10** (%)	T[2,3-*b*]I **12**	Yield of **12** (%)

1	**8a**	Ph	A	**10a**	63	**12a**	90
2	**8b**	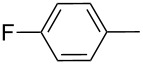	A	**10b**	65	**12b**	90
3	**8c**	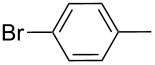	A	**10c**	69	**12c**	94
4	**8d**	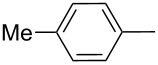	A	**10d**	57	**12d**	79
5	**8e**	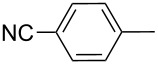	B	**10e**	75	**12e**	92
6	**8f**	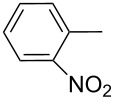	B	**10f**	81	**12f**	82
7	**8g**	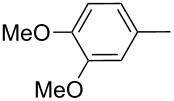	A	**10g**	74	**12g**	56
8	**8h**	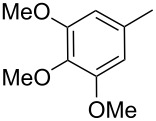	A	**10h**	91	**12h**	70
9	**8i**	C_6_F_5_	A	**10i**	33	**12i**	77
11	**8j**	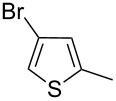	A	**10j**	76	**12j**	75
10	**8k**	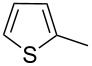	A	**10k**	76	**12k**	74
12	**8l**	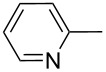	B	**10l**	66	**12l**	61
13	**8m**	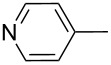	B	**10m**	67	**12m**	60

**Scheme 5:**
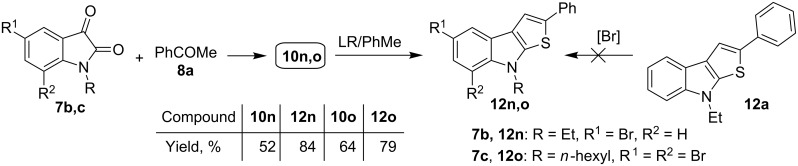
Synthesis of mono- and dibromo-substituted thieno[2,3-*b*]indoles **12n,o**.

Additionally, compounds **12n,o** bearing one or two bromine atoms at C-5 or C-5,7 of the thieno[2,3-*b*]indole scaffold have been prepared successfully from the corresponding isatins **7b,c** and acetophenone (**8a**) according to the synthetic procedure described above. Isatin **7c** containing an *n*-hexyl substituent at the nitrogen atom has been applied to ensure a good solubility of the target product **12o**, as well as of the intermediate **10o**. It should be noted that bromination of 2-phenyl-substituted thieno[2,3-*b*]indole **12a** led to a rather complicated mixture of compounds, and all attempts to reach bromination of compound **12a** with an excess of Br-agent (e.g., Br_2_/1,4-dioxane, NBS/CHCl_3_) proved to be unsuccessful (Scheme 5).

## Conclusion

In summary, we have developed a convenient and robust synthetic approach to thieno[2,3-*b*]indoles, bearing a wide range of aromatic and heteroaromatic substituents at C-2 with various electronic characteristics. Target thieno[2,3-*b*]indoles have been synthesized from 3-(2-oxo-2-(hetero)arylethylidene)indolin-2-one by treatment of the latter with Lawesson’s reagent through a tandem reduction of the C=C double bond and the Paal–Knorr cyclization, thus affording tricyclic thieno[2,3-*b*]indoles. In its turn, the required indolin-2-ones have been prepared by a simple “aldol-crotonic” type of condensation of *N*-alkylated isatins with acetophenones or their heterocyclic analogues. This two-step approach provides an easy access to compounds of the family of electron-rich thieno[2,3-*b*]indoles, which are regarded as promising building-blocks for the development of new photo- and electrosensitive molecules, e.g., novel push–pull dyes for dye-sensitized solar cells.

## Experimental

### General information

^1^H and ^13^C NMR spectra were obtained on Bruker DRX-400 and AVANCE-500 spectrometers with TMS as the internal standard. Elemental analysis was carried on an Eurovector EA 3000 automated analyzer. Mass spectrometry was performed using a Bruker maXis Impact HD spectrometer. Melting points were determined on Boetius combined heating stages and were not corrected. All solvents used were dried and distilled per standard procedures. IR spectra of samples (solid powders) were recorded on a Spectrum One Fourier transform IR spectrometer (Perkin Elmer) equipped with a diffuse reflectance attachment (DRA). X-ray diffraction analysis was performed on an automated X-ray diffractometer “Xcalibur E” on standard procedure.

### General procedure for the synthesis of 3-(2-oxo-2-(hetero)arylethylidene)indolin-2-one **10a**−**10o**

The solution of 1-alkylisatin **7** (3 mmol), corresponding methyl ketone **8** (3 mmol) and *N*,*N*-diethylamine (0.062 mL, 0.6 mmol) in EtOH (15 mL) was stirred at room temperature for 48 h. The resulting mixture was concentrated under reduced pressure to obtain crude adduct **9** that was dehydrated without any purification according to method A or method B.

**Method A (For the preparation of compounds 10a–d, 10g–k):** A drop of hydrochloric acid was added to the suspension of crude adduct **9** in acetic acid (3 mL). The mixture was stirred at 100 °C for 30 min. After cooling the precipitate was filtered, washed with methanol and dried to give indolin-2-one **10** as an orange to dark-red solid.

**1-Ethyl-3-(2-oxo-2-phenylethylidene)indolin-2-one (10a):** Orange powder; Yield 525 mg (63%); mp 122–123 °C; ^1^H NMR (500 MHz, DMSO-*d*_6_) δ 8.08 (dd, *J* = 8.2, 0.9 Hz, 2H), 8.00 (d, *J* = 7.6 Hz, 1H), 7.79 (s, 1H), 7.73 (t, *J* = 7.4 Hz, 1H), 7.61 (t, *J* = 7.8 Hz, 2H), 7.47–7.40 (m, 1H), 7.13 (d, *J* = 7.9 Hz, 1H), 7.02 (t, *J* = 7.6 Hz, 1H), 3.79 (q, *J* = 7.2 Hz, 2H), 1.20 (t, *J* = 7.2 Hz, 3H); ^13^C NMR (126 MHz, DMSO-*d*_6_) δ 191.3, 166.3, 144.8, 136.8, 135.2, 134.1, 132.8, 129.1, 128.6, 126.8, 126.4, 122.1, 119.4, 109.2, 34.3, 12.5; IR(DRA): 493, 546, 577, 600, 630, 674, 691, 750, 779, 802, 877, 894, 949, 1009, 1067, 1089, 1108, 1159, 1181, 1228, 1293, 1347, 1446, 1469, 1482, 1578, 1600, 1620, 1658, 1701, 1823, 1921, 2873, 2933, 2975, 3056, 3114 cm^−1^; anal. calcd for C_18_H_15_NO_2_: C, 77.96; H, 5.45; N, 5.05; found: C, 77.66; H, 5.34; N, 5.08.

**Method B (For the preparation of compounds 10e,f,l,–o):** SOCl_2_ (0.28 ml, 3.9 mmol) was added to the solution of crude adduct **9** in dry CH_2_Cl_2_ (10 ml). The mixture was stirred at room temperature for 2 h. The resulting mixture was concentrated under reduced pressure and the residue was recrystallized in EtOH affording indolin-2-one **10** as orange to dark-red needles.

**1-Ethyl-3-(2-(2-nitrophenyl)-2-oxoethylidene)indolin-2-one (10f):** Red crystals; Yield 780 mg (81%); mp 120–121 °C; ^1^H NMR (400 MHz, DMSO-*d*_6_) δ 8.41 (d, *J* = 7.6 Hz, 1H), 8.21 (dd, *J* = 8.1, 0.7 Hz, 1H), 7.97–7.82 (m, 3H), 7.52 (td, *J* = 7.8, 1.0 Hz, 1H), 7.33 (s, 1H), 7.16 (d, *J* = 7.9 Hz, 1H), 7.11 (td, *J* = 7.7, 0.8 Hz, 1H), 3.77 (q, *J* = 7.2 Hz, 2H), 1.18 (t, *J* = 7.2 Hz, 3H); ^13^C NMR (126 MHz, DMSO-*d*_6_) δ 191.3, 166.4, 146.2, 145.5, 136.4, 135.8, 134.7, 133.9, 132.2, 128.8, 127.5, 125.8, 124.6, 122.4, 119.3, 109.3, 34.3, 12.5; IR(DRA): 473, 490, 548, 567, 584, 642, 675, 690, 746, 778, 846, 897, 932, 1002, 1058, 1080, 1128, 1164, 1187, 1224, 1285, 1324, 1348, 1391, 1414, 1464, 1485, 1502, 1584, 1608, 1629, 1653, 1709, 1780, 2648, 2837, 2939, 3021, 3402 cm^−1^; anal. calcd for C_18_H_14_N_2_O_4_: C, 67.07; H, 4.38; N, 8.69; found: C, 67.03; H, 4.12; N, 8.62.

### Procedure for the one-pot synthesis of thieno[2,3-*b*]indole **12a**

The solution of *N*-ethylisatin **7a** (175 mg, 1 mmol) and (phenacylidene)triphenylphosphorane **13** (380 mg, 1 mmol) in dry toluene (5 mL) was stirred at room temperature for 48 h. Afterwards Lawesson’s reagent (405 mg, 1 mmol) was added and the mixture was refluxed for 1 h. The resulting solution was concentrated under reduced pressure and the residue was dissolved in CH_2_Cl_2_ and filtered through a silicagel pad. After evaporation of CH_2_Cl_2_ the crude product was purified by recrystallization from EtOH giving thieno[2,3-*b*]indoles **12a** as white needles in 54% yield (150 mg).

### General procedure for the synthesis of thieno[2,3-*b*]indoles **12a**–**12o**

The mixture of indolin-2-one **10** (2 mmol) and the Lawesson reagent (0.81 g, 2 mmol) in dry toluene (10 mL) was refluxed for 1 h. Upon refluxing the color of the solution turns from dark-red to yellowish. The resulting solution was concentrated under reduced pressure and the residue was dissolved in CH_2_Cl_2_ and filtered through a silicagel pad. After evaporation of CH_2_Cl_2_ the crude product was purified by recrystallization in EtOH affording thieno[2,3-*b*]indoles **12** as white to yellow needles.

**8-Ethyl-2-phenyl-8*H*-thieno[2,3-*b*]indole (12a):** Pale yellow needles; Yield 500 mg (90%); mp 82–83 °C; ^1^H NMR (500 MHz, CDCl_3_) δ 7.81 (d, *J* = 7.8 Hz, 1H), 7.67–7.57 (m, 3H), 7.42–7.33 (m, 3H), 7.30–7.26 (m, 1H), 7.25–7.21 (m, 1H), 7.21–7.16 (m, 1H), 4.28 (q, *J* = 7.3 Hz, 2H), 1.52 (t, *J* = 7.3 Hz, 3H); ^1^H NMR (500 MHz, DMSO-*d*_6_) δ 7.92 (s, 1H), 7.82 (d, *J* = 7.7 Hz, 1H), 7.66 (d, *J* = 7.3 Hz, 2H), 7.58 (d, *J* = 8.2 Hz, 1H), 7.44–7.36 (m, 2H), 7.28–7.21 (m, 2H), 7.19–7.11 (m, 1H), 4.34 (q, *J* = 7.2 Hz, 2H), 1.40 (t, *J* = 7.2 Hz, 3H); ^13^C NMR (126 MHz, DMSO-*d*_6_) δ 141.6, 140.8, 135.0, 134.8, 129.1, 126.7, 124.5, 123.4, 122.0, 121.5, 119.4, 119.1, 114.8, 109.9, 40.3, 13.6; IR(DRA): 474, 550, 567, 688, 746, 779, 835, 850, 905, 935, 999, 1015, 1049, 1078, 1100, 1131, 1162, 1191, 1207, 1252, 1330, 1350, 1377, 1393, 1409, 1439, 1477, 1496, 1524, 1595, 1671, 1734, 1776, 1867, 1894, 1942, 2888, 2928, 2972, 3029, 3053, 3748 cm^−1^; MS (+APCI): Calcd. for C_18_H_15_NS *m*/*z* 278.0998 [M + H], found *m*/*z* 278.1000 [M + H]; Crystal data for **12a**: Colorless crystals 0.24 × 0.19 × 0.13 mm, θ < 25.6080°, 11563 reflections were collected, 7249 independent reflections (*R*_int_ 0.0245), completeness 100%. Crystal is monoclinic, space group Cc, *a* = 19.0695(13) Å, *b* = 30.6399(16) Å, *c* = 7.8360(4) Å, α = 90.00°, β = 106.777(6)°, γ = 90.00°, μ = 0.210 mm^−1^. The SHELXTL program [35] was used for solution and structure refinement. Refinement and the final *R* indices: *R*_1_ = 0.0432 [I>2σ(I)], wR_2_ = 0.1050 [I>2σ(I)], *R*_1_ = 0.0772 (all data), w*R*_2_ = 0.1355 (all data), *S* = 1.004. Deposition number CCDC 1054153 contains the supplementary crystallographic data for this structure. These data can be obtained free of charge from the Cambridge Crystallographic Data Centre via http://www.ccdc.cam.ac.uk/data_request/cif.

### 8-Ethyl-2-(2-nitrophenyl)-8*H*-thieno[2,3-*b*]indole (**12f**)

Yellow powder; Yield 530 mg (82%); mp 103–104 °C; ^1^H NMR (400 MHz, DMSO-*d*_6_) δ 7.94 (d, *J* = 7.8 Hz, 1H), 7.87 (d, *J* = 7.8 Hz, 1H), 7.77–7.71 (m, 2H), 7.67–7.53 (m, 3H), 7.33–7.27 (m, 1H), 7.22–7.13 (m, 1H), 4.37 (q, *J* = 7.2 Hz, 2H), 1.41 (t, *J* = 7.2 Hz, 3H); ^13^C NMR (126 MHz, DMSO-*d*_6_) δ 148.9, 143.0, 140.8, 132.5, 131.7, 128.6, 127.6, 127.0, 123.9, 123.3, 122.4, 121.3, 119.6, 119.4, 118.7, 110.0, 40.4, 13.5; IR(DRA): 482, 549, 567, 584, 646, 662, 685, 700, 738, 746, 770, 834, 845, 861, 919, 941, 953, 986, 1015, 1052, 1084, 1134, 1162, 1204, 1251, 1267, 1283, 1296, 1331, 1375, 1399, 1411, 1439, 1464, 1484, 1532, 1570, 1599, 1652, 1751, 1788, 1836, 1871, 1909, 1936, 1969, 2332, 2736, 2889, 2936, 2974, 2989, 3075, 3058 cm^−1^; Anal. calcd for C_18_H_14_N_2_O_2_S: C, 67.06; H, 4.38; N, 8.69; found: C, 66.98; H, 4.35; N, 8.72.

## Supporting Information

File 1 Analytical data and copies of the ^1^H and ^13^C NMR spectra of the new compounds.
