# Supplementary material for: A new and convenient synthetic way to 2-substituted thieno[2,3-b]indoles
Source: Beilstein J Org Chem. 2015 Jun 11;11:1000–7. doi: 10.3762/bjoc.11.112 (PMC4505097; doi:10.3762/bjoc.11.112)
Supplement: File 1 — Analytical data and copies of the 1H and 13C NMR spectra of the new compounds. [file Beilstein_J_Org_Chem-11-1000-s001.pdf]

Supporting Information

for

A new and convenient synthetic way to 2-substituted thieno[2,3-*b*]indoles

Roman A. Irgashev*[1,2], Arseny A. Karmatsky[1,2], Gennady L. Rusinov[1,2], Valery N. Charushin[1,2]

Address: [1]Postovsky Institute of Organic Synthesis, Ural Division of the Russian Academy of Sciences, Ekaterinburg, 620990, Russia and [2]Ural Federal University named after the First President of Russia, B. N. Yeltsin, Ekaterinburg, 620002, Russia

Email: Roman A. Irgashev - irgashev@ios.uran.ru

*Corresponding author

Analytical data and copies of the ^{1}H and ^{13}C NMR spectra of the new compounds

Table of contents

1. Analytical Data S2–S14

2. ^{1}H, ^{13}C NMR Spectra of indolin-2-ones **10** S15–S42

3. ^{1}H, ^{13}C NMR Spectra of thieno[2,3-b]indoles **12** S43–S71

3-(2-Oxo-2-(hetero)arylethylidene)indolin-2-ones 10

Compounds synthesized by method A:

1-Ethyl-3-(2-(4-fluorophenyl)-2-oxoethylidene)indolin-2-one (10b).

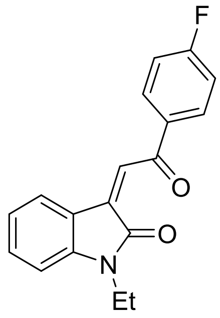

Red crystals; Yield 575 mg (65%); Mp 108 – 109 °C.

^{1}H NMR (500 MHz, DMSO-d_6) δ 8.19 – 8.11 (m, 2H), 7.99 (d, J = 7.6 Hz, 1H), 7.76 (s, 1H), 7.45 – 7.37 (m, 3H), 7.11 (d, J = 7.8 Hz, 1H), 7.04 – 6.97 (m, 1H), 3.77 (q, J = 7.2 Hz, 2H), 1.18 (t, J = 7.2 Hz, 3H); ^{13}C NMR (126 MHz, DMSO-d_6) δ 189.9, 166.3, 165.5 (d, J_{CF} = 253.5 Hz), 144.8, 135.3, 133.6 (d, J_{CF} = 2.7 Hz), 132.8, 131.8 (d, J_{CF} = 9.8 Hz), 126.5, 126.4, 122.2, 119.3, 116.2 (d, J_{CF} = 22.1 Hz), 109.2, 34.3, 12.5.

IR(DRA): 479, 489, 549, 574, 590, 607, 671, 712, 750, 784, 816, 845, 863, 883, 891, 950, 989, 1006, 1029, 1066, 1085, 1110, 1129, 1159, 1170, 1221, 1287, 1307, 1354, 1392, 1410, 1465, 1480, 1505, 1599, 1619, 1656, 1706, 1779, 2977, 3075, 3110 cm^{-1}.

Anal. Calcd. for $C_{18}H_{14}FNO_2$: C, 73.21; H, 4.78; N, 4.74. Found: C, 73.19; H, 4.56; N, 4.76.

3-(2-(4-Bromophenyl)-2-oxoethylidene)-1-ethylindolin-2-one (10c).

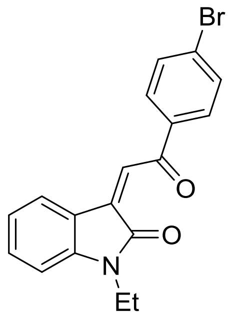

Red crystals; Yield 740 mg (69%); Mp 154 – 155 °C.

^{1}H NMR (500 MHz, DMSO-d_6) δ 8.07 – 7.98 (m, 3H), 7.82 (d, J = 8.6 Hz, 2H), 7.76 (s, 1H), 7.44 (td, J = 7.8, 1.0 Hz, 1H), 7.13 (d, J = 7.8 Hz, 1H), 7.03 (td, J = 7.7, 0.6 Hz, 1H), 3.78 (q, J = 7.2 Hz, 2H), 1.19 (t, J = 7.2 Hz, 3H); ^{13}C NMR (126 MHz, DMSO-d_6) δ 190.4, 166.3, 144.9, 135.9, 135.6, 133.0, 132.2, 130.6, 128.3, 126.5, 126.2, 122.2, 119.3, 109.3, 34.3, 12.5.

IR(DRA): 462, 490, 504, 548, 575, 603, 626, 645, 669, 697, 750, 784, 795, 826, 844, 866, 898, 936, 948, 1001, 1015, 1031, 1067, 1085, 1104, 1152, 1224, 1286, 1349, 1363, 1384, 1400, 1449, 1463, 1518, 1595, 1657, 1708, 1921, 2875, 2937, 2977, 3094, 3123, 3406 cm^{-1}.

Anal. Calcd. for $C_{18}H_{14}BrNO_2$: C, 60.69; H, 3.96; N, 3.93. Found: C, 60.47; H, 3.87; N, 3.89.

1-Ethyl-3-(2-oxo-2-(*p*-tolyl)ethylidene)indolin-2-one (10d).

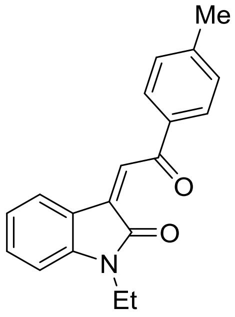

Red crystals; Yield 500 mg, (57%); Mp 99 – 100 °C.

^{1}H NMR (500 MHz, DMSO-d_6) δ 7.99 – 7.96 (m, 3H), 7.77 (s, 1H), 7.44 – 7.39 (m, 3H), 7.12 (d, J = 7.8 Hz, 1H), 7.01 (td, J = 7.7, 0.7 Hz, 1H), 3.78 (q, J = 7.2 Hz, 2H), 2.41 (s, 3H), 1.19 (t, J = 7.2 Hz, 3H); ^{13}C NMR (126 MHz, DMSO-d_6) δ 190.9, 166.3, 144.9, 144.7, 134.9, 134.4, 132.6, 129.7, 128.7, 127.2, 126.3, 122.1, 119.4, 109.2, 34.3, 21.3, 12.5.

IR(DRA): 467, 492, 549, 573, 606, 675, 711, 745, 780, 835, 852, 880, 889, 941, 964, 993, 1010, 1024, 1071, 1085, 1111, 1131, 1153, 1177, 1211, 1232, 1299, 1350, 1388, 1408, 1448, 1479, 1603, 1619, 1652, 1709, 1777, 1795, 1828, 1946, 2607, 2738, 2874, 2938, 2981, 3042, 3113, 3402 cm^{-1}.

Anal. Calcd. for $C_{19}H_{17}NO_2$: C, 78.33; H, 5.88; N, 4.81. Found: C, 78.00; H, 5.89; N, 4.77.

3-(2-(3,4-Dimethoxyphenyl)-2-oxoethylidene)-1-ethylindolin-2-one (10g).

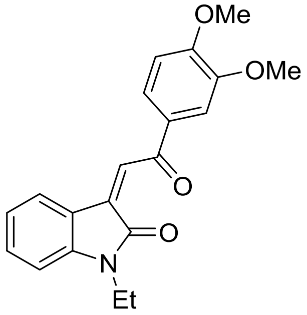

Orange powder; Yield 750 mg, (74%); Mp 143 – 144 °C.

^{1}H NMR (500 MHz, DMSO-d_6) δ 7.95 (d, J = 7.6 Hz, 1H), 7.78 (s, 1H), 7.74 (dd, J = 8.4, 2.0 Hz, 1H), 7.58 (d, J = 2.0 Hz, 1H), 7.41 (td, J = 7.9, 0.9 Hz, 1H), 7.15 – 7.09 (m, 2H), 7.03 – 6.98 (m, 1H), 3.88 (s, 3H), 3.87 (s, 3H), 3.78 (q, J = 7.2 Hz, 2H), 1.19 (t, J = 7.2 Hz, 3H); ^{13}C NMR (126 MHz, DMSO-d_6) δ 189.6, 166.4, 154.1, 149.1, 144.6, 134.5, 132.4, 129.8, 127.4, 126.3, 124.2, 122.1, 119.5, 111.1, 110.0, 109.1, 55.9, 55.5, 34.2, 12.5.

IR(DRA): 484, 551, 565, 583, 623, 673, 691, 751, 765, 784, 800, 844, 868, 920, 939, 959, 992, 1018, 1033, 1086, 1111, 1146, 1173, 1192, 1209, 1224, 1264, 1287, 1305, 1360, 1390, 1421,

1448, 1470, 1515, 1576, 1600, 1615, 1649, 1708, 1753, 1822, 2850, 2878, 2941, 2987, 3022, 3083 cm^{-1}.

Anal. Calcd. for $C_{20}H_{19}NO_4$: C, 71.20; H, 5.62; N, 4.15. Found: C, 71.20; H, 5.62; N, 4.08.

1-Ethyl-3-(2-oxo-2-(3,4,5-trimethoxyphenyl)ethylidene)indolin-2-one (10h).

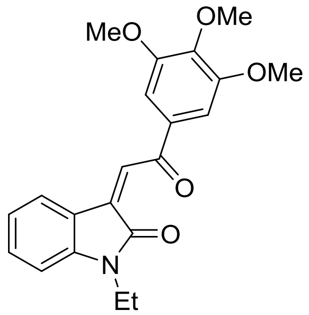

Red powder; Yield 1g (91%); Mp 122 – 123 °C.

^{1}H NMR (500 MHz, DMSO-d_6) δ 8.01 (d, J = 7.6 Hz, 1H), 7.81 (s, 1H), 7.45 – 7.39 (m, 1H), 7.36 (s, 2H), 7.11 (d, J = 7.9 Hz, 1H), 7.04 – 6.98 (m, 1H), 3.88 (s, 6H), 3.81 – 3.74 (m, 5H), 1.19 (t, J = 7.2 Hz, 3H); ^{13}C NMR (126 MHz, DMSO-d_6) δ 190.1, 166.4, 153.0, 144.7, 142.8, 135.3, 132.7, 132.2, 127.1, 126.4, 122.1, 119.4, 109.2, 106.2, 60.2, 56.1, 34.3, 12.5.

IR(DRA): 473, 490, 548, 567, 584, 642, 675, 690, 746, 778, 846, 897, 932, 1002, 1058, 1080, 1128, 1164, 1187, 1224, 1285, 1324, 1348, 1391, 1414, 1464, 1485, 1502, 1584, 1608, 1629, 1653, 1709, 1780, 2648, 2837, 2939, 3021, 3402 cm^{-1}.

Anal. Calcd. for $C_{21}H_{11}NO_5$: C, 68.65; H, 5.76; N, 3.81. Found: C, 68.41; H, 5.73; N, 3.80.

1-Ethyl-3-(2-oxo-2-(perfluorophenyl)ethylidene)indolin-2-one (10i).

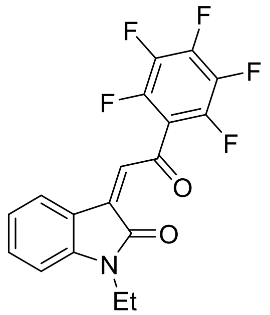

Dark-red powder; Yield 365 mg (33%); Mp 118 – 119 °C.

^{1}H NMR (500 MHz, DMSO-d_6) δ 8.46 (d, J = 7.5 Hz, 1H), 7.53 (td, J = 7.8, 1.0 Hz, 1H), 7.35 (t, J = 2.3 Hz, 1H), 7.15 (d, J = 7.9 Hz, 1H), 7.13 – 7.07 (m, 1H), 3.77 (q, J = 7.2 Hz, 2H), 1.19 (t, J = 7.2 Hz, 3H); ^{13}C NMR (126 MHz, DMSO-d_6) δ 182.9, 166.3, 145.9, 144.6 (dm*, J_{CF} = 244.8 Hz), 142.5 (dm*, J_{CF} = 242.9 Hz), 137.7, 137.2 (dm*, J_{CF} = 248.2 Hz), 134.6, 127.5, 126.0, 122.4, 119.1, 114.9 (t, J_{CF} = 16.4 Hz), 109.4, 34.4, 12.4.

*dm = doublet of multiplets.

IR(DRA): 480, 547, 569, 615, 641, 700, 723, 756, 767, 783, 801, 831, 862, 896, 920, 950, 988, 1034, 1049, 1087, 1106, 1161, 1227, 1290, 1314, 1349, 1363, 1388, 1409, 1468, 1498, 1519, 1596, 1618, 1650, 1674, 1712, 2882, 2939, 2979, 3037, 3127 cm^{-1}.

Anal. Calcd. for $C_{18}H_{10}F_5NO_2$: C, 58.86; H, 2.74; N, 3.81. Found: C, 58.85; H, 2.81; N, 3.90.

3-(2-(4-Bromothiophen-2-yl)-2-oxoethylidene)-1-ethylindolin-2-one (10j).

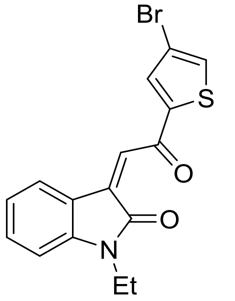

Red powder; Yield 825 mg (76%); Mp 160 – 161 °C.

^{1}H NMR (400 MHz, DMSO-d_6) δ 8.35 (d, J = 7.7 Hz, 1H), 8.27 (d, J = 1.0 Hz, 1H), 8.24 (d, J = 1.0 Hz, 1H), 7.70 (s, 1H), 7.52 – 7.42 (m, 1H), 7.12 (d, J = 7.9 Hz, 1H), 7.09 – 7.01 (m, 1H), 3.78 (q, J = 7.1 Hz, 2H), 1.19 (t, J = 7.1 Hz, 3H); ^{13}C NMR (126 MHz, DMSO-d_6) δ 181.9, 166.3, 145.4, 145.3, 136.8, 136.0, 134.1, 133.5, 127.6, 124.3, 122.2, 119.4, 110.6, 109.2, 34.3, 12.5.

IR(DRA): 472, 492, 549, 569, 595, 642, 707, 732, 753, 785, 815, 848, 875, 889, 948, 976, 1031, 1087, 1107, 1156, 1195, 1227, 1291, 1307, 1356, 1401, 1466, 1482, 1509, 1598, 1619, 1650, 1706, 1925, 2873, 2932, 2976, 3096 cm^{-1}.

Anal. Calcd. for $C_{16}H_{12}BrNO_2S$: C, 53.05; H, 3.34; N, 3.87. Found: C, 52.89; H, 3.29; N, 3.82.

1-Ethyl-3-(2-oxo-2-(thiophen-2-yl)ethylidene)indolin-2-one (10k).

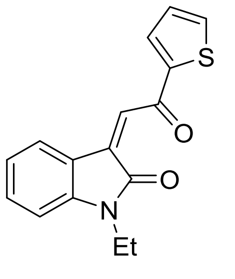

Red crystals; Yield 645 mg (76%); Mp 147 – 148 °C.

Indolin-2-one **10k** was previously described in the literature and its analytical data are identical to the reported data [1].

Compounds synthesized by method B:

3-(2-(4-Cyanophenyl)-2-oxoethylidene)-1-ethylindolin-2-one (10e).

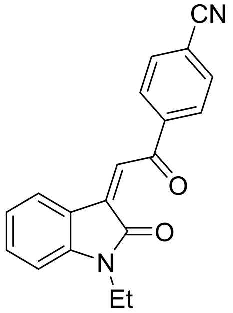

Dark-red crystals; Yield 680 mg (75%); Mp 176 – 177 °C.

^{1}H NMR (500 MHz, DMSO-*d*$_6$) δ 8.21 (d, *J* = 8.5 Hz, 2H), 8.12 (d, *J* = 7.5 Hz, 1H), 8.06 (d, *J* = 8.5 Hz, 2H), 7.77 (s, 1H), 7.45 (td, *J* = 7.8, 1.1 Hz, 1H), 7.13 (d, *J* = 7.9 Hz, 1H), 7.03 (td, *J* = 7.7, 0.8 Hz, 1H), 3.78 (q, *J* = 7.2 Hz, 2H), 1.18 (t, *J* = 7.2 Hz, 3H); ^{13}C NMR (126 MHz, DMSO-*d*$_6$) δ 190.3, 166.3, 145.2, 140.2, 136.4, 133.4, 133.1, 129.2, 126.8, 125.5, 122.3, 119.3, 118.1, 115.7, 109.3, 34.3, 12.5.

IR(DRA): 487,553, 565, 604, 643, 674, 718, 755, 783, 847, 887, 941, 991, 1022, 1070, 1087, 1111, 1134, 1159, 1177, 1226, 1291, 1358, 1390, 1406, 1467, 1605, 1619, 1656, 1705, 1929, 2229, 2877, 2938, 2978, 3097, 3116, 3402 cm^{-1}.

Anal. Calcd. for $C_{19}H_{14}N_2O_2$: C, 75.48; H, 4.67; N, 9.27. Found: C, 75.36; H, 4.48; N, 9.16.

1-Ethyl-3-(2-oxo-2-(pyridin-2-yl)ethylidene)indolin-2-one (10l).

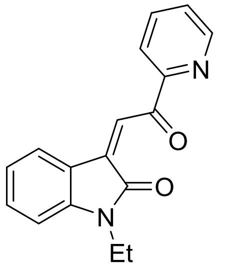

Red crystals; Yield 550 mg (66%); Mp 142 – 143 °C.

^{1}H NMR (500 MHz, DMSO-*d*$_6$) δ 8.83 (ddd, *J* = 4.7, 1.5, 0.9 Hz, 1H), 8.54 (d, *J* = 7.2 Hz, 1H), 8.45 (s, 1H), 8.18 (dd, *J* = 6.9, 0.9 Hz, 1H), 8.11 (td, *J* = 7.7, 1.7 Hz, 1H), 7.75 (ddd, *J* = 7.5, 4.7, 1.2 Hz, 1H), 7.48 (td, *J* = 7.7, 1.1 Hz, 1H), 7.14 (d, *J* = 7.8 Hz, 1H), 7.10 (td, *J* = 7.7, 0.9 Hz, 1H), 3.79 (q, *J* = 7.2 Hz, 2H), 1.20 (t, *J* = 7.2 Hz, 3H); ^{13}C NMR (126 MHz, DMSO-*d*$_6$) δ 190.1, 166.7, 153.4, 149.3, 145.4, 137.9, 136.7, 133.4, 128.1, 127.6, 124.7, 122.5, 122.2, 119.7, 109.1, 34.3, 12.5.

IR(DRA): 491, 547, 573, 603, 616, 640, 697, 745, 784, 807, 819, 859, 885, 911, 940, 996, 1016, 1083, 1104, 1132, 1156, 1227, 1294, 1351, 1387, 1433, 1481, 1580, 1597, 1619, 1669, 1705, 1919, 2934, 2973, 3049, 3116 cm^{-1}.

Anal. Calcd. for $C_{17}H_{14}N_2O_2$: C, 73.37; H, 5.07; N, 10.07. Found: C, 73.13; H, 5.03; N, 10.08.

1-Ethyl-3-(2-oxo-2-(pyridin-4-yl)ethylidene)indolin-2-one (10m).

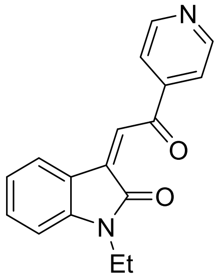

Dark-red crystals; Yield 560 mg (67%); Mp 144 – 145 °C.

^{1}H NMR (400 MHz, DMSO-d_6) δ 8.88 (dd, *J* = 4.4, 1.6 Hz, 2H), 8.23 (d, *J* = 7.7 Hz, 1H), 7.94 (dd, *J* = 4.4, 1.6 Hz, 2H), 7.75 (s, 1H), 7.53 – 7.44 (m, 1H), 7.14 (d, *J* = 7.9 Hz, 1H), 7.10 – 7.03 (m, 1H), 3.79 (q, *J* = 7.2 Hz, 2H), 1.19 (t, *J* = 7.2 Hz, 3H); ^{13}C NMR (126 MHz, DMSO-d_6) δ 190.7, 166.3, 151.0, 145.3, 143.0, 136.9, 133.6, 127.0, 124.7, 122.3, 121.4, 119.3, 109.3, 34.3, 12.5.

IR(DRA): 469, 490, 548, 574, 602, 630, 664, 692, 728, 746, 783, 835, 869, 887, 947, 993, 1027, 1072, 1110, 1135, 1160, 1230, 1293, 1354, 1406, 1469, 1482, 1554, 1598, 1620, 1665, 1709, 1949, 2979, 3046 cm^{-1}.

Anal. Calcd. for $C_{17}H_{14}N_2O_2$: C, 73.37; H, 5.07; N, 10.07. Found: C, 73.15; H, 5.03; N, 10.10.

5-Bromo-1-ethyl-3-(2-oxo-2-phenylethylidene)indolin-2-one (10n).

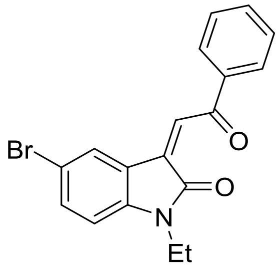

Red crystals; Yield 560 mg (52%); Mp 118 – 119 °C.

^{1}H NMR (500 MHz, DMSO-d_6) δ 8.25 (d, *J* = 1.9 Hz, 1H), 8.09 (d, *J* = 7.3 Hz, 2H), 7.86 (s, 1H), 7.76 – 7.73 (m, 1H), 7.67 – 7.59 (m, 3H), 7.12 (d, *J* = 8.4 Hz, 1H), 3.78 (q, *J* = 7.2 Hz, 2H), 1.18 (t, *J* = 7.2 Hz, 3H); ^{13}C NMR (126 MHz, DMSO-d_6) δ 190.9, 166.0, 144.1, 136.8, 135.0, 134.6, 134.3, 129.1, 128.9, 128.7, 127.8, 121.3, 113.8, 111.2, 34.5, 12.4.

IR(DRA): 472, 533, 587, 612, 683, 726, 788, 811, 893, 907, 938, 1007, 1073, 1089, 1114, 1140, 1185, 1230, 1272, 1290, 1342, 1365, 1446, 1458, 1468, 1578, 1598, 1615, 1666, 1706, 1809, 1840, 1864, 1896, 1958, 1977, 2350, 2728, 2875, 2946, 2970, 3039, 3066, 3115, 3392 cm^{-1}.

Anal. Calcd. for $C_{18}H_{14}BrNO_2$: C, 60.69; H, 3.96; N, 3.93. Found: C, 60.49; H, 3.78; N, 3.92.

5,7-Dibromo-1-hexyl-3-(2-oxo-2-phenylethylidene)indolin-2-one (10o).

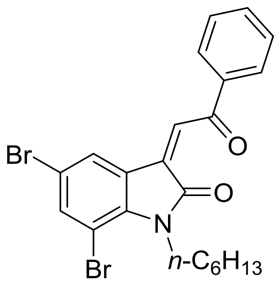

Orange powder; Yield 780 mg (64%); Mp 100 – 101 °C.

^{1}H NMR (500 MHz, DMSO-d_6) δ 8.14 (d, *J* = 1.9 Hz, 1H), 8.08 (dd, *J* = 8.3, 1.1 Hz, 2H), 7.93 (s, 1H), 7.85 (d, *J* = 1.9 Hz, 1H), 7.78 – 7.72 (m, 1H), 7.66 – 7.59 (m, 1H), 4.09 – 4.02 (m, 1H), 1.71 – 1.57 (m, 2H), 1.41 – 1.24 (m, 6H), 0.86 (t, *J* = 7.0 Hz, 3H); ^{13}C NMR (126 MHz, DMSO-d_6) δ 191.3, 167.0, 141.4, 138.8, 136.5, 134.5, 132.4, 130.2, 129.2, 128.8, 127.7, 124.2, 114.1, 102.8, 41.1, 30.8, 29.1, 25.5, 21.9, 13.8.

IR(DRA): 455, 500, 553, 567, 584, 617, 627, 660, 689, 699, 735, 753, 789, 829, 893, 960, 976, 999, 1039, 1053, 1066, 1091, 1149, 1192, 1218, 1248, 1281, 1302, 1342, 1365, 1403, 1421, 1440, 1467, 1495, 1523, 1572, 1595, 1658, 1744, 1797, 1874, 1933, 1952, 2852, 2928, 2953, 3021, 3076 cm^{-1};

Anal. Calcd. for $C_{22}H_{21}Br_2NO_2$: C, 53.79; H, 4.31; N, 2.85. Found: C, 53.61; H, 4.10; N, 2.81.

Thieno[2,3-*b*]indoles 12

8-Ethyl-2-(4-fluorophenyl)-8*H*-thieno[2,3-*b*]indole (12b).

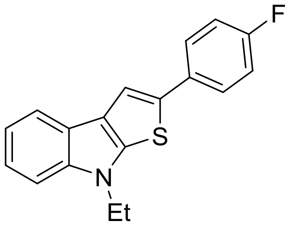

White needles; Yield 590 mg (90%); Mp 110 – 111 °C.

^{1}H NMR (500 MHz, DMSO-d_6) δ 7.88 (s, 1H), 7.82 (d, *J* = 7.7 Hz, 1H), 7.74 – 7.65 (m, 2H), 7.59 (d, *J* = 8.3 Hz, 1H), 7.30 – 7.23 (m, 3H), 7.20 – 7.13 (m, 1H), 4.33 (q, *J* = 7.2 Hz, 2H), 1.40 (t, *J* = 7.2 Hz, 3H); ^{13}C NMR (126 MHz, DMSO-d_6) δ 161.1 (d, J_{CF} = 244.1 Hz), 141.5, 140.7, 133.7, 131.6 (d, J_{CF} = 3.1 Hz), 126.4 (d, J_{CF} = 8.0 Hz), 123.4, 122.0, 121.5, 119.4, 119.1, 116.0 (d, J_{CF} = 21.8 Hz), 114.9, 109.9, 40.3, 13.6.

IR(DRA): 457, 498, 531, 549, 624, 667, 684, 745, 776, 802, 823, 837, 854, 927, 939, 1010, 1054, 1080, 1097, 1132, 1162, 1225, 1252, 1282, 1298, 1334, 1379, 1395, 1417, 1453, 1480, 1529, 1592, 1612, 1672, 1773, 1879, 1924, 2877, 2941, 2987, 3038, 3071 cm^{-1}.

MS (+APCI): Calcd. for $C_{18}H_{14}FNS$ m/z 296.0904 (M+H), found m/z 296.0902 (M+H).

2-(4-Bromophenyl)-8-ethyl-8*H*-thieno[2,3-*b*]indole (12c).

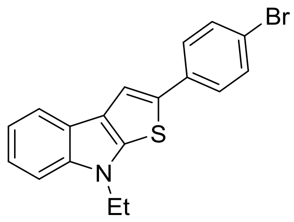

Pale yellow needles; Yield 670 mg (94%); Mp 142 – 143 °C.

^{1}H NMR (500 MHz, DMSO-d_6) δ 7.99 (s, 1H), 7.82 (d, *J* = 7.6 Hz, 1H), 7.68 – 7.56 (m, 5H), 7.28 (ddd, *J* = 8.3, 7.2, 1.2 Hz, 1H), 7.17 (td, *J* = 7.6, 0.9 Hz, 1H), 4.35 (q, *J* = 7.2 Hz, 2H), 1.41 (t, *J* = 7.2 Hz, 3H); ^{13}C NMR (126 MHz, DMSO-d_6) δ 141.8, 140.8, 134.3, 133.3, 131.9, 126.3, 123.5, 122.2, 121.5, 119.5, 119.2, 119.2, 115.6, 110.0, 40.4, 13.6.

IR(DRA): 478, 548, 582, 663, 683, 702, 744, 777, 814, 850, 926, 941, 1002, 1015, 1055, 1070, 1098, 1112, 1131, 1162, 1183, 1203, 1251, 1278, 1334, 1378, 1393, 1414, 1455, 1473, 1523, 1557, 1573, 1647, 1770, 1886, 1924, 2875, 2937, 2984, 3021, 3055 cm^{-1}.

MS (+APCI): Calcd. for $C_{18}H_{14}BrNS$ m/z 356.0103 (M+H), found m/z 356.0100 (M+H).

8-Ethyl-2-(*p*-tolyl)-8*H*-thieno[2,3-*b*]indole (12d).

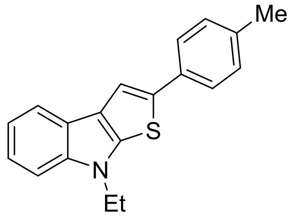

White needles; Yield 560 mg (79%); Mp 102 – 103 °C.

^{1}H NMR (500 MHz, DMSO-d_6) δ 7.85 (s, 1H), 7.81 (d, *J* = 7.7 Hz, 1H), 7.59 (d, *J* = 8.3 Hz, 1H), 7.56 (d, *J* = 8.0 Hz, 2H), 7.28 – 7.24 (m, 1H), 7.22 (d, *J* = 8.0 Hz, 2H), 7.19 – 7.12 (m, 1H), 4.33 (q, *J* = 7.2 Hz, 2H), 2.31 (s, 3H), 1.40 (t, *J* = 7.2 Hz, 3H); ^{13}C NMR (126 MHz, DMSO-d_6) δ 141.2, 140.7, 136.0, 135.1, 132.3, 129.7, 124.4, 123.4, 121.9, 121.5, 119.3, 119.1, 114.1, 109.9, 40.3, 20.7, 13.6.

IR(DRA): 484, 533, 552, 611, 627, 683, 739, 750 , 779, 810, 833, 853, 922, 943, 1014, 1031, 1055, 1086, 1120, 1132, 1162, 1184, 1204, 1253, 1282, 1335, 1380, 1396, 1419, 1440, 1466, 1481, 1507, 1531, 1565, 1596, 1644, 1762, 1797, 1841, 1874, 1893, 1910, 2731, 2876, 2937, 2981, 3018 cm^{-1}.

MS (+APCI): Calcd. for $C_{19}H_{17}NS$ m/z 292.1154 (M+H), found m/z 292.1153 (M+H).

2-(4-Cyanophenyl)-8-ethyl-8*H*-thieno[2,3-*b*]indole (12e).

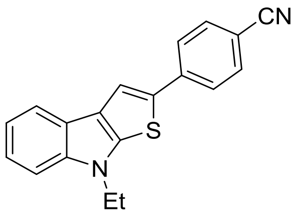

Yellow needles; Yield 555 mg (92%); Mp 150 – 151 °C.

^{1}H NMR (500 MHz, DMSO-d_6) δ 8.19 (s, 1H), 7.86 – 7.79 (m, 5H), 7.62 (d, *J* = 8.3 Hz, 1H), 7.34 – 7.27 (m, 1H), 7.23 – 7.17 (m, 1H), 4.36 (q, *J* = 7.2 Hz, 2H), 1.41 (t, *J* = 7.2 Hz, 3H); ^{13}C NMR (126 MHz, DMSO-d_6) δ 143.2, 141.1, 139.6, 133.0, 132.5, 124.5, 123.9, 122.6, 121.5, 119.8, 119.3, 119.0, 117.8, 110.1, 108.0, 40.4, 13.5.

IR(DRA): 501, 528, 556, 640, 668, 687, 749, 777, 813, 830, 851, 942, 961, 1017, 1053, 1084, 1109, 1131, 1178, 1203, 1254, 1278, 1330, 1353, 1401, 1418, 1453, 1469, 1504, 1525, 1553, 1573, 1600, 1657, 1883, 2168, 2220, 2417, 2574, 2730, 2879, 2942, 2981, 3057, 3077, 3197, 3396, 3819 cm^{-1}.

MS (+ESI): Calcd. for $C_{19}H_{14}N_2S$ m/z 627,1648 (2M+Na), found m/z 627.1653 (2M+Na).

2-(3,4-Dimethoxyphenyl)-8-ethyl-8*H*-thieno[2,3-*b*]indole (12g).

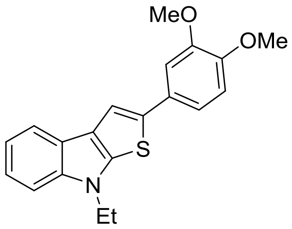

White powder; Yield 375 mg (56%); Mp 108 – 109 °C.

^{1}H NMR (500 MHz, DMSO-d_6) δ 7.83 (s, 1H), 7.80 (d, *J* = 7.8 Hz, 1H), 7.58 (d, *J* = 8.3 Hz, 1H), 7.35 – 7.22 (m, 2H), 7.20 – 7.11 (m, 2H), 6.97 (d, *J* = 8.3 Hz, 1H), 4.33 (q, *J* = 7.2 Hz, 2H), 3.87 (s, 3H), 3.78 (s, 3H), 1.41 (t, *J* = 7.2 Hz, 3H); ^{13}C NMR* (126 MHz, DMSO-d_6) δ 149.2, 148.0, 141.0, 140.6, 135.3, 128.0, 123.3, 121.8, 121.5, 119.3, 119.0, 116.9, 113.7, 112.3, 109.9, 108.6, 55.6, 40.3, 13.6.

*The signals from non-equivalent carbon atoms of MeO-groups have substantially identical chemical shift and appear as a single peak at 55.6 ppm.

IR(DRA): 497, 521, 552, 568, 624, 637, 668, 687, 737, 763, 777, 797, 806, 822, 848, 883, 919, 950, 1001, 1023, 1054, 1082, 1146, 1165, 1187, 1203, 1258, 1288, 1336, 1383, 1396, 1416, 1445, 1483, 1508, 1532, 1583, 1604, 1641, 1752, 1826, 1875, 1915, 2019, 2580, 2834, 2932, 2978, 3002, 3050, 3081 cm^{-1}.

MS (+APCI): Calcd. for $C_{20}H_{19}NO_2S$ m/z 338.1209 (M+H), found m/z 338.1211 (M+H).

8-Ethyl-2-(3,4,5-trimethoxyphenyl)-8*H*-thieno[2,3-*b*]indole (12h).

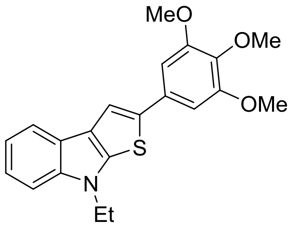

White powder; Yield 515 mg (70%); Mp 104 – 105 °C.

^{1}H NMR (500 MHz, DMSO-d_6) δ 7.93 (s, 1H), 7.81 (d, J = 7.7 Hz, 1H), 7.60 (d, J = 8.2 Hz, 1H), 7.30 – 7.23 (m, 1H), 7.20 – 7.13 (m, 1H), 6.95 (s, 2H), 4.35 (q, J = 7.2 Hz, 2H), 3.88 (s, 6H), 3.69 (s, 3H), 1.42 (t, J = 7.2 Hz, 3H); ^{13}C NMR (126 MHz, DMSO-d_6) δ 153.3, 141.4, 140.7, 136.6, 135.0, 130.8, 123.3, 121.9, 121.5, 119.4, 119.0, 114.8, 109.9, 102.1, 60.1, 56.0, 40.3, 13.6.

IR(DRA): 502, 531, 579, 629, 648, 665, 697, 733, 752, 777, 802, 813, 919, 1002, 1013, 1025, 1053, 1081, 1128, 1205, 1162, 1245, 1318, 1275, 1337, 1378, 1423, 1464, 1484, 1507, 1529, 1579, 1996, 2833, 2940, 2973, 3056 cm^{-1};

MS (+APCI): Calcd. for $C_{21}H_{21}NO_3S$ m/z 368.1315 (M+H), found m/z 368.1317 (M+H).

8-Ethyl-2-(perfluorophenyl)-8*H*-thieno[2,3-*b*]indole (12i).

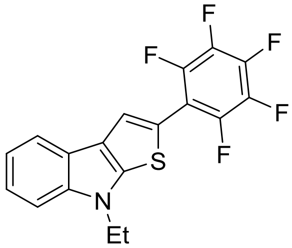

White needles; Yield 565 mg (77%); Mp 176 – 177 °C.

^{1}H NMR (500 MHz, DMSO-d_6) δ 8.02 (s, 1H), 7.94 (d, J = 7.7 Hz, 1H), 7.65 (d, J = 8.3 Hz, 1H), 7.35 – 7.30 (m, 1H), 7.24 – 7.17 (m, 1H), 4.40 (q, J = 7.2 Hz, 2H), 1.42 (t, J = 7.2 Hz, 3H); ^{13}C NMR (101 MHz, DMSO-d_6) δ 144.1, 143.2 (dm*, J_{CF} = 247.3 Hz), 141.1, 138.4 (dm*, J_{CF} = 250.6 Hz), 137.4 (dm*, J_{CF} = 250.5 Hz), 122.7, 122.4, 122.1 – 121.8 (m), 121.1, 119.5, 119.2, 115.5, 110.6 – 110.2 (m), 109.7, 40.3, 13.1.

*dm = doublet of multiplets.

IR(DRA): 509, 550, 564, 636, 679, 745, 780, 796, 840, 880, 926, 980, 1015, 1032, 1058, 1087, 1128, 1142, 1166, 1213, 1255, 1280, 1330, 1355, 1380, 1402, 1429, 1478, 1497, 1525, 1610, 1680, 1886, 1924, 2946, 2989, 3055, 3120 cm^{-1}.

MS (+APCI): Calcd. for $C_{18}H_{10}F_5NS$ m/z 367.0449 (M), found m/z 367.0449 (M).

2-(4-Bromothiophen-2-yl)-8-ethyl-8*H*-thieno[2,3-*b*]indole (12j).

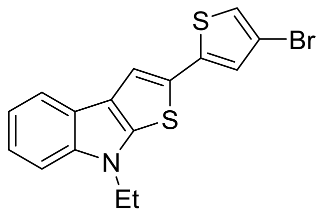

Yellow powder; Yield 545 mg (75%); Mp 114 – 115 °C.

^{1}H NMR (400 MHz, DMSO-d_6) δ 7.83 (d, *J* = 7.7 Hz, 1H), 7.79 (s, 1H), 7.61 (d, *J* = 8.3 Hz, 1H), 7.57 (d, *J* = 1.4 Hz, 1H), 7.33 – 7.25 (m, 2H), 7.21 – 7.13 (m, 1H), 4.35 (q, *J* = 7.2 Hz, 2H), 1.40 (t, *J* = 7.2 Hz, 3H); ^{13}C NMR (126 MHz, DMSO-d_6) δ 141.6, 140.9, 139.7, 126.3, 124.1, 123.1, 122.3, 121.3, 121.2, 119.6, 119.3, 116.2, 110.0, 109.5, 40.4, 13.5.

IR(DRA): 486, 542, 561, 586, 650, 675, 695, 725, 750, 777, 786, 807, 822, 833, 847, 863, 908, 926, 946, 1017, 1050, 1081, 1130, 1138, 1161, 1172, 1192, 1217, 1253, 1278, 1334, 1357, 1380, 1398, 1429, 1442, 1464, 1475, 1503, 1547, 1570, 1599, 1662, 1774, 1811, 1855, 1891, 1929, 2867, 2930, 2974, 3047, 3069, 3114 cm^{-1}.

Anal. Calcd. for $C_{16}H_{12}BrNS_2$: C, 53.04; H, 3.34; N, 3.87. Found: C, 52.93; H, 3.21; N, 3.70.

8-Ethyl-2-(thiophen-2-yl)-8*H*-thieno[2,3-*b*]indole (12k).

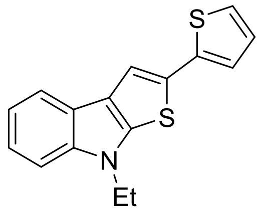

Cream powder; Yield 420 mg (74%); Mp 85 – 86 °C.

Analytical data of thieno[2,3-*b*]indole **12k** are identical to the reported data [2].

8-Ethyl-2-(pyridin-2-yl)-8*H*-thieno[2,3-*b*]indole (12l).

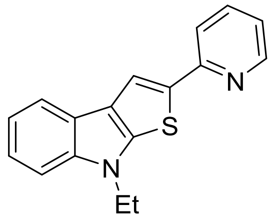

Pale yellow powder; Yield 340 mg (61%); Mp 83 – 84 °C.

^{1}H NMR (400 MHz, DMSO-d_6) δ 8.47 (d, *J* = 4.2 Hz, 1H), 8.22 (s, 1H), 7.94 (d, *J* = 8.1 Hz, 1H), 7.85 – 7.77 (m, 2H), 7.61 (d, *J* = 8.2 Hz, 1H), 7.31 – 7.25 (m, 1H), 7.22 – 7.14 (m, 2H), 4.37 (q, *J* = 7.2 Hz, 2H), 1.43 (t, *J* = 7.2 Hz, 3H); ^{13}C NMR (126 MHz, DMSO-d_6) δ 153.0,

149.0, 144.2, 141.0, 136.8, 136.5, 123.5, 122.2, 121.7, 121.2, 119.6, 119.1, 117.6, 116.7, 110.0, 40.3, 13.5.

IR(DRA): 475, 550, 573, 602, 619, 670, 705, 740, 775, 809, 842, 853, 876, 890, 945, 959, 991, 1016, 1050, 1087, 1131, 1154, 1207, 1251, 1281, 1292, 1331, 1383, 1412, 1430, 1455, 1479, 1494, 1527, 1583, 1609, 1675, 1764, 1849, 1884, 1920, 1970, 2875, 2933, 2971, 3048 cm^{-1}.

MS (+ESI): Calcd. for $C_{17}H_{14}N_2S$ m/z 279.0950 (M+H), found m/z 279.0949 (M+H).

8-Ethyl-2-(pyridin-4-yl)-8*H*-thieno[2,3-*b*]indole (12m).

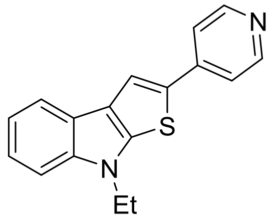

Pale yellow powder; Yield 335 mg (60%); Mp 140 – 141 °C.

^{1}H NMR (400 MHz, DMSO-d_6) δ 8.53 (dd, *J* = 4.7, 1.5 Hz, 2H), 8.29 (s, 1H), 7.86 (d, *J* = 7.7 Hz, 1H), 7.68 – 7.54 (m, 3H), 7.36 – 7.26 (m, 1H), 7.25 – 7.16 (m, 1H), 4.39 (q, *J* = 7.2 Hz, 2H), 1.43 (t, *J* = 7.2 Hz, 3H); ^{13}C NMR (126 MHz, DMSO-d_6) δ 150.2, 143.1, 142.0, 141.2, 131.4, 123.7, 122.6, 121.4, 119.8, 119.3, 118.3, 118.1, 110.2, 40.5, 13.5.

IR(DRA): 479, 550, 563, 661, 682, 694, 729, 774, 809, 844, 854, 867, 924, 941, 967, 991, 1018, 1052, 1077, 1133, 1163, 1219, 1254, 1280, 1325, 1348, 1378, 1406, 1454, 1466, 1481, 1496, 1521, 1538, 1573, 1593, 1668, 1930, 2934, 2980, 3054 cm^{-1}.

MS (+ESI): Calcd. for $C_{17}H_{14}N_2S$ m/z 279.0950 (M+H), found m/z 279.0953 (M+H).

5-Bromo-8-ethyl-2-phenyl-8*H*-thieno[2,3-*b*]indole (12n).

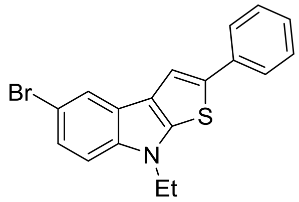

White needles; Yield 600 mg (84%); Mp 140 – 141 °C.

^{1}H NMR (500 MHz, DMSO-d_6) δ 8.05 (d, *J* = 2.0 Hz, 1H), 7.92 (s, 1H), 7.69 – 7.62 (m, 2H), 7.60 (d, *J* = 8.7 Hz, 1H), 7.47 – 7.41 (m, 2H), 7.38 (dd, *J* = 8.7, 2.0 Hz, 1H), 7.32 – 7.25 (m, 1H), 4.35 (q, *J* = 7.2 Hz, 2H), 1.40 (t, *J* = 7.2 Hz, 3H); ^{13}C NMR (126 MHz, DMSO-d_6) δ 142.7, 139.5, 135.5, 134.8, 129.2, 126.9, 124.6, 124.3, 123.0, 122.7, 121.5, 114.7, 112.0, 111.8, 40.6, 13.6.

IR(DRA): 473, 555, 578, 584, 621, 692, 740, 754, 779, 798, 827, 836, 860, 872, 904, 937, 962, 978, 1042, 1059, 1082, 1101, 1138, 1159, 1187, 1205, 1247, 1260, 1313, 1332, 1345, 1378,

1400, 1438, 1455, 1478, 1496, 1524, 1561, 1574, 1593, 1659, 1716, 1805, 1851, 1936, 1955, 2874, 2932, 2977, 3023, 3074 cm^{-1}.

MS (+APCI): Calcd. for $C_{18}H_{14}BrNS$ m/z 355.0025 (M), found m/z 355.0022 (M).

5,7-Dibromo-8-hexyl-2-phenyl-8*H*-thieno[2,3-*b*]indole (12o).

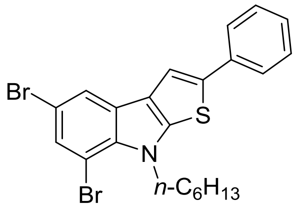

White needles; Yield 775 mg (79%); Mp 104 – 105 °C.

^{1}H NMR (500 MHz, DMSO-d_6) δ 8.11 (d, *J* = 1.8 Hz, 1H), 7.93 (s, 1H), 7.66 (d, *J* = 7.3 Hz, 2H), 7.61 (d, *J* = 1.8 Hz, 1H), 7.50 – 7.41 (m, 2H), 7.35 – 7.28 (m, 1H), 4.60 (t, *J* = 7.4 Hz, 2H), 1.90 – 1.79 (m, 2H), 1.36 – 1.23 (m, 6H), 0.83 (t, *J* = 7.0 Hz, 3H); ^{13}C NMR (126 MHz, DMSO-d_6) δ 145.9, 136.5, 135.6, 134.4, 129.3, 128.4, 127.3, 125.9, 124.7, 122.2, 121.4, 114.5, 111.9, 103.6, 48.2, 30.7, 30.1, 25.6, 21.9, 13.8.

IR(DRA): 455, 465, 500, 553, 567, 584, 617, 627, 660, 677, 689, 699, 735, 753, 789, 829, 875, 893, 960, 976, 999, 1039, 1053, 1066, 1091, 1122, 1149, 1192, 1218, 1248, 1281, 1302, 1331, 1342, 1365, 1403, 1421, 1440, 1467, 1485, 1495, 1523, 1544, 1572, 1595, 1658, 1744, 1797, 1874, 1933, 1952, 2852, 2928, 2953, 3021, 3076 cm^{-1}.

MS (+ESI): Calcd. for $C_{22}H_{21}Br_2NS$ m/z 489.9834 (M+H), found m/z 489.9838 (M+H).

References

1. Metwally, S. A.; Younes, M. I.; Abbas, H. H. *Acta Chim. Hungarica* **1989**, *126*, 591−598.

2. Irgashev, R. A.; Karmatsky, A. A.; Kozyukhin, S. A.; Ivanov, V. K.; Sadovnikov, A.; Kozik, V. V.; Grinberg, V. A.; Emets, V. V.; Rusinov, G. L.; Charushin, V. N. *Synth. Met.* **2015**, *199*, 152–158.

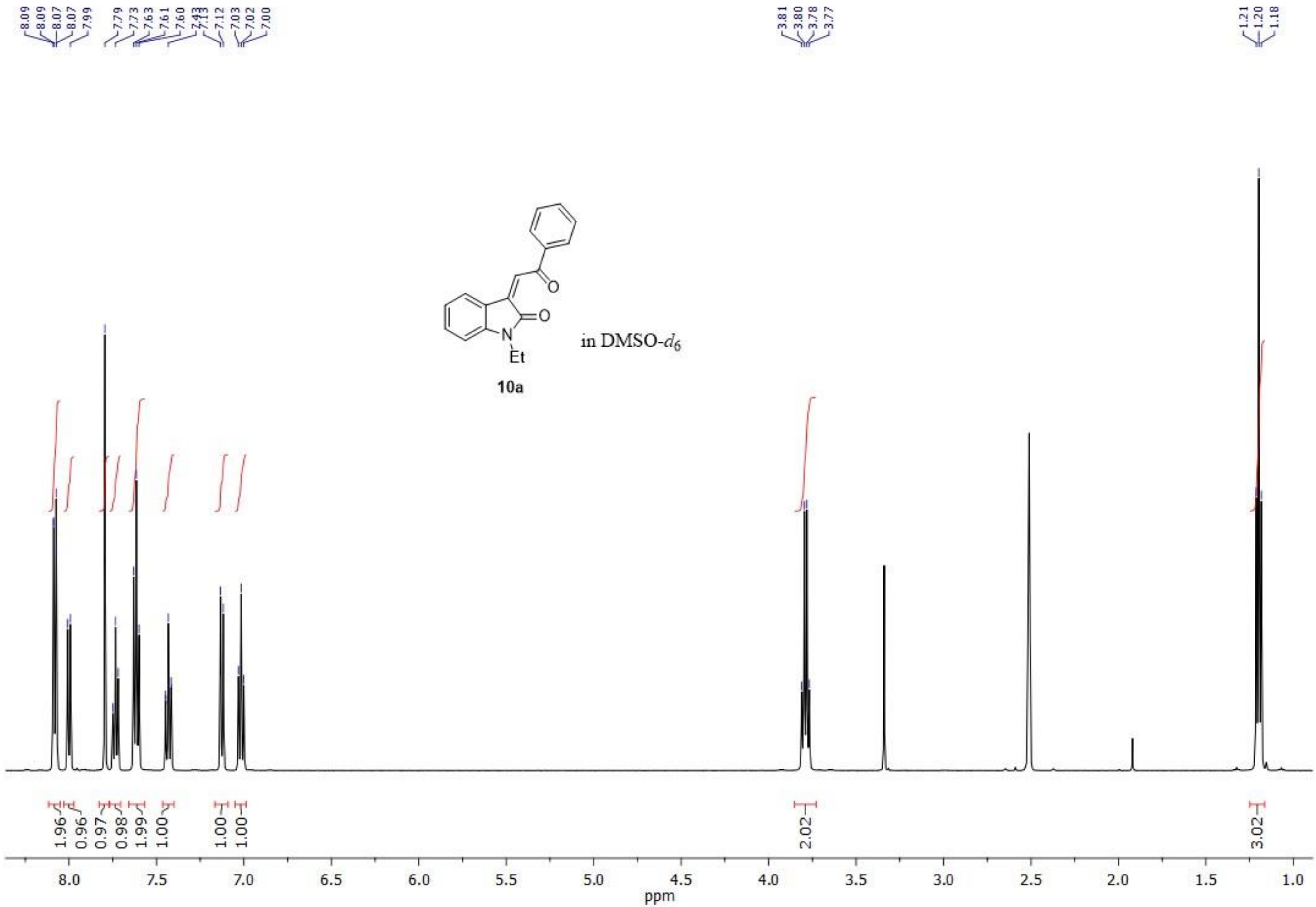
8.09
8.09
8.07
8.07
7.99
7.79
7.73
7.63
7.61
7.60
7.43
7.13
7.12
7.03
7.02
7.00
3.81
3.80
3.78
3.77
1.21
1.20
1.18
O
O
N
Et
10a
in DMSO-d_6
1.96
0.96
0.97
0.98
1.99
1.00
1.00
1.00
2.02
3.02
8.0
7.5
7.0
6.5
6.0
5.5
5.0
4.5
4.0
3.5
3.0
2.5
2.0
1.5
1.0
ppm

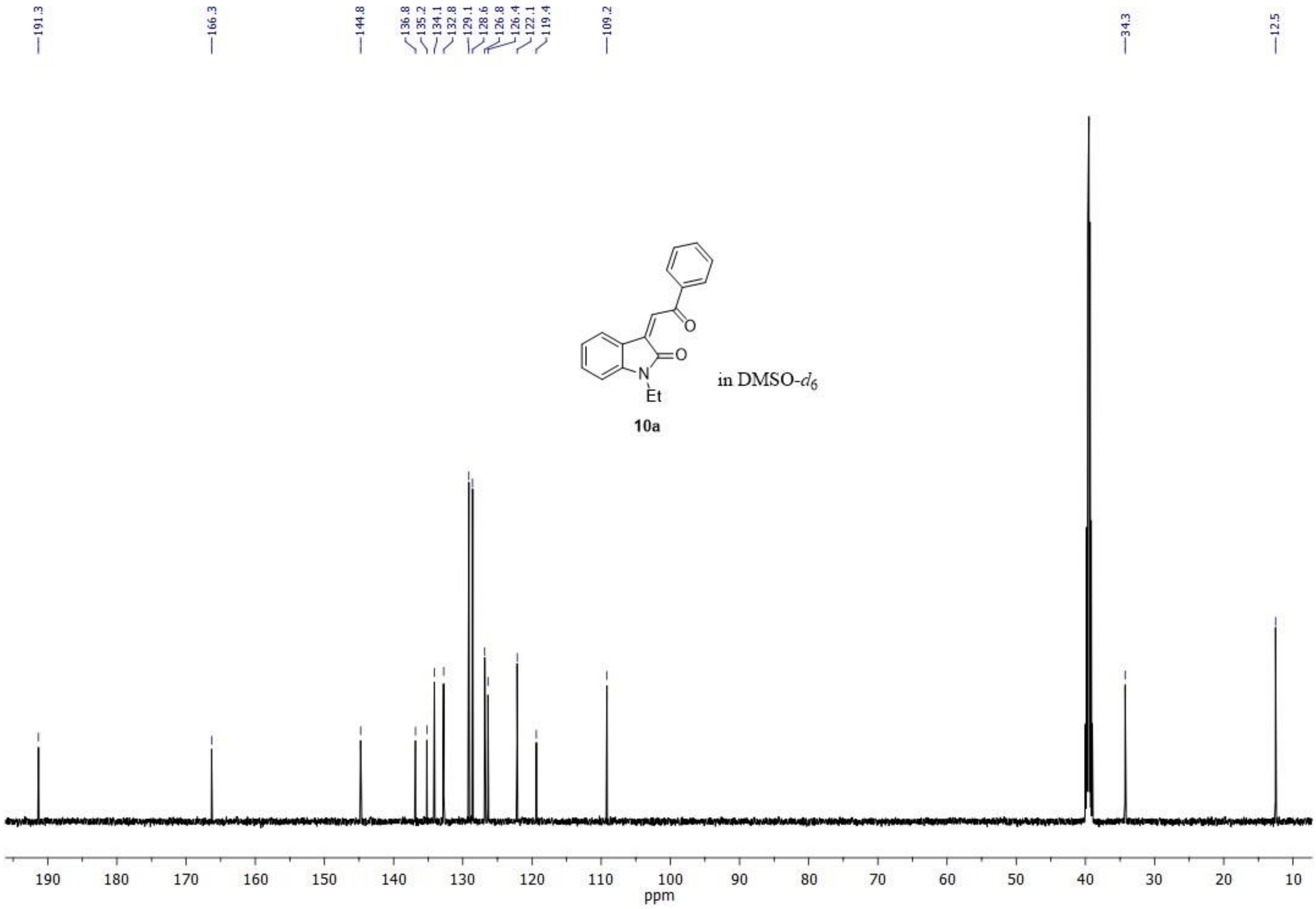
191.3
166.3
144.8
136.8
135.2
134.1
132.8
129.1
128.6
126.8
126.4
122.1
119.4
109.2
34.3
12.5
O
O
N
Et
10a
in DMSO-d_6
190 180 170 160 150 140 130 120 110 100 90 80 70 60 50 40 30 20 10
ppm

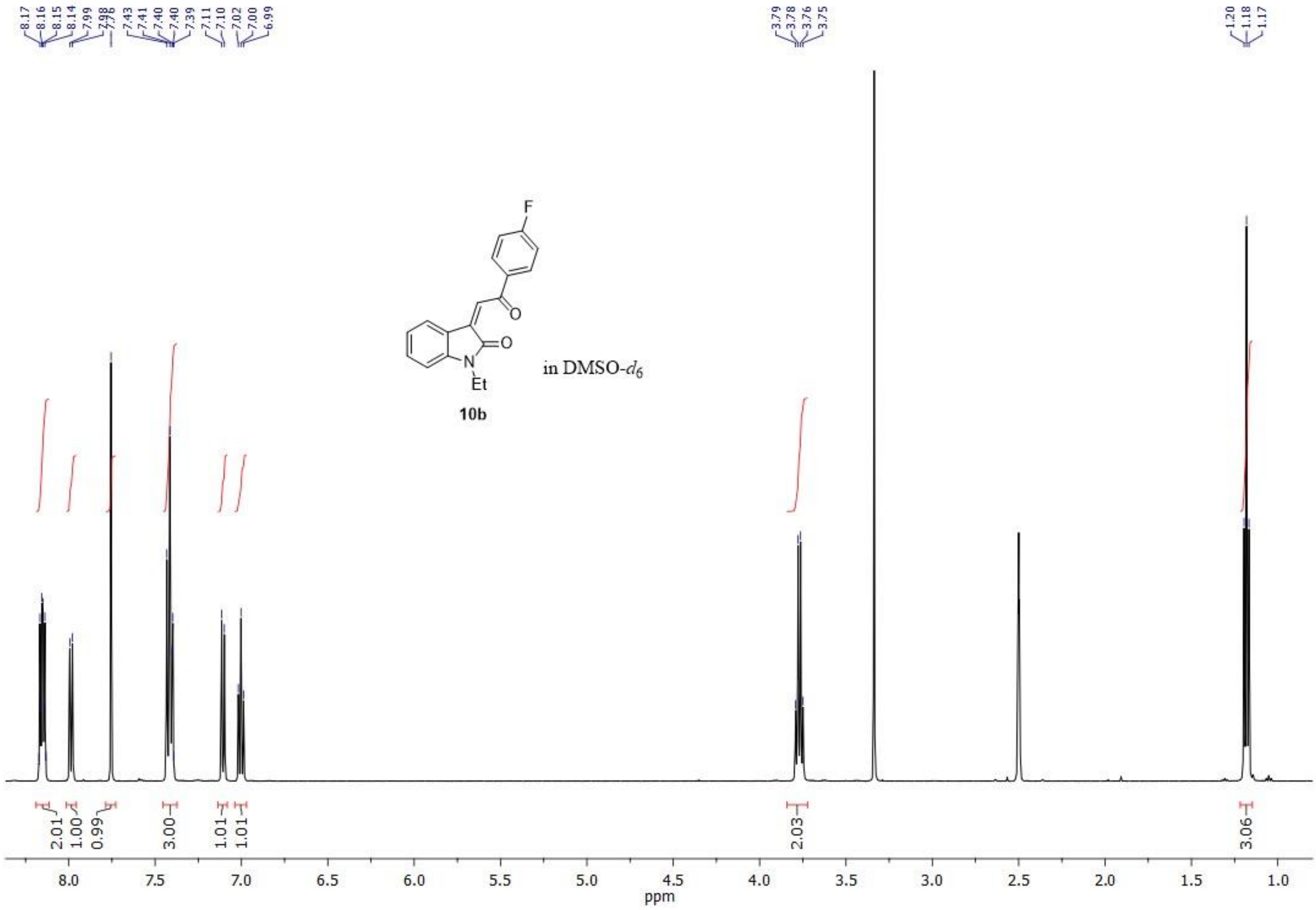
8.17
8.16
8.15
8.14
7.99
7.98
7.76
7.43
7.41
7.40
7.40
7.39
7.11
7.10
7.02
7.00
6.99
3.79
3.78
3.76
3.75
1.20
1.18
1.17
F
O
O
N
Et
10b
in DMSO-d_6
2.01
1.00
0.99
3.00
1.01
1.01
2.03
3.06
8.0
7.5
7.0
6.5
6.0
5.5
5.0
4.5
4.0
3.5
3.0
2.5
2.0
1.5
1.0
ppm

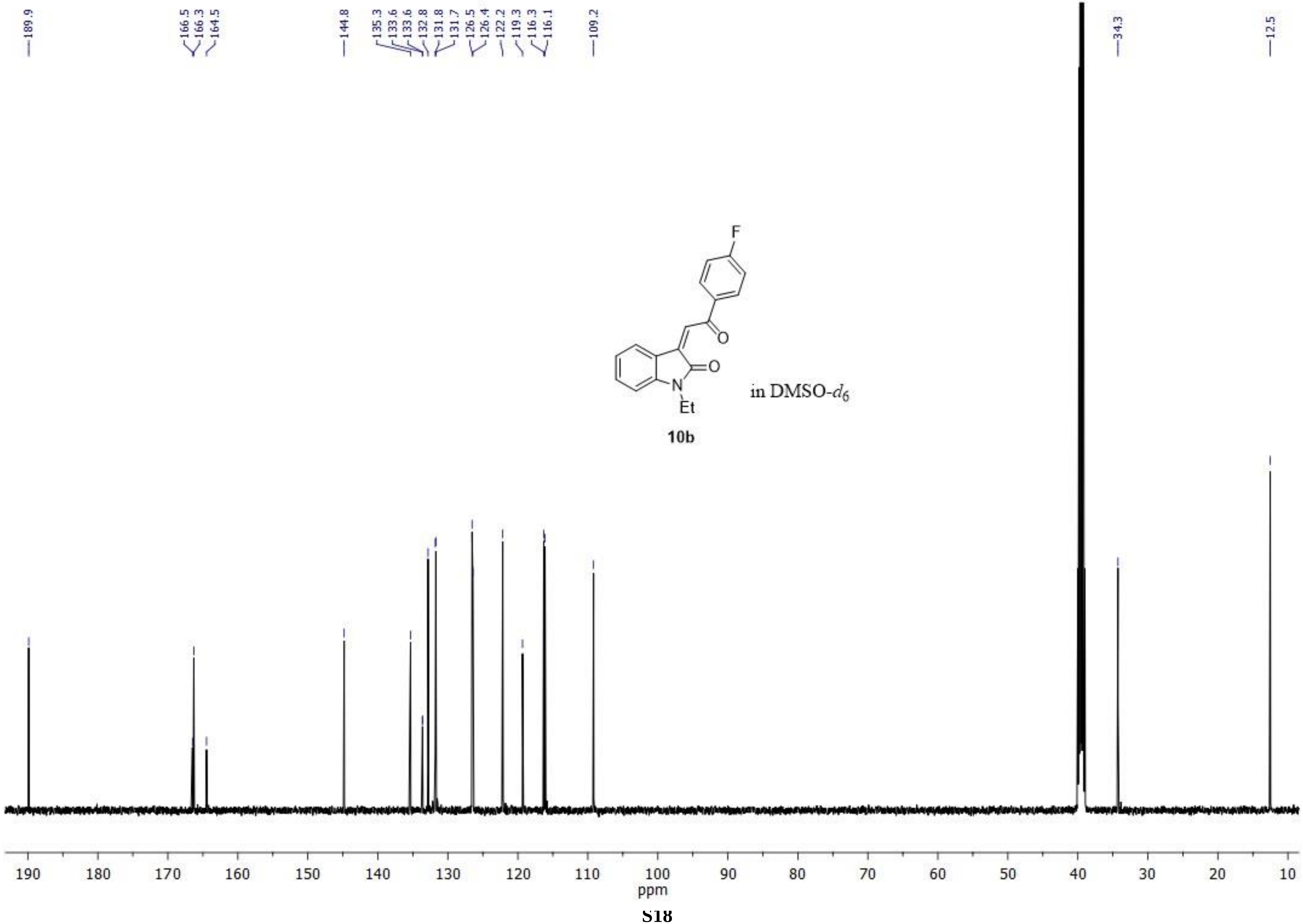
189.9
166.5
166.3
164.5
144.8
135.3
133.6
133.6
132.8
131.8
131.7
126.5
126.4
122.2
119.3
116.3
116.1
109.2
34.3
12.5
F
O
O
N
Et
10b
in DMSO-d_6
190 180 170 160 150 140 130 120 110 100 90 80 70 60 50 40 30 20 10
ppm

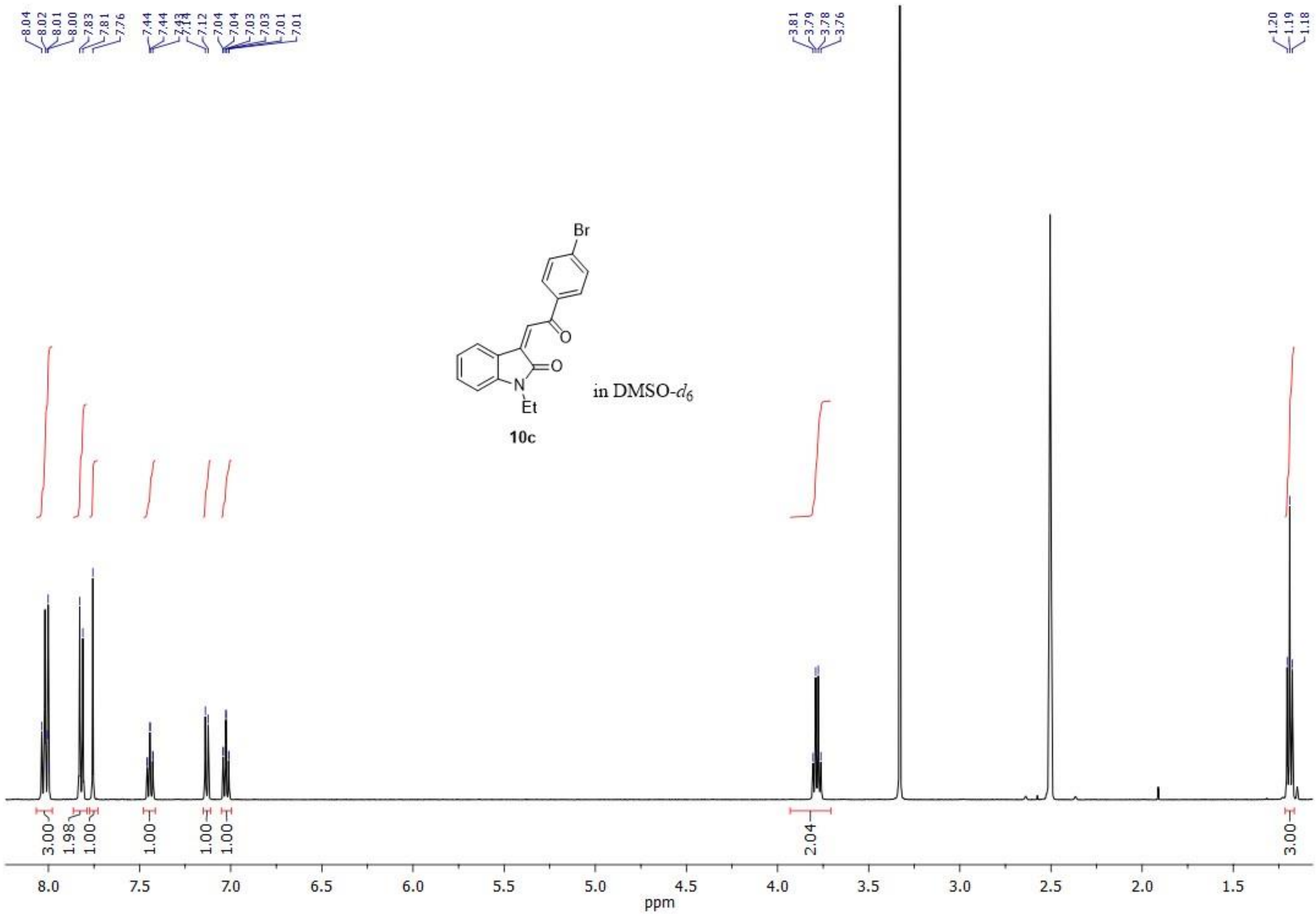
8.04
8.02
8.01
8.00
7.83
7.81
7.76
7.44
7.44
7.43
7.14
7.12
7.04
7.04
7.03
7.03
7.01
7.01
3.81
3.79
3.78
3.76
1.20
1.19
1.18
Br
O
O
N
Et
10c
in DMSO-d_6
3.00
1.98
1.00
1.00
1.00
1.00
2.04
3.00
8.0
7.5
7.0
6.5
6.0
5.5
5.0
4.5
4.0
3.5
3.0
2.5
2.0
1.5
ppm

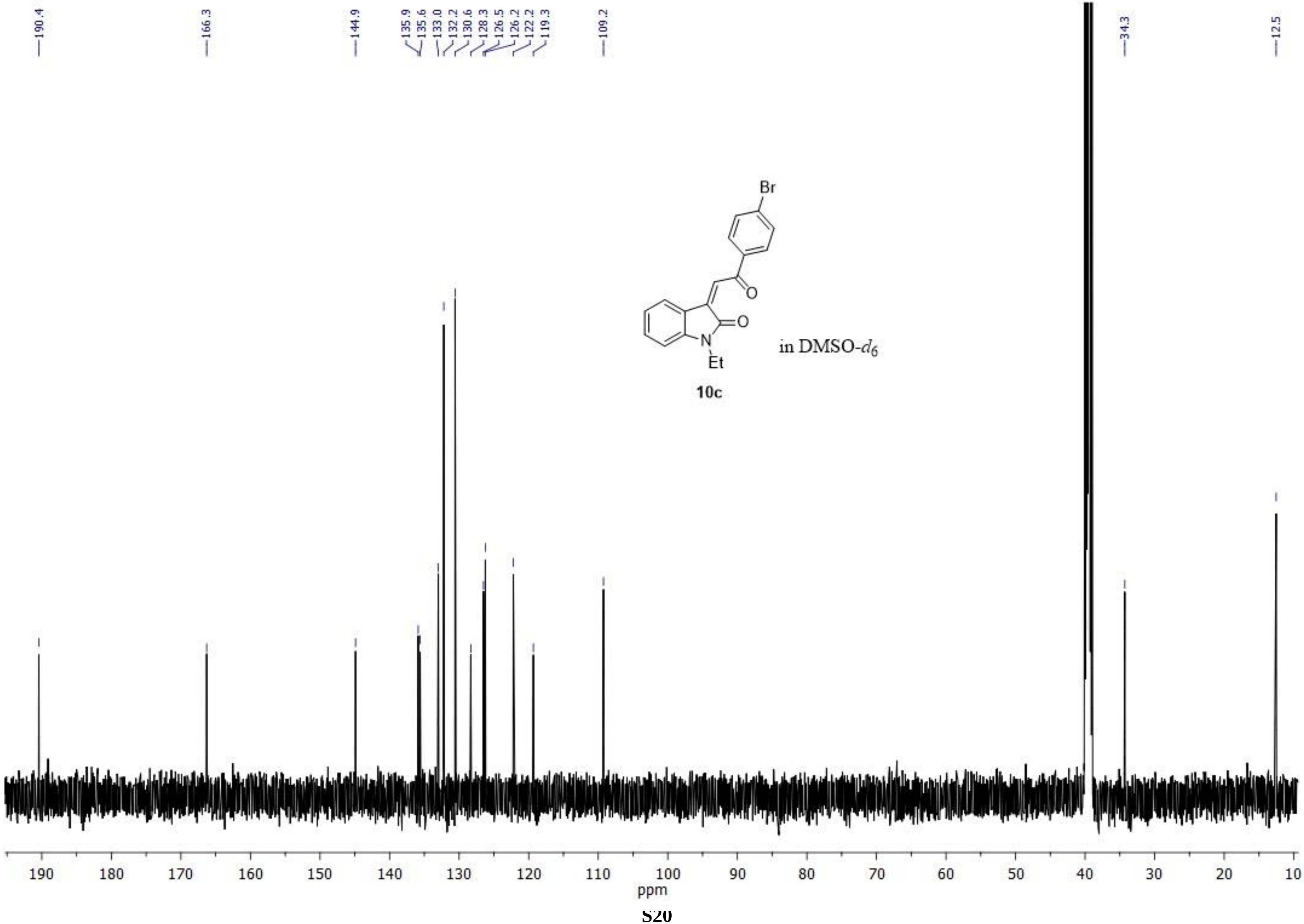
190.4
166.3
144.9
135.9
135.6
133.0
132.2
130.6
128.3
126.5
126.2
122.2
119.3
109.2
34.3
12.5
Br
O
O
N
Et
10c
in DMSO-d_6
190
180
170
160
150
140
130
120
110
100
90
80
70
60
50
40
30
20
10
ppm

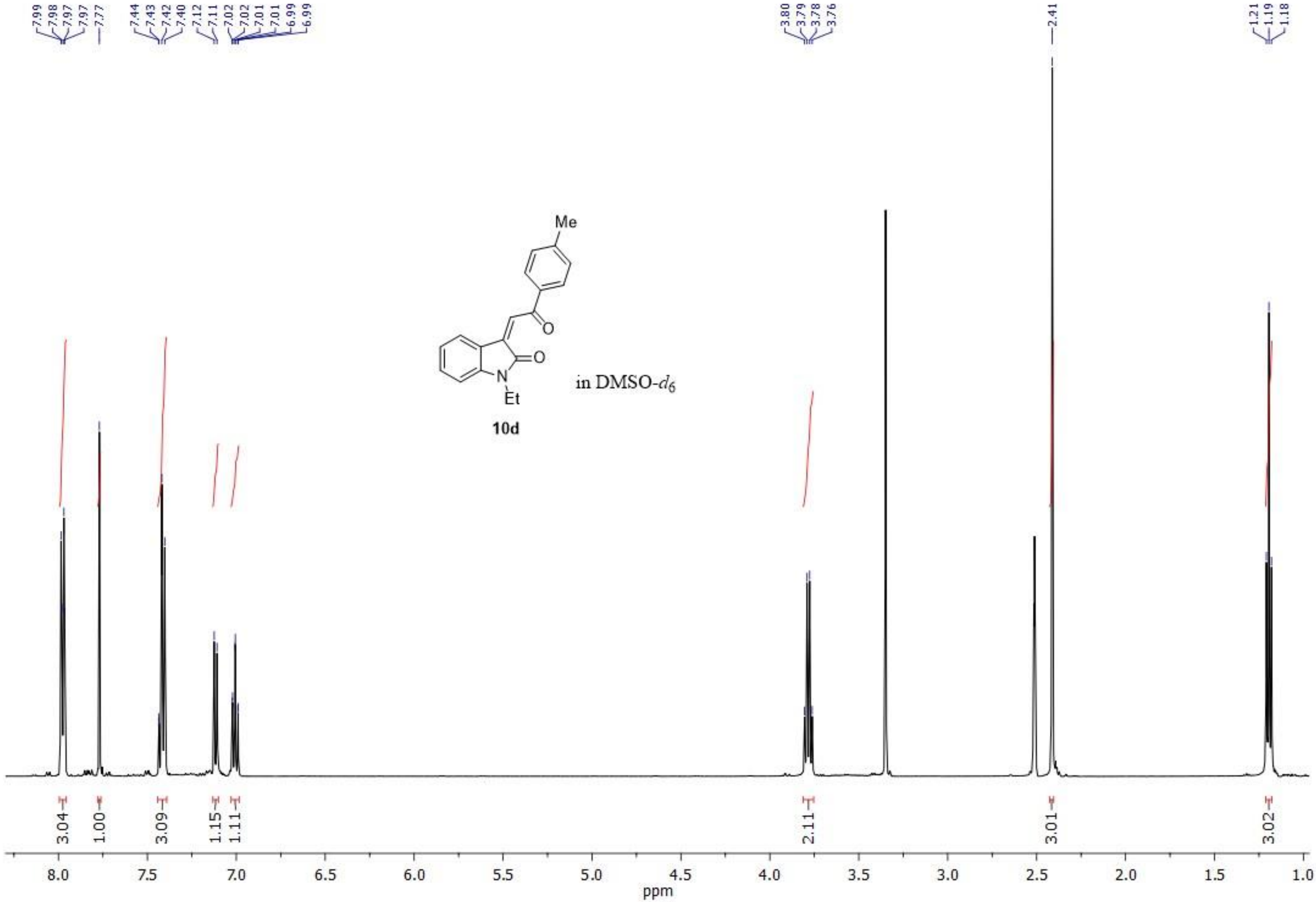
7.99
7.98
7.97
7.97
7.77
7.44
7.43
7.42
7.40
7.12
7.11
7.02
7.02
7.01
7.01
6.99
6.99
3.80
3.79
3.78
3.76
2.41
1.21
1.19
1.18
Me
O
O
N
Et
10d
in DMSO-d_6
3.04
1.00
3.09
1.15
1.11
2.11
3.01
3.02
8.0
7.5
7.0
6.5
6.0
5.5
5.0
4.5
4.0
3.5
3.0
2.5
2.0
1.5
1.0
ppm

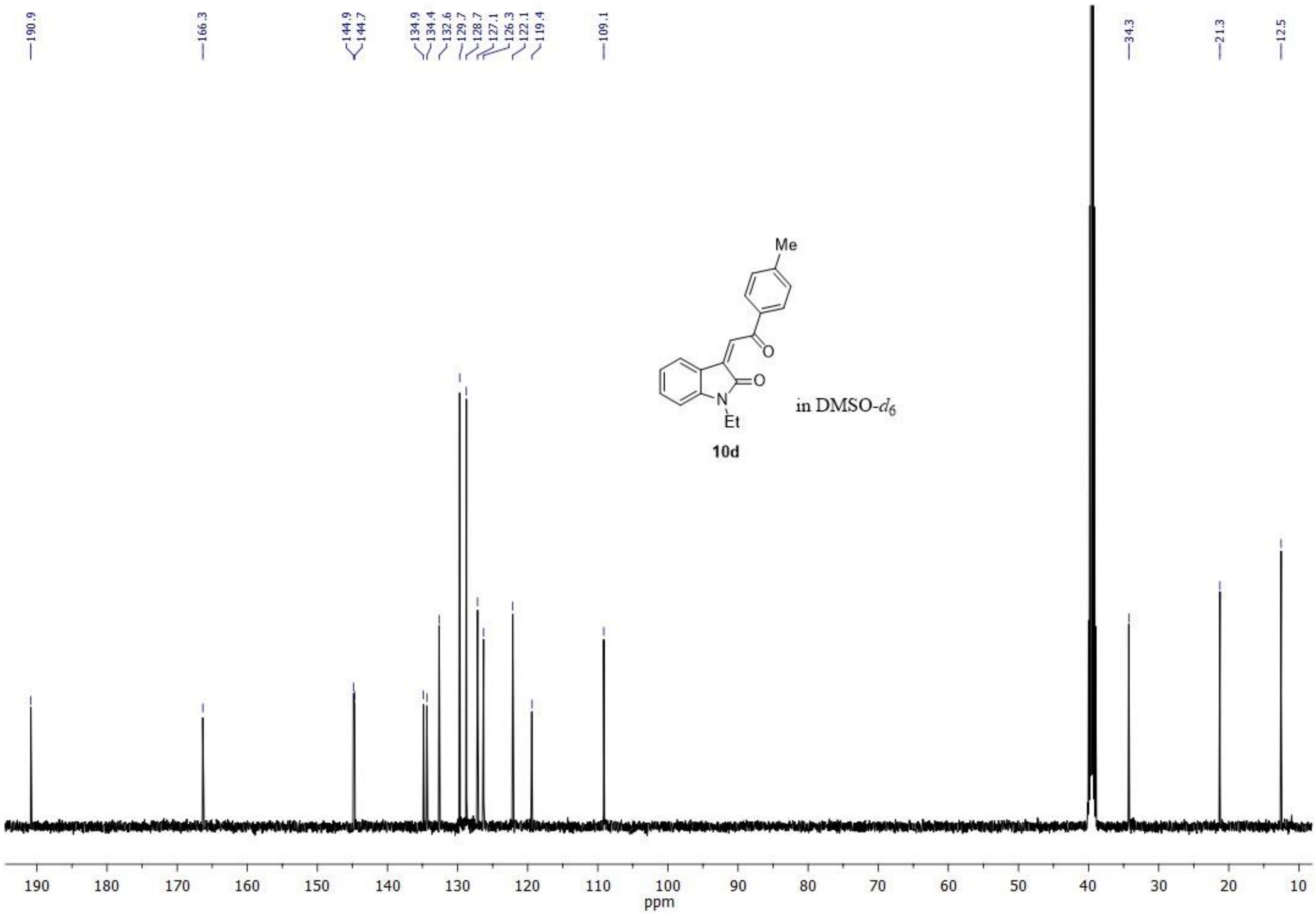
190.9
166.3
144.9
144.7
134.9
134.4
132.6
129.7
128.7
127.1
126.3
122.1
119.4
109.1
34.3
21.3
12.5
Me
O
O
N
Et
10d
in DMSO-d_6
190
180
170
160
150
140
130
120
110
100
90
80
70
60
50
40
30
20
10
ppm

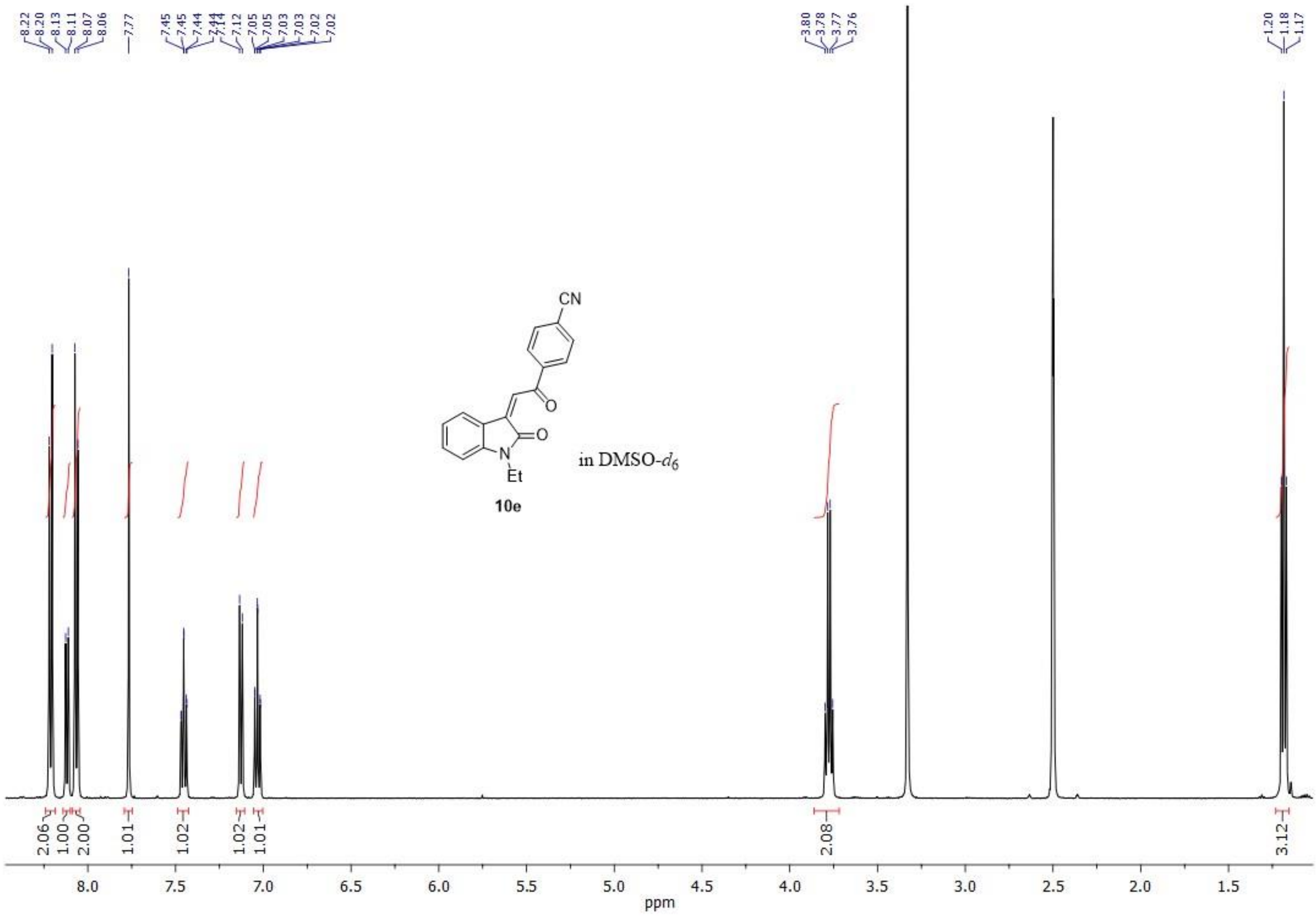
8.22
8.20
8.13
8.11
8.07
8.06
7.77
7.45
7.45
7.44
7.44
7.14
7.12
7.05
7.03
7.03
7.02
7.02
3.80
3.78
3.77
3.76
1.20
1.18
1.17
CN
O
O
N
Et
10e
in DMSO-d_6
2.06
1.00
2.00
1.01
1.02
1.02
1.01
2.08
3.12
8.0
7.5
7.0
6.5
6.0
5.5
5.0
4.5
4.0
3.5
3.0
2.5
2.0
1.5
ppm

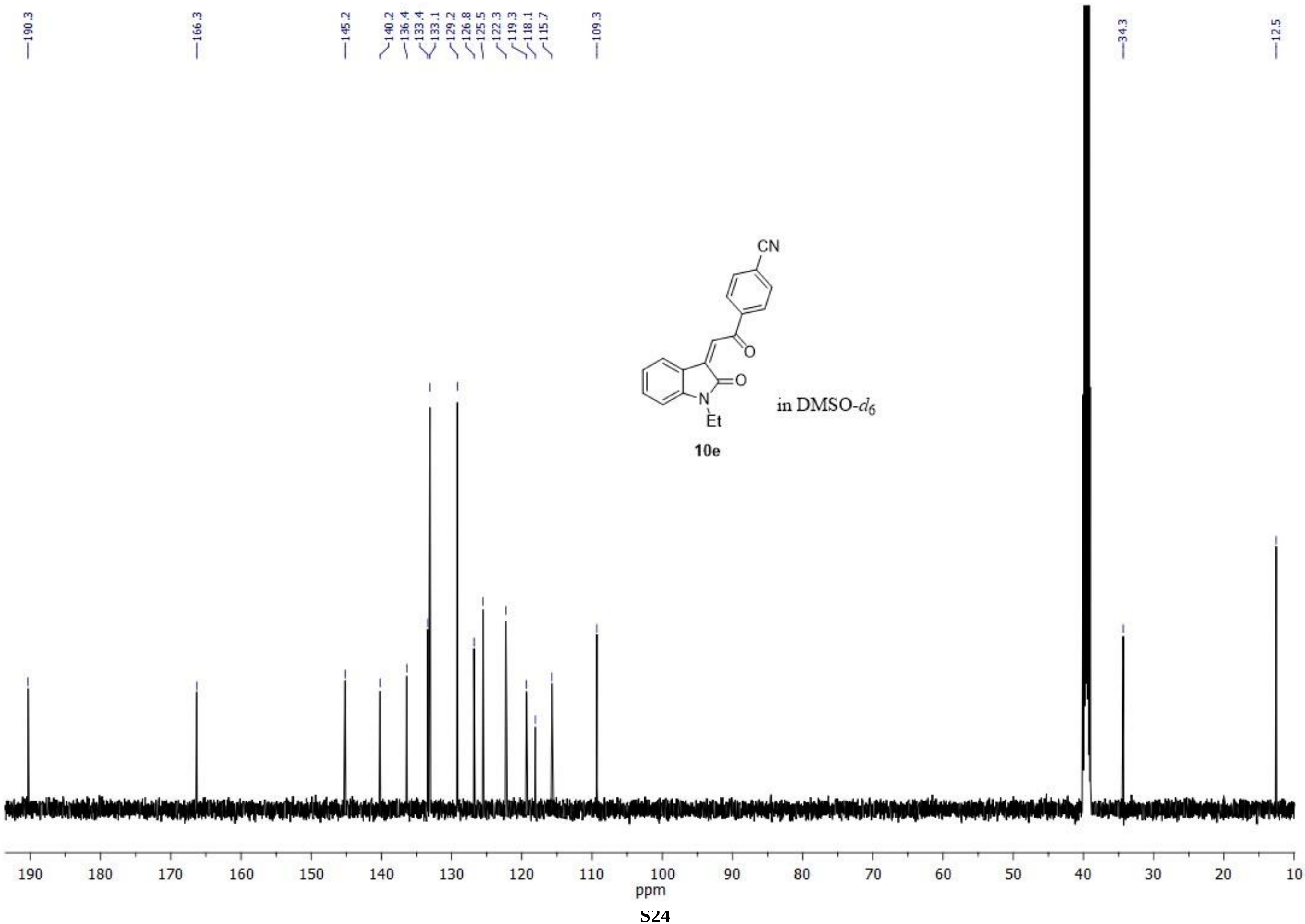
190.3
166.3
145.2
140.2
136.4
133.4
133.1
129.2
126.8
125.5
122.3
119.3
118.1
115.7
109.3
34.3
12.5
CN
O
O
N
Et
10e
in DMSO-d6
190
180
170
160
150
140
130
120
110
100
90
80
70
60
50
40
30
20
10
ppm

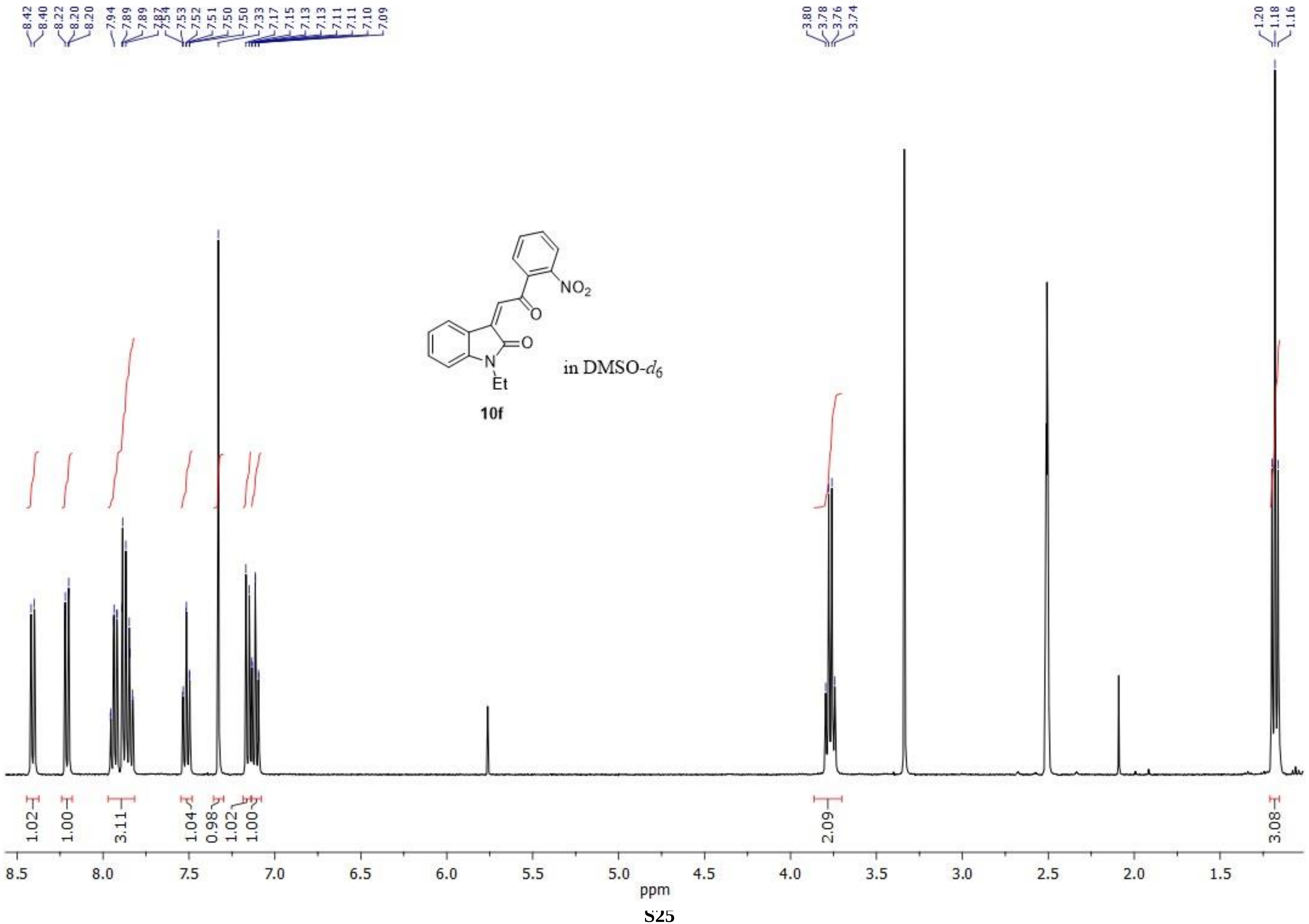

8.42
8.40
8.22
8.20
8.20
7.94
7.89
7.89
7.87
7.54
7.53
7.52
7.51
7.50
7.50
7.33
7.17
7.15
7.13
7.13
7.11
7.11
7.10
7.09
3.80
3.78
3.76
3.74
1.20
1.18
1.16
NO2
O
O
N
Et
10f
in DMSO-d6
1.02
1.00
3.11
1.04
0.98
1.02
1.00
2.09
3.08
8.5
8.0
7.5
7.0
6.5
6.0
5.5
5.0
4.5
4.0
3.5
3.0
2.5
2.0
1.5
ppm

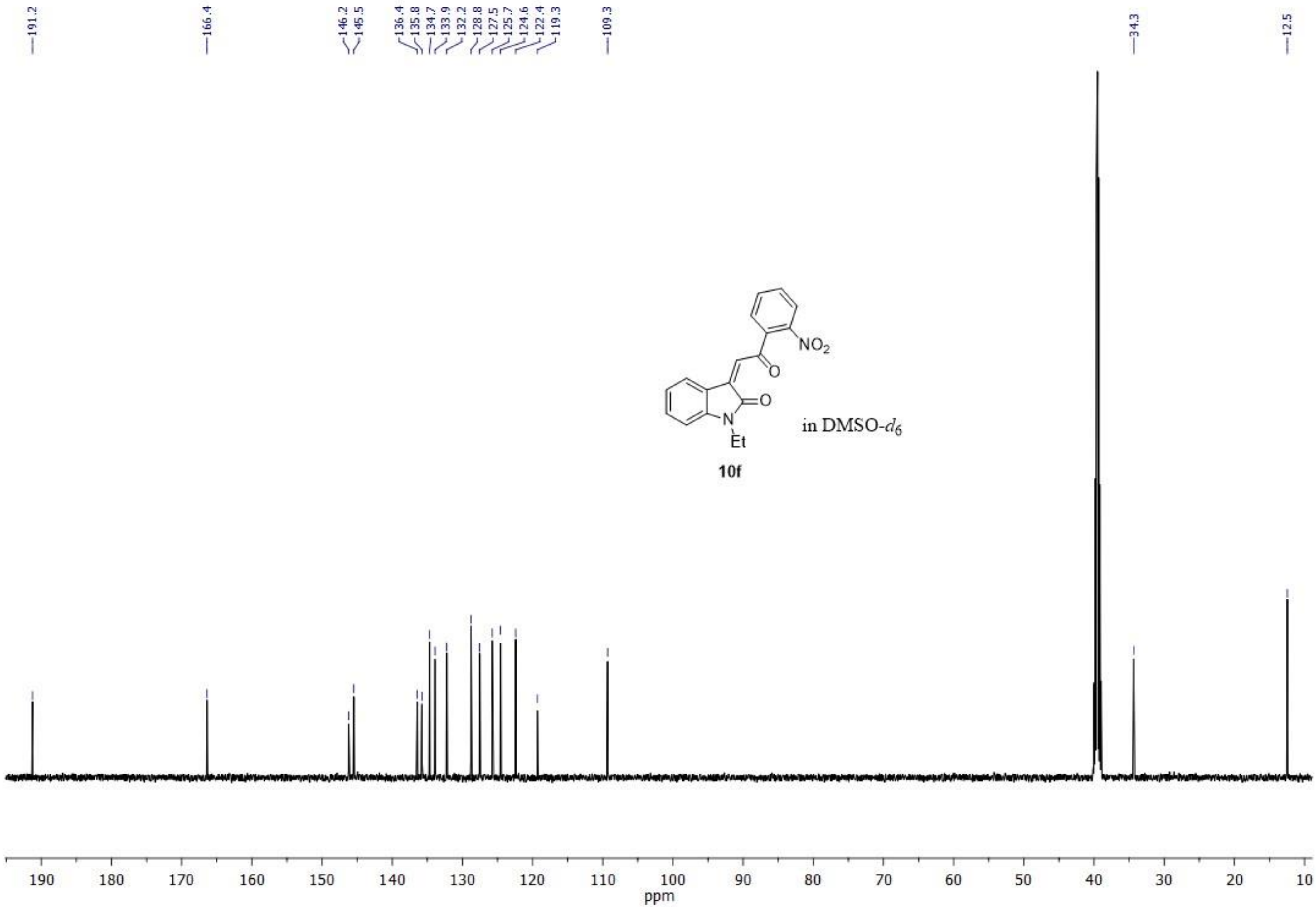
191.2
166.4
146.2
145.5
136.4
135.8
134.7
133.9
132.2
128.8
127.5
125.7
124.6
122.4
119.3
109.3
34.3
12.5
NO2
O
O
N
Et
10f
in DMSO-d6
190
180
170
160
150
140
130
120
110
100
90
80
70
60
50
40
30
20
10
ppm

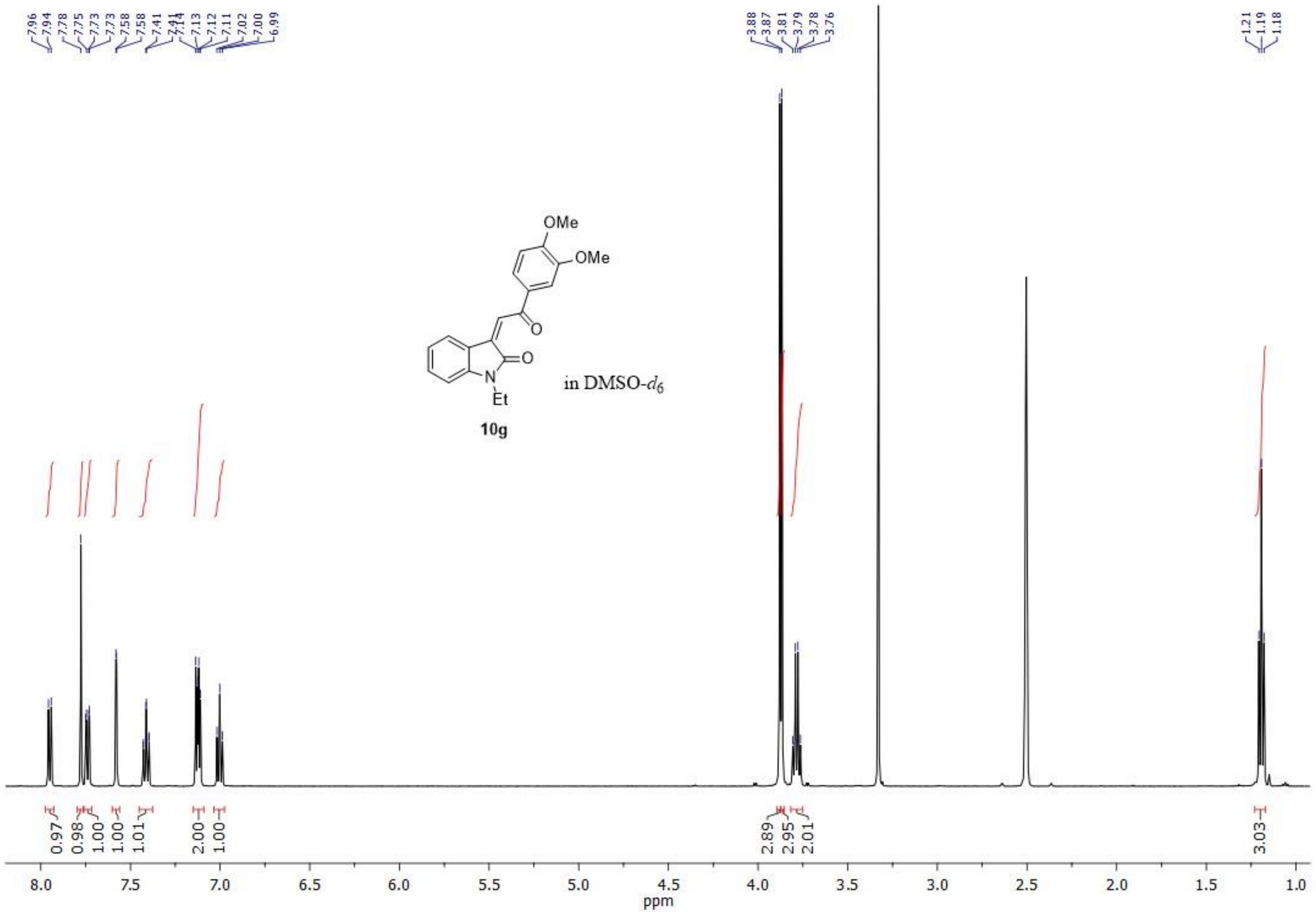

7.96
7.94
7.78
7.75
7.73
7.73
7.58
7.58
7.41
7.41
7.14
7.13
7.12
7.11
7.02
7.00
6.99
3.88
3.87
3.81
3.79
3.78
3.76
1.21
1.19
1.18
OMe
OMe
O
O
N
Et
10g
in DMSO-d_6
0.97
0.98
1.00
1.00
1.01
2.00
1.00
2.89
2.95
2.01
3.03
8.0
7.5
7.0
6.5
6.0
5.5
5.0
4.5
4.0
3.5
3.0
2.5
2.0
1.5
1.0
ppm

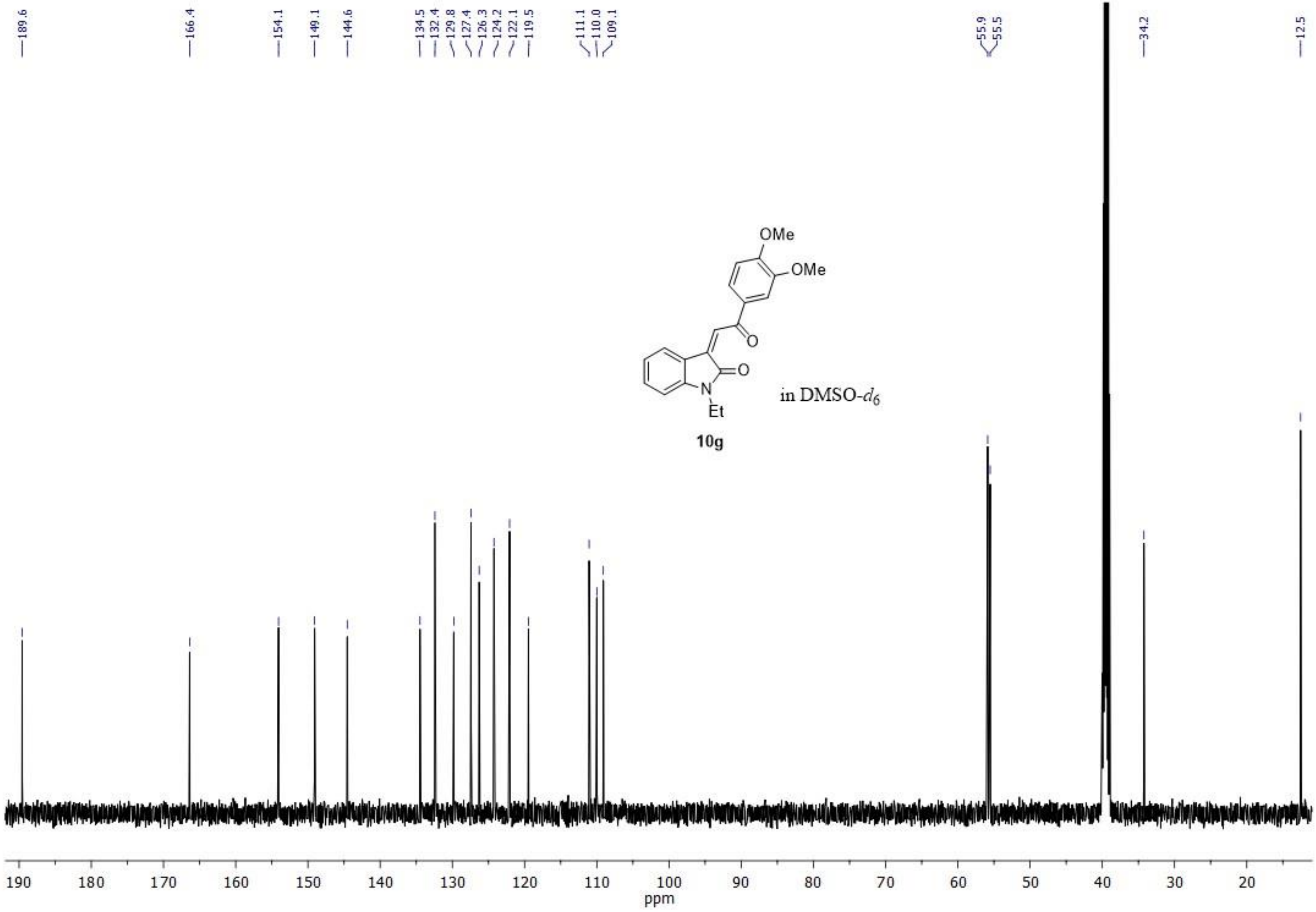
189.6
166.4
154.1
149.1
144.6
134.5
132.4
129.8
127.4
126.3
124.2
122.1
119.5
111.1
110.0
109.1
55.9
55.5
34.2
12.5
OMe
OMe
O
O
N
Et
10g
in DMSO-d_6
190 180 170 160 150 140 130 120 110 100 90 80 70 60 50 40 30 20
ppm

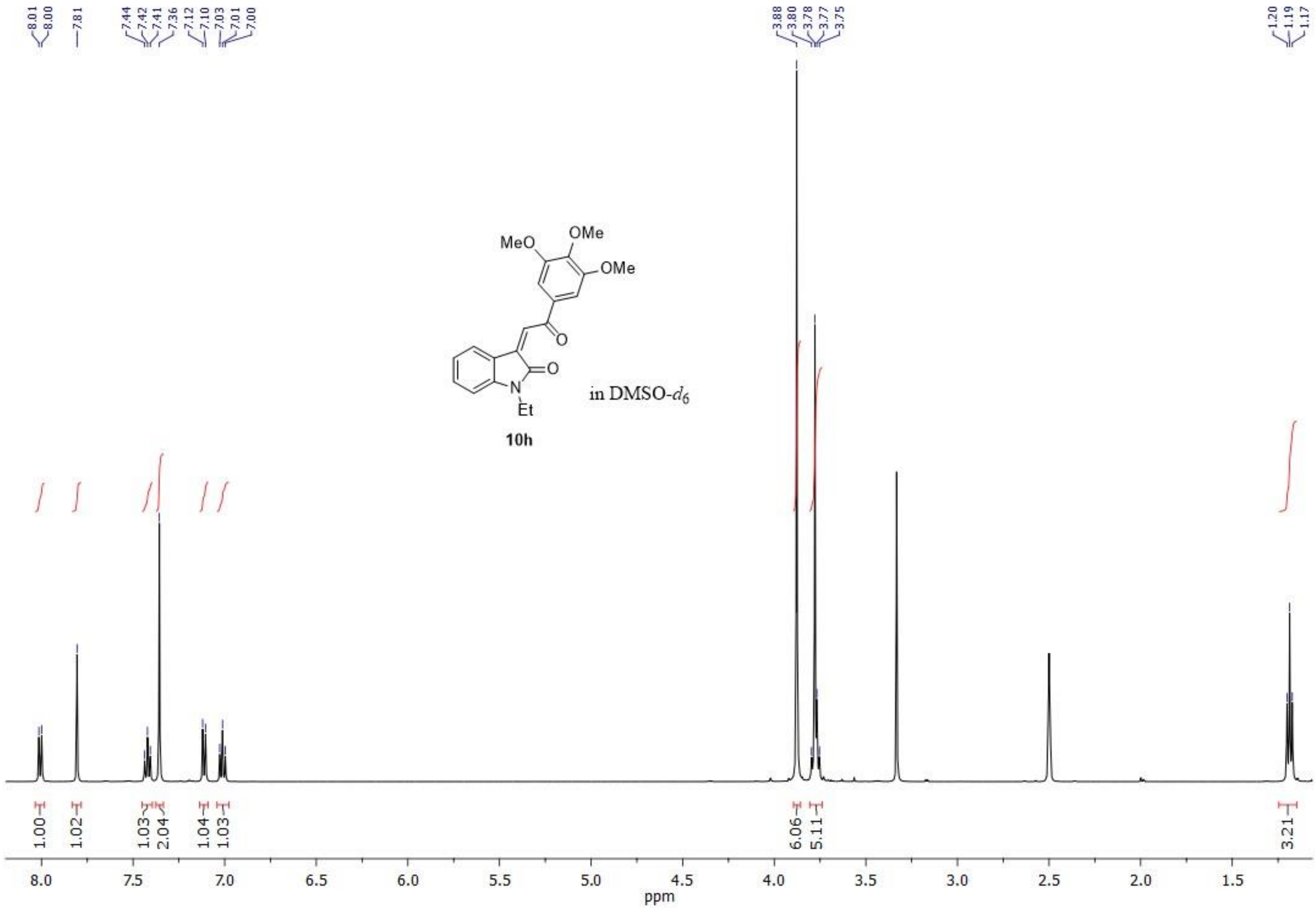
8.01
8.00
7.81
7.44
7.42
7.41
7.36
7.12
7.10
7.03
7.01
7.00
3.88
3.80
3.78
3.77
3.75
1.20
1.19
1.17
MeO
OMe
OMe
O
O
N
Et
10h
in DMSO-d_6
1.00
1.02
1.03
2.04
1.04
1.03
6.06
5.11
3.21
8.0
7.5
7.0
6.5
6.0
5.5
5.0
4.5
4.0
3.5
3.0
2.5
2.0
1.5
ppm

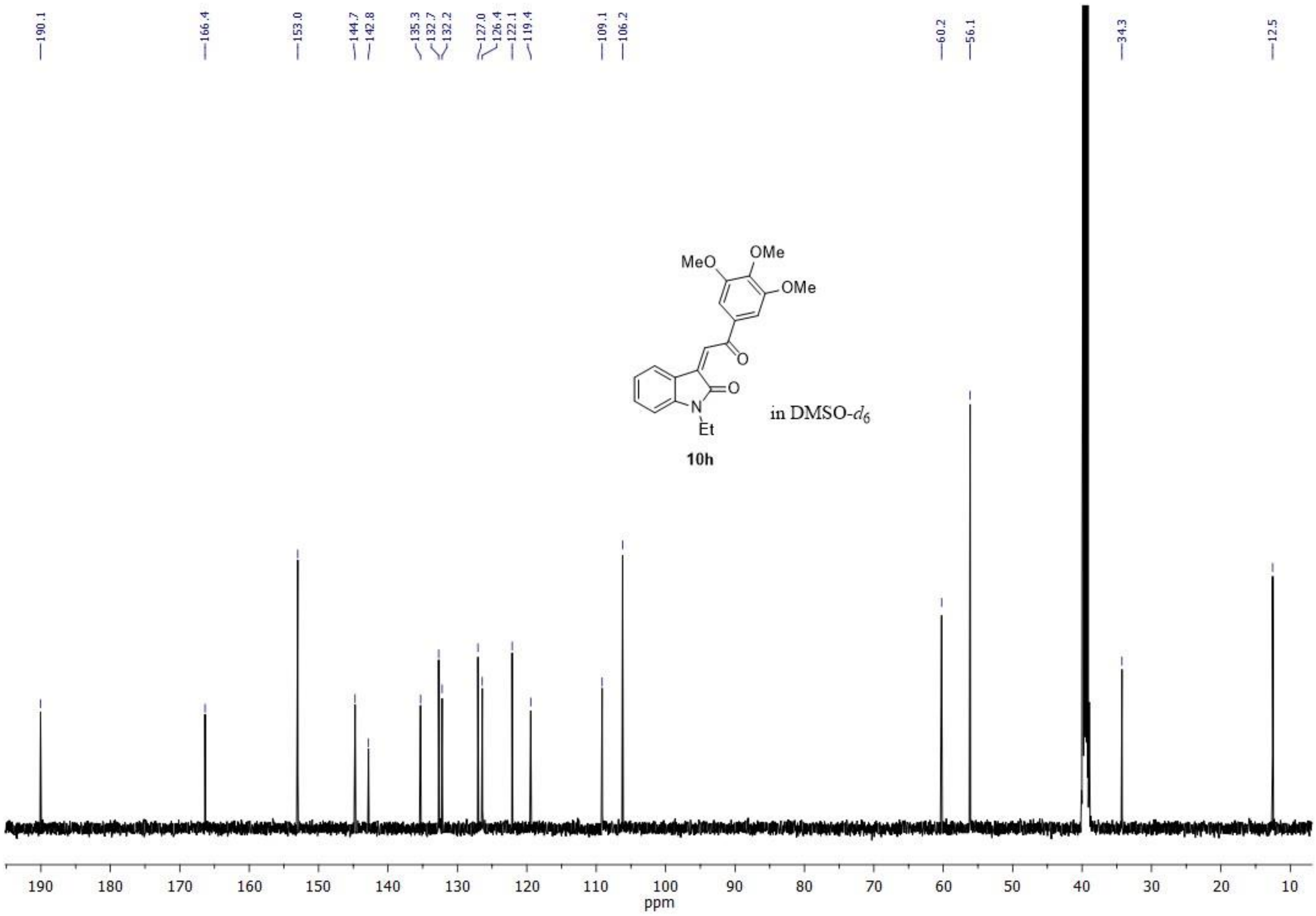
190.1
166.4
153.0
144.7
142.8
135.3
132.7
132.2
127.0
126.4
122.1
119.4
109.1
106.2
60.2
56.1
34.3
12.5
MeO
OMe
OMe
O
O
N
Et
10h
in DMSO-d_6
190
180
170
160
150
140
130
120
110
100
90
80
70
60
50
40
30
20
10
ppm

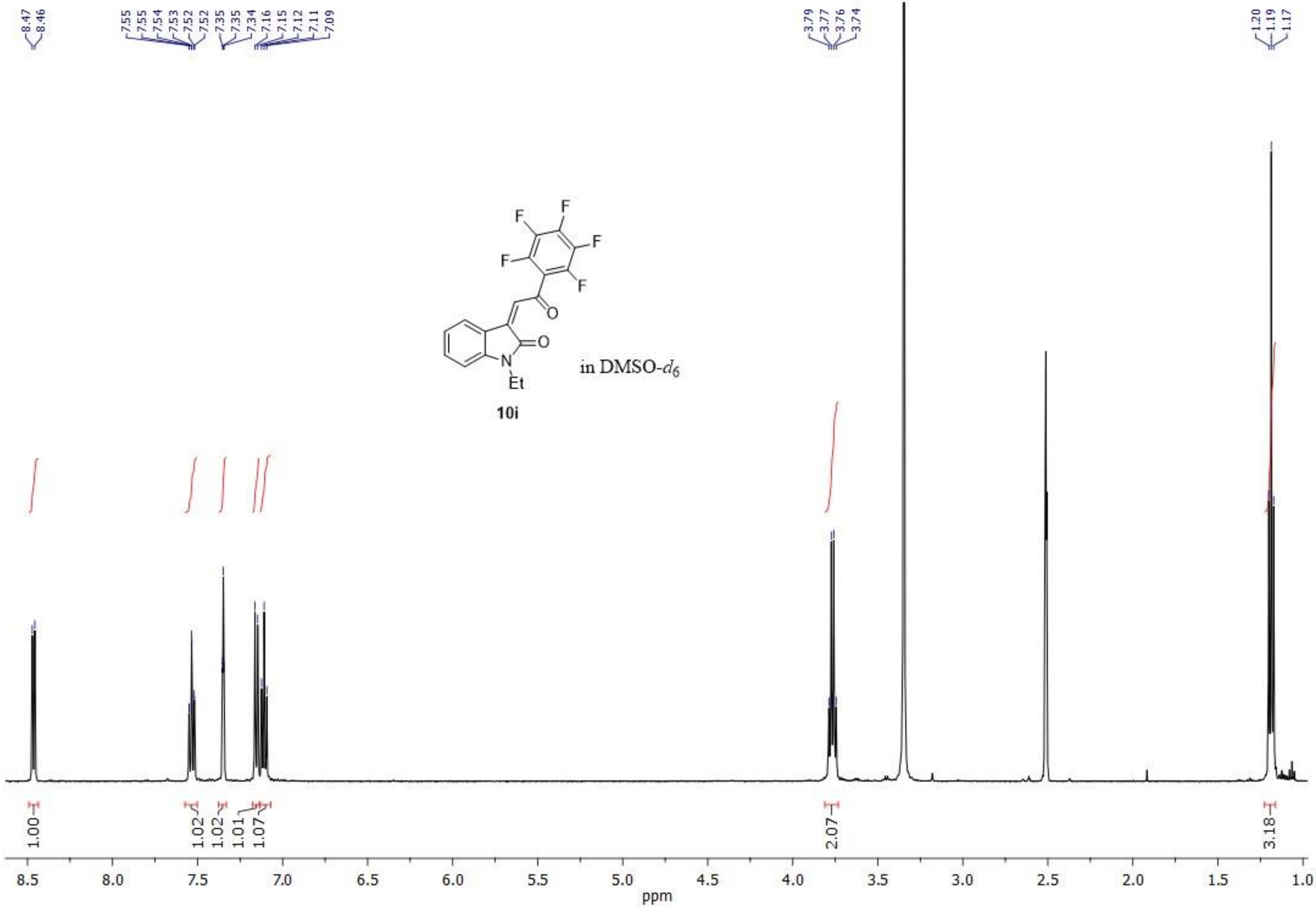
8.47
8.46
7.55
7.55
7.54
7.53
7.52
7.52
7.35
7.35
7.34
7.16
7.15
7.12
7.11
7.09
3.79
3.77
3.76
3.74
1.20
1.19
1.17
F
F
F
F
F
O
O
N
Et
10i
in DMSO-d_6
1.00
1.02
1.02
1.01
1.07
2.07
3.18
8.5
8.0
7.5
7.0
6.5
6.0
5.5
5.0
4.5
4.0
3.5
3.0
2.5
2.0
1.5
1.0
ppm

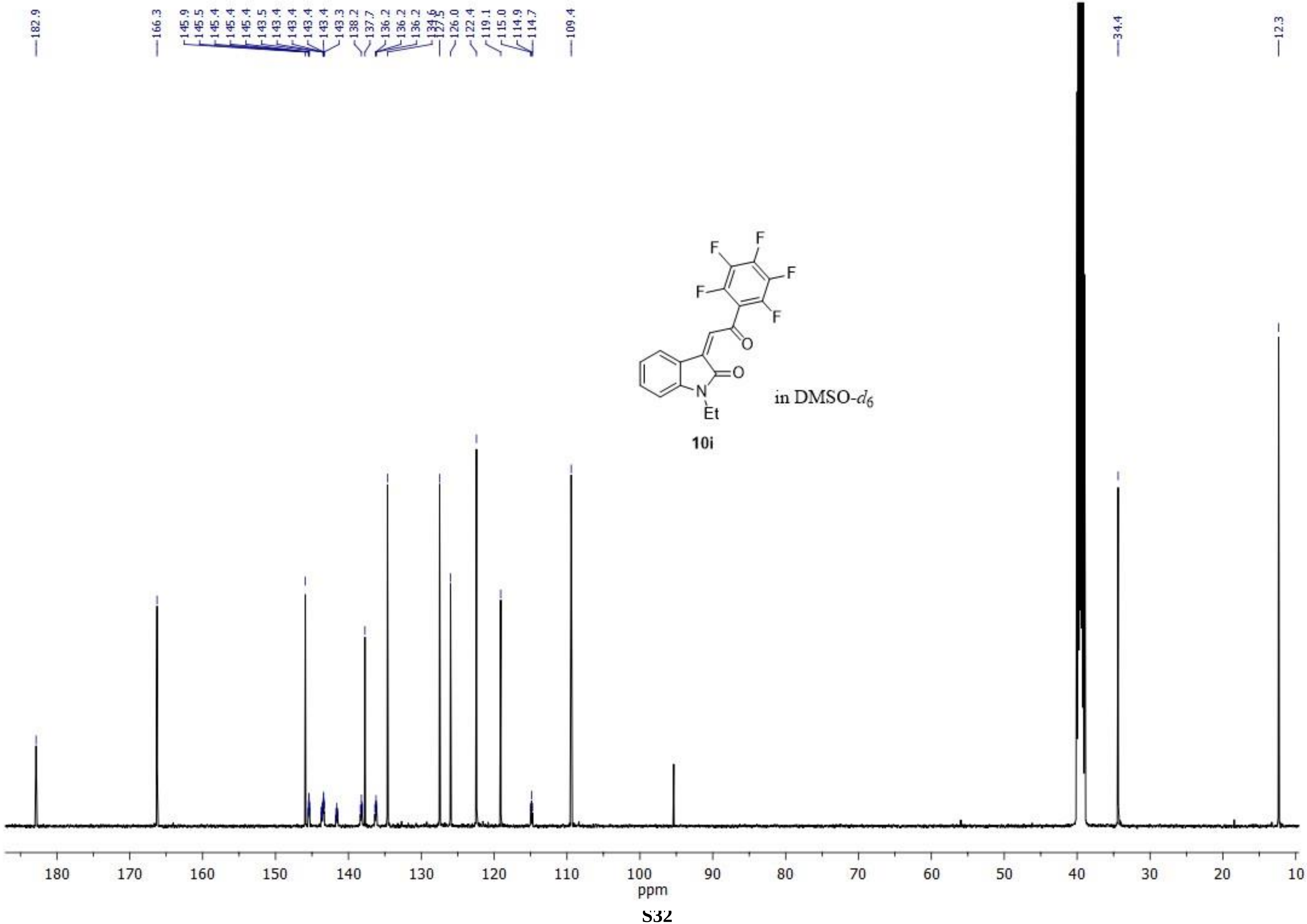
182.9
166.3
145.9
145.5
145.4
145.4
145.4
143.5
143.4
143.4
143.4
143.4
143.3
138.2
137.7
136.2
136.2
136.2
134.6
127.5
126.0
122.4
119.1
115.0
114.9
114.7
109.4
34.4
12.3
F
F
F
F
F
O
O
N
Et
10i
in DMSO-d_6
180
170
160
150
140
130
120
110
100
90
80
70
60
50
40
30
20
10
ppm

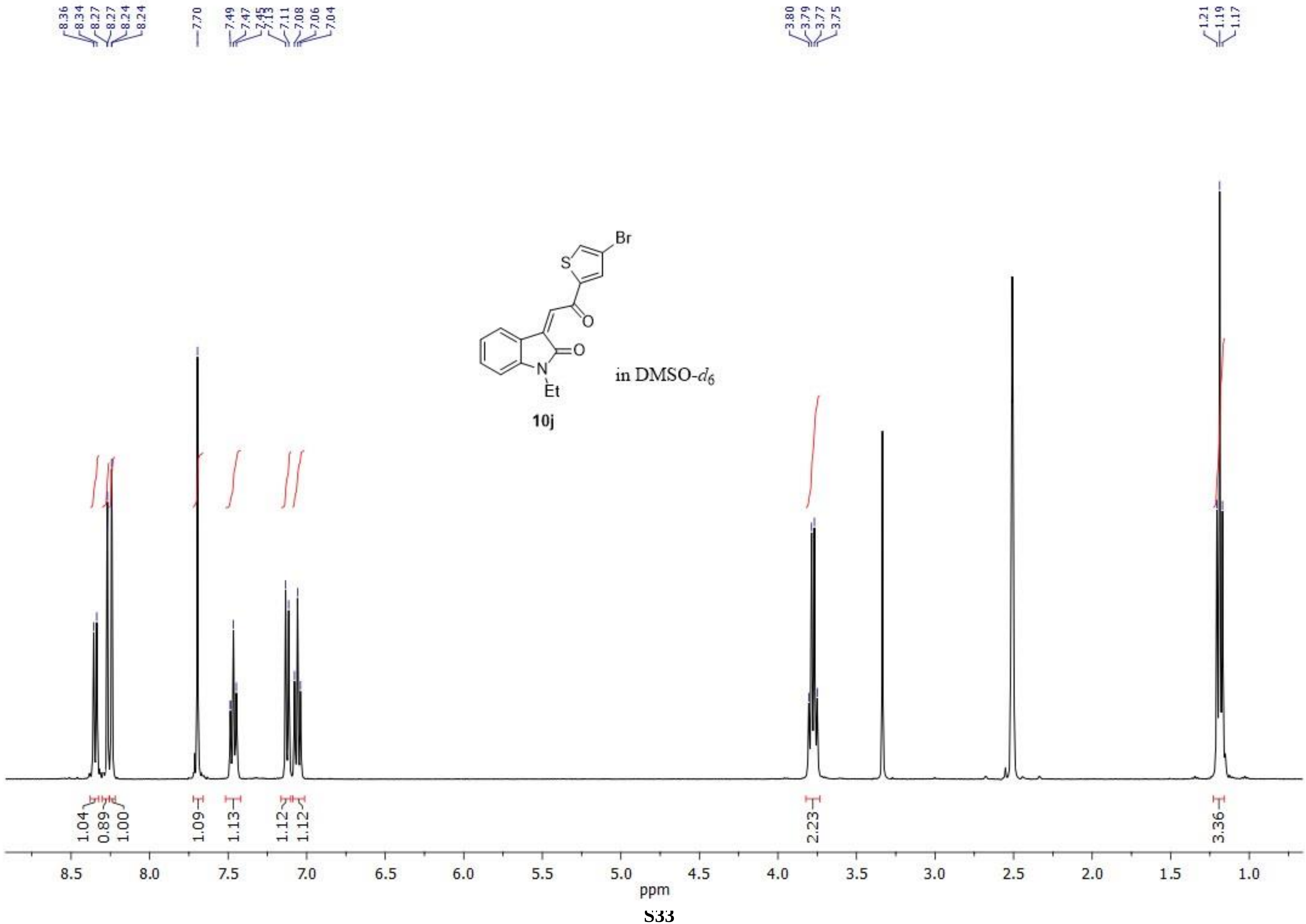

8.36 8.34 8.27 8.27 8.24 8.24
7.70
7.49 7.47 7.45 7.13 7.11 7.08 7.06 7.04
3.80 3.79 3.77 3.75
1.21 1.19 1.17
Br
S
O
O
N
Et
10j
in DMSO-d_6
1.04 0.89 1.00
1.09
1.13
1.12 1.12
2.23
3.36
8.5 8.0 7.5 7.0 6.5 6.0 5.5 5.0 4.5 4.0 3.5 3.0 2.5 2.0 1.5 1.0
ppm

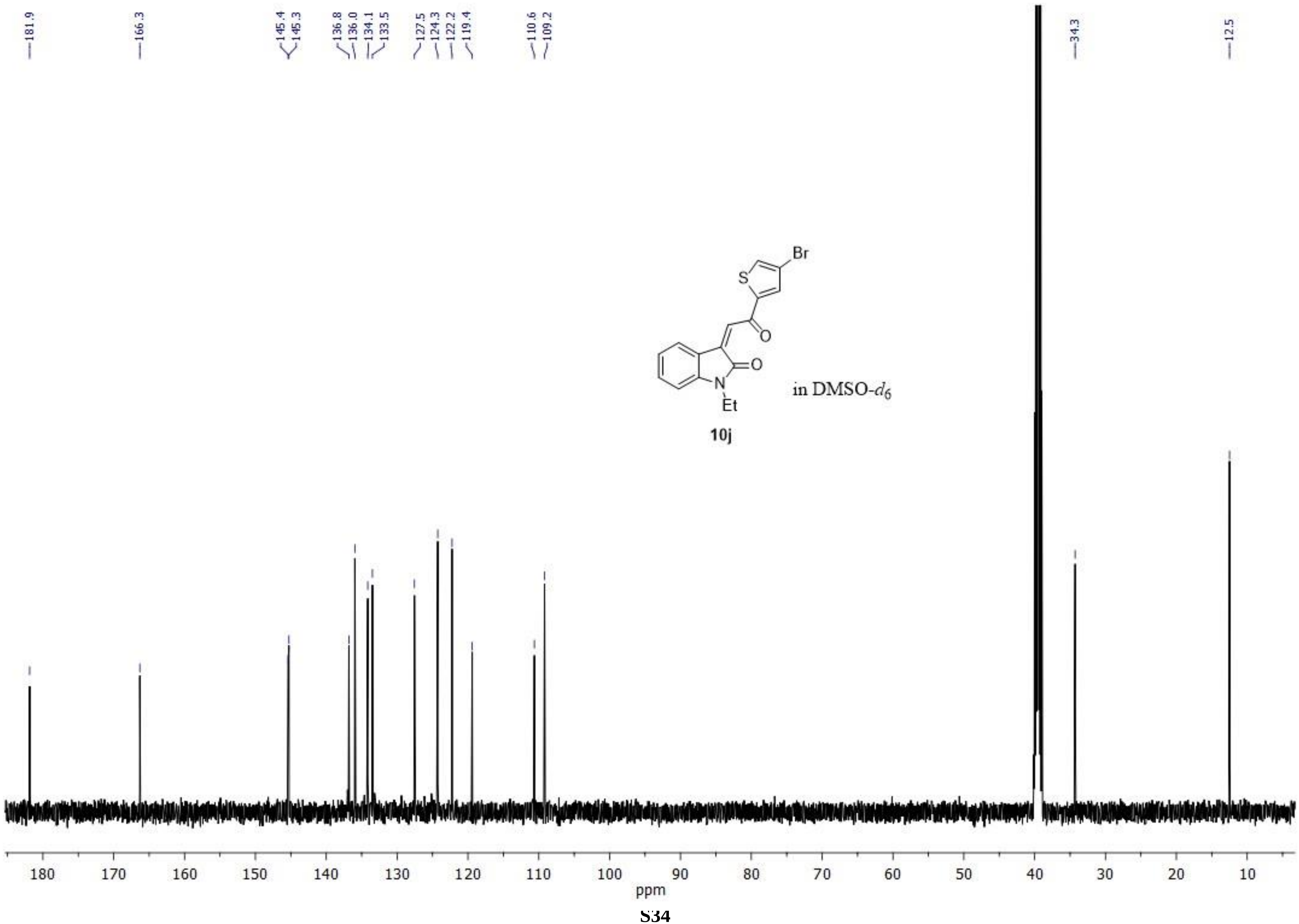
181.9
166.3
145.4
145.3
136.8
136.0
134.1
133.5
127.5
124.3
122.2
119.4
110.6
109.2
34.3
12.5
Br
S
O
O
N
Et
10j
in DMSO-d_6
180 170 160 150 140 130 120 110 100 90 80 70 60 50 40 30 20 10
ppm

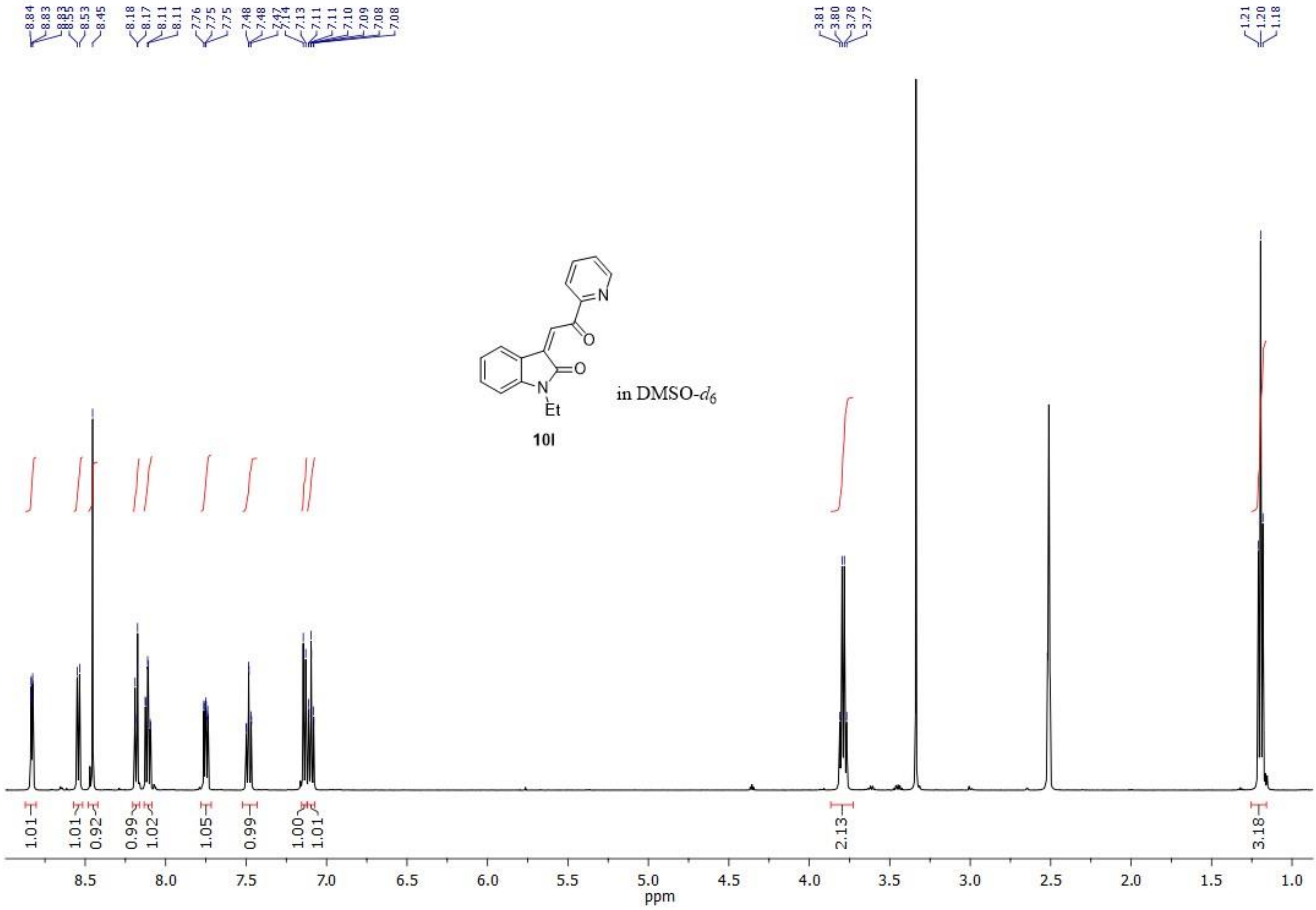

8.84
8.83
8.83
8.55
8.53
8.45
8.18
8.17
8.11
8.11
7.76
7.75
7.75
7.48
7.48
7.47
7.14
7.13
7.11
7.11
7.10
7.09
7.08
7.08
3.81
3.80
3.78
3.77
1.21
1.20
1.18
N
O
O
N
Et
10l
in DMSO-d_6
1.01
1.01
0.92
0.99
1.02
1.05
0.99
1.00
1.01
2.13
3.18
8.5
8.0
7.5
7.0
6.5
6.0
5.5
5.0
4.5
4.0
3.5
3.0
2.5
2.0
1.5
1.0
ppm

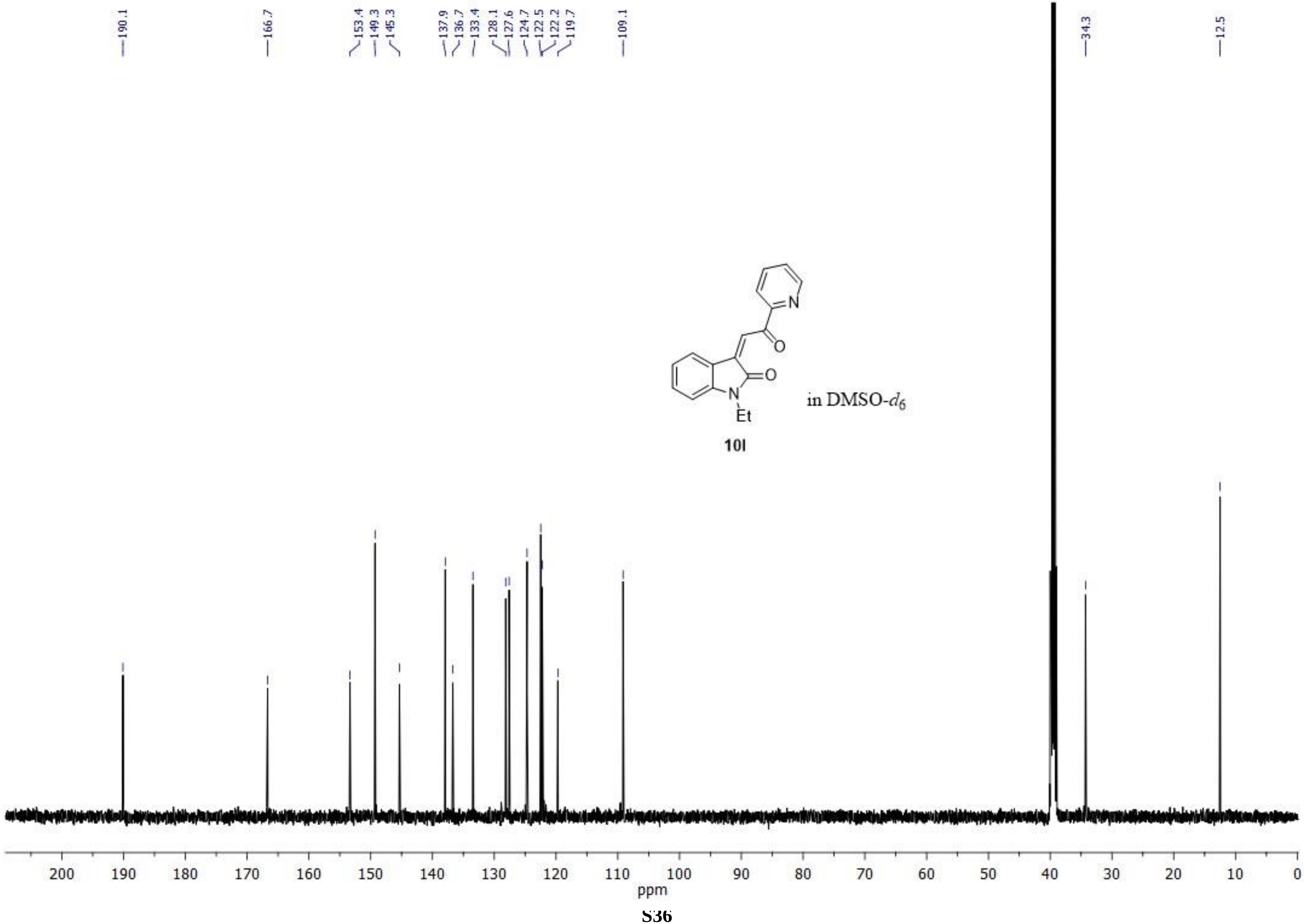
190.1
166.7
153.4
149.3
145.3
137.9
136.7
133.4
128.1
127.6
124.7
122.5
122.2
119.7
109.1
34.3
12.5
N
O
O
N
Et
10l
in DMSO-d_6
200
190
180
170
160
150
140
130
120
110
100
90
80
70
60
50
40
30
20
10
0
ppm

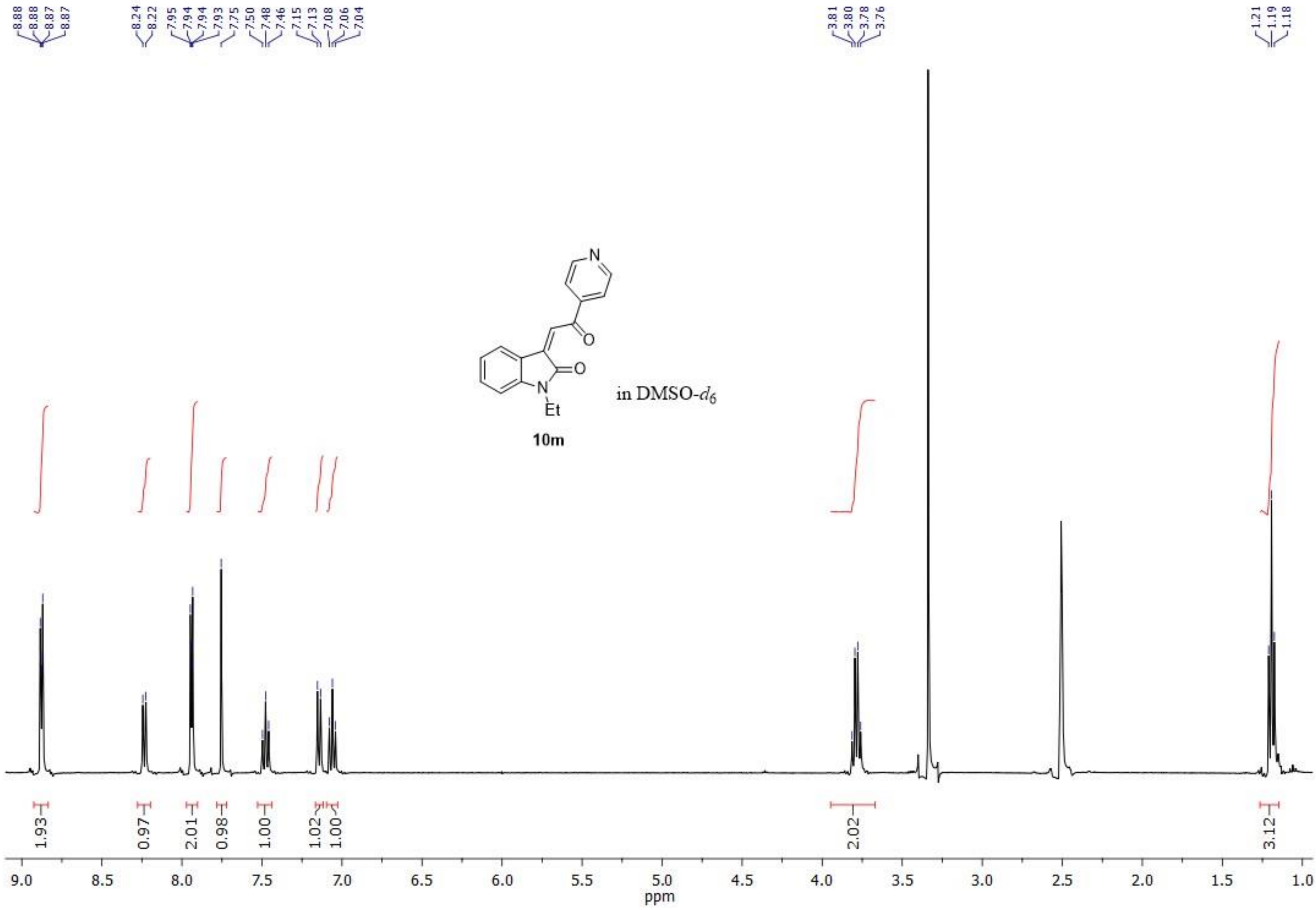
8.88
8.88
8.87
8.87
8.24
8.22
7.95
7.94
7.94
7.93
7.75
7.50
7.48
7.46
7.15
7.13
7.08
7.06
7.04
3.81
3.80
3.78
3.76
1.21
1.19
1.18
N
O
O
N
Et
10m
in DMSO-d_6
1.93
0.97
2.01
0.98
1.00
1.02
1.00
2.02
3.12
9.0
8.5
8.0
7.5
7.0
6.5
6.0
5.5
5.0
4.5
4.0
3.5
3.0
2.5
2.0
1.5
1.0
ppm

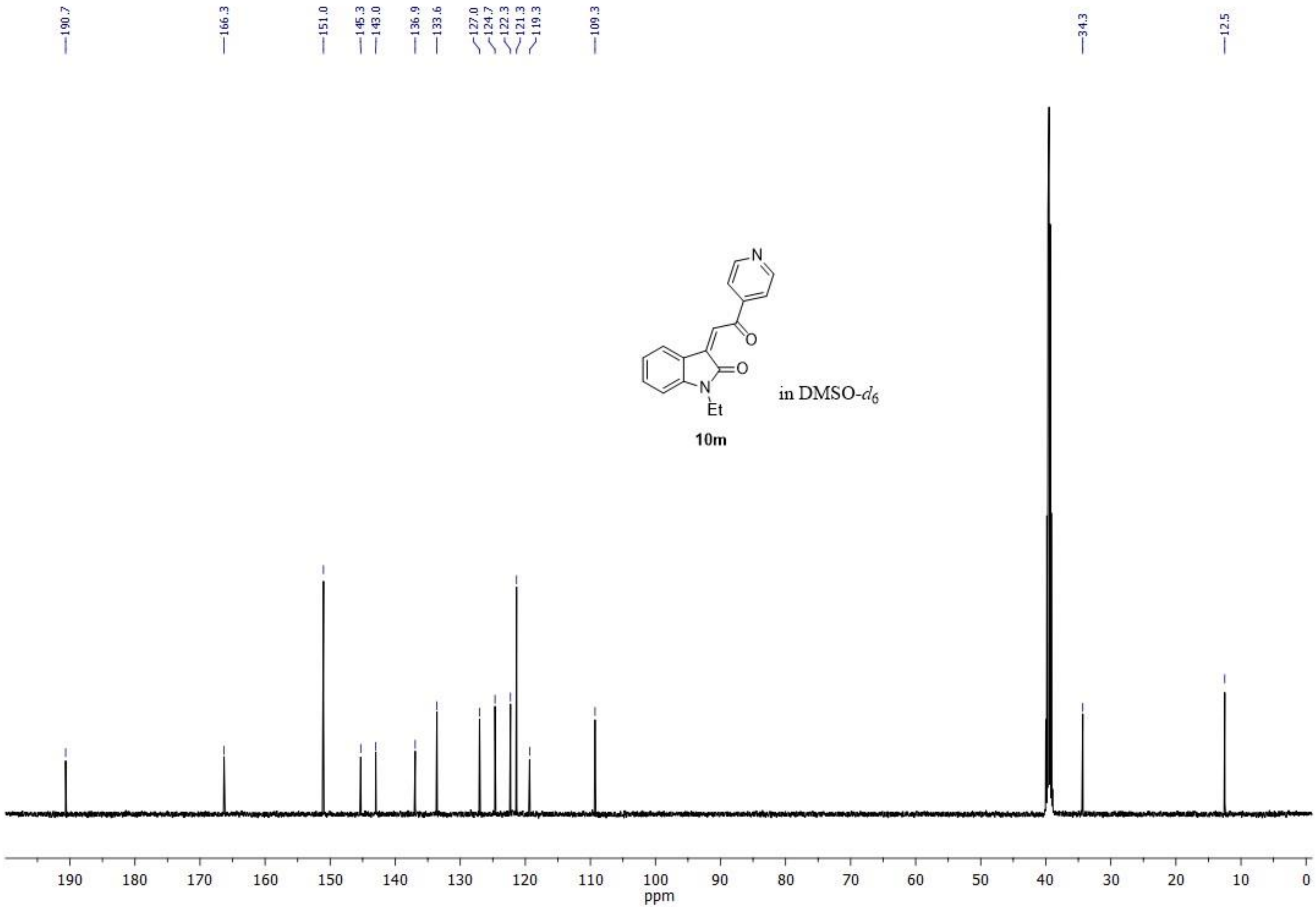

190.7
166.3
151.0
145.3
143.0
136.9
133.6
127.0
124.7
122.3
121.3
119.3
109.3
34.3
12.5
N
O
O
N
Et
10m
in DMSO-d_6
190 180 170 160 150 140 130 120 110 100 90 80 70 60 50 40 30 20 10 0
ppm

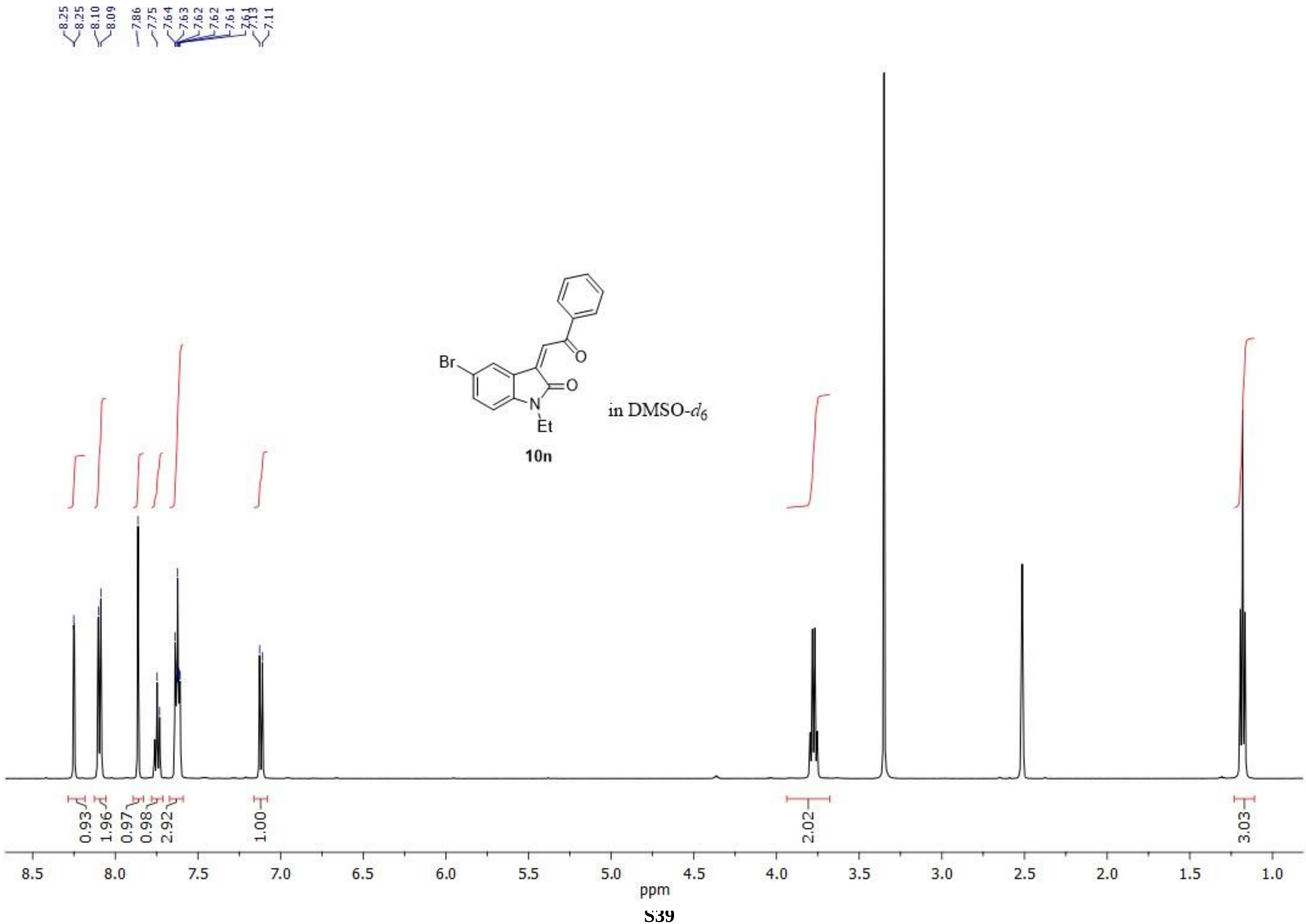

8.25
8.25
8.10
8.09
7.86
7.75
7.64
7.63
7.62
7.62
7.61
7.61
7.13
7.11
Br
O
O
N
Et
10n
in DMSO-d_6
0.93
1.96
0.97
0.98
2.92
1.00
2.02
3.03
8.5
8.0
7.5
7.0
6.5
6.0
5.5
5.0
4.5
4.0
3.5
3.0
2.5
2.0
1.5
1.0
ppm

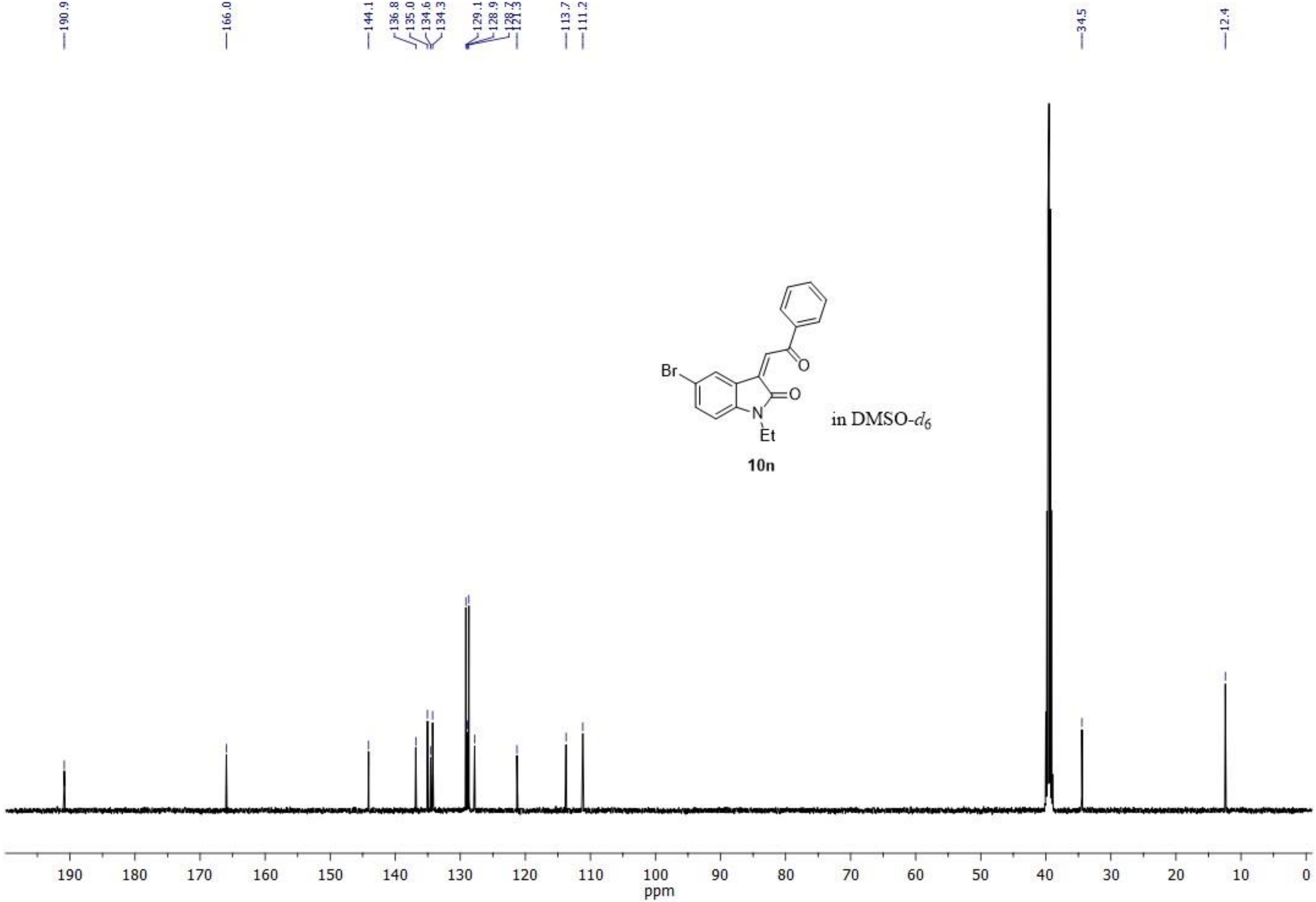
190.9
166.0
144.1
136.8
135.0
134.6
134.3
129.1
128.9
128.7
121.3
113.7
111.2
34.5
12.4
Br
O
O
N
Et
10n
in DMSO-d_6
190 180 170 160 150 140 130 120 110 100 90 80 70 60 50 40 30 20 10 0
ppm

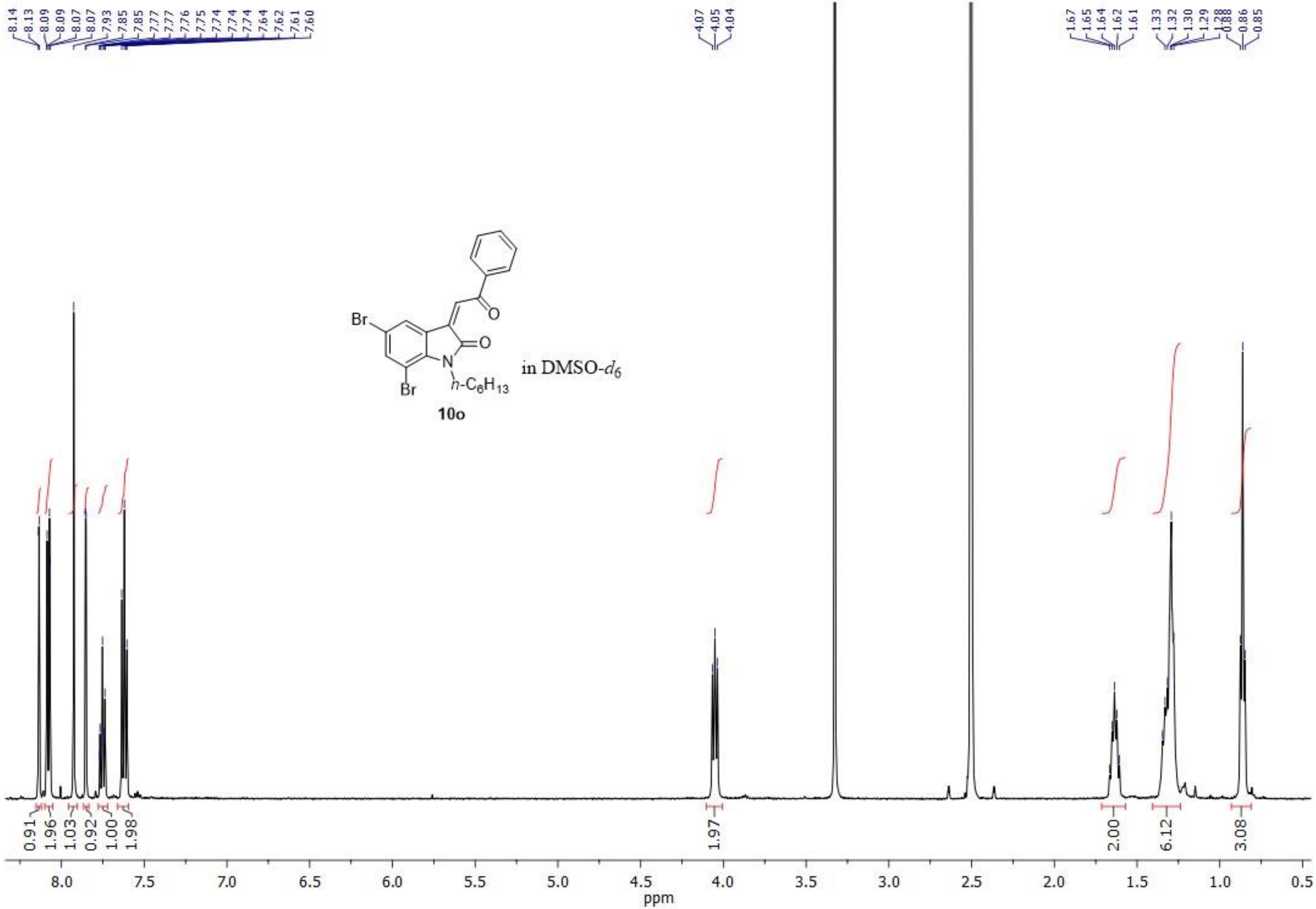
8.14
8.13
8.09
8.09
8.07
8.07
7.93
7.85
7.85
7.77
7.77
7.76
7.75
7.74
7.74
7.74
7.64
7.62
7.61
7.60
4.07
4.05
4.04
1.67
1.65
1.64
1.62
1.61
1.33
1.32
1.30
1.29
1.28
0.88
0.86
0.85
Br
Br
O
O
N
n-C6H13
10o
in DMSO-d6
0.91
1.96
1.03
0.92
1.00
1.98
1.97
2.00
6.12
3.08
8.0
7.5
7.0
6.5
6.0
5.5
5.0
4.5
4.0
3.5
3.0
2.5
2.0
1.5
1.0
0.5
ppm

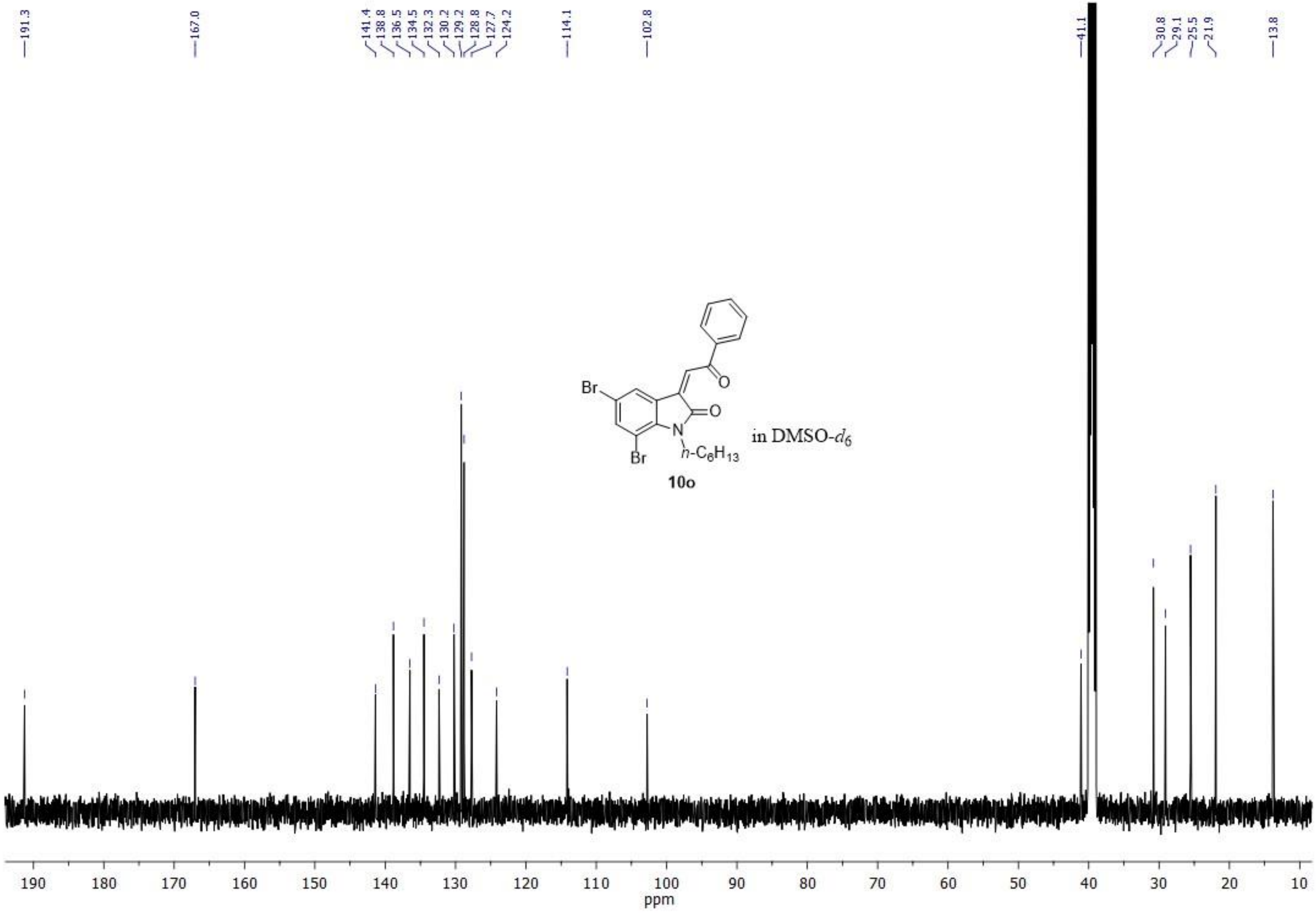
191.3
167.0
141.4
138.8
136.5
134.5
132.3
130.2
129.2
128.8
127.7
124.2
114.1
102.8
41.1
30.8
29.1
25.5
21.9
13.8
Br
Br
O
O
N
n-C6H13
10o
in DMSO-d6
190 180 170 160 150 140 130 120 110 100 90 80 70 60 50 40 30 20 10
ppm

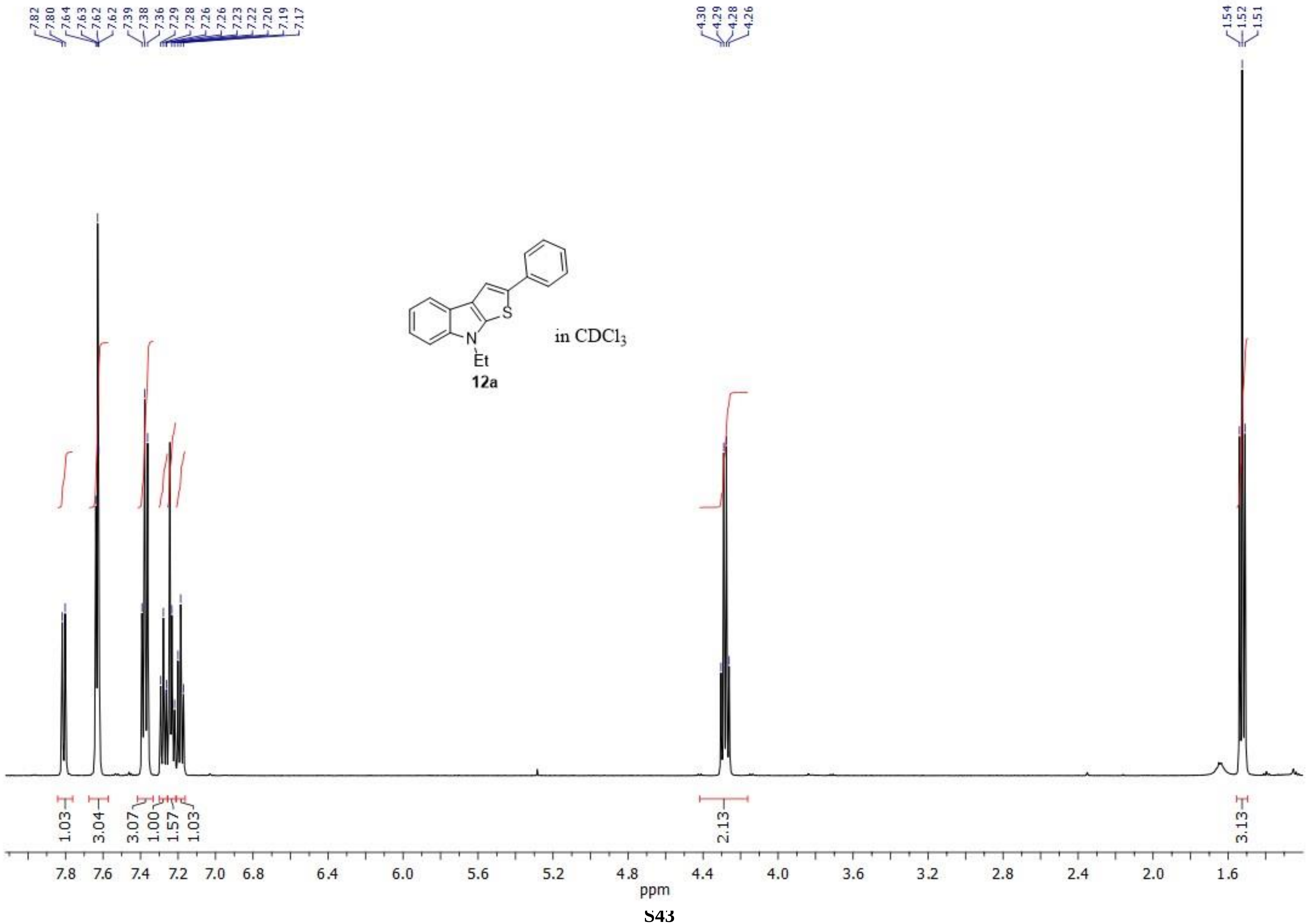
7.82
7.80
7.64
7.63
7.62
7.62
7.39
7.38
7.36
7.29
7.28
7.26
7.26
7.23
7.22
7.20
7.19
7.17
4.30
4.29
4.28
4.26
1.54
1.52
1.51
S
N
Et
12a
in CDCl3
1.03
3.04
3.07
1.00
1.57
1.03
2.13
3.13
7.8 7.6 7.4 7.2 7.0 6.8 6.4 6.0 5.6 5.2 4.8 4.4 4.0 3.6 3.2 2.8 2.4 2.0 1.6
ppm

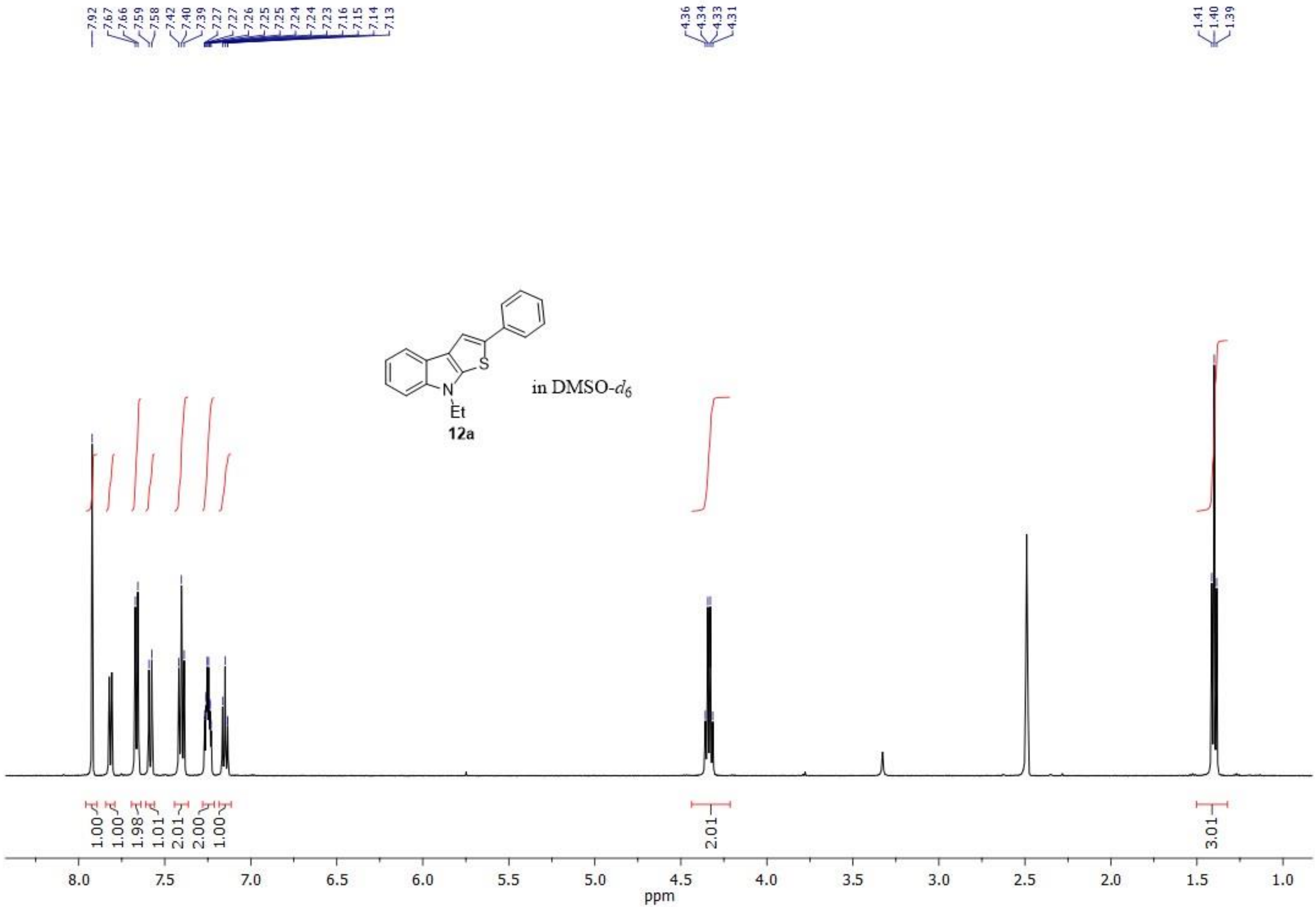
7.92
7.67
7.66
7.59
7.58
7.42
7.40
7.39
7.27
7.27
7.26
7.25
7.25
7.24
7.24
7.23
7.16
7.15
7.14
7.13
4.36
4.34
4.33
4.31
1.41
1.40
1.39
S
N
Et
12a
in DMSO-d_6
1.00
1.00
1.98
1.01
2.01
2.00
1.00
2.01
3.01
8.0
7.5
7.0
6.5
6.0
5.5
5.0
4.5
4.0
3.5
3.0
2.5
2.0
1.5
1.0
ppm

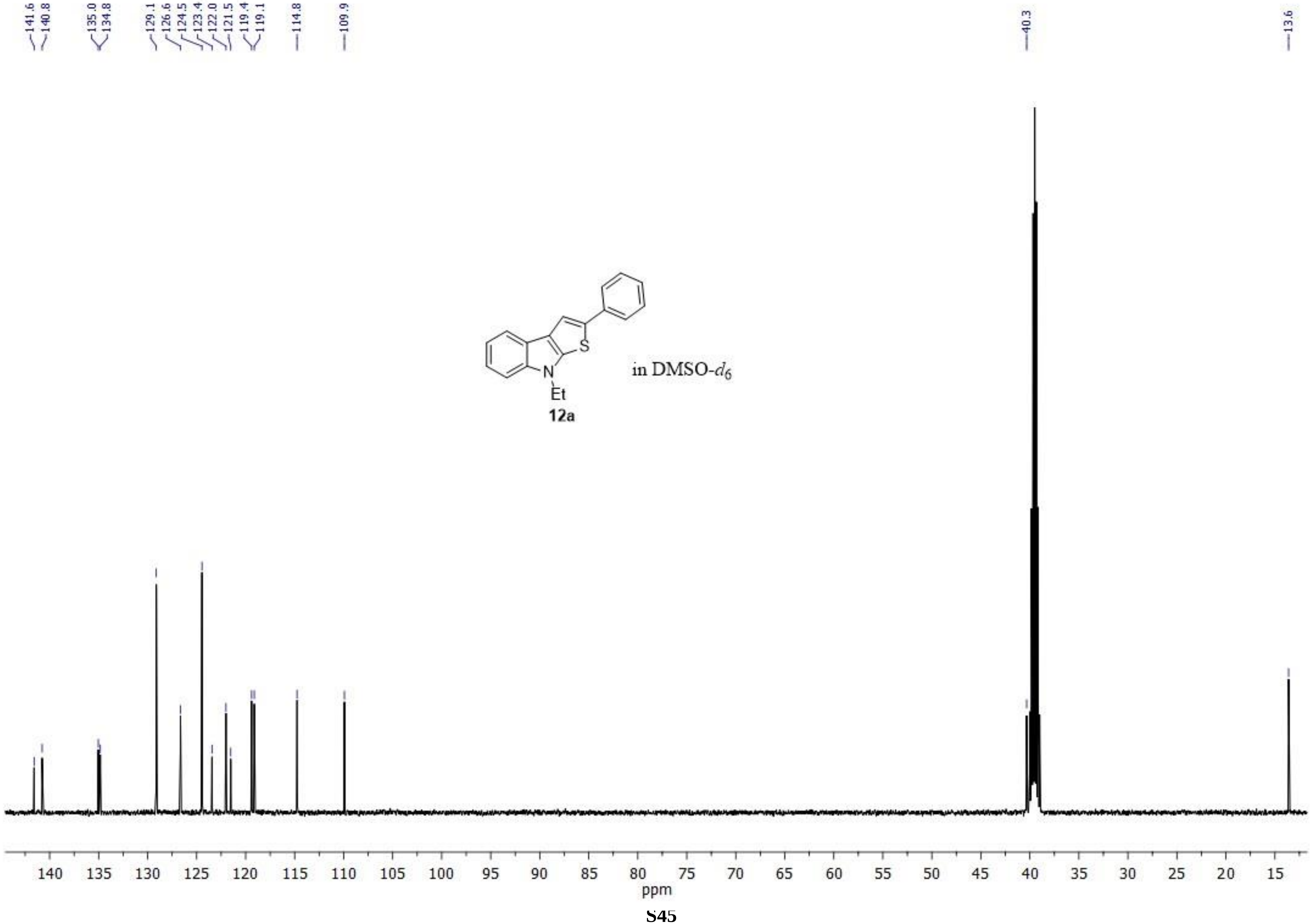
141.6
140.8
135.0
134.8
129.1
126.6
124.5
123.4
122.0
121.5
119.4
119.1
114.8
109.9
40.3
13.6
S
N
Et
12a
in DMSO-d_6
140 135 130 125 120 115 110 105 100 95 90 85 80 75 70 65 60 55 50 45 40 35 30 25 20 15
ppm

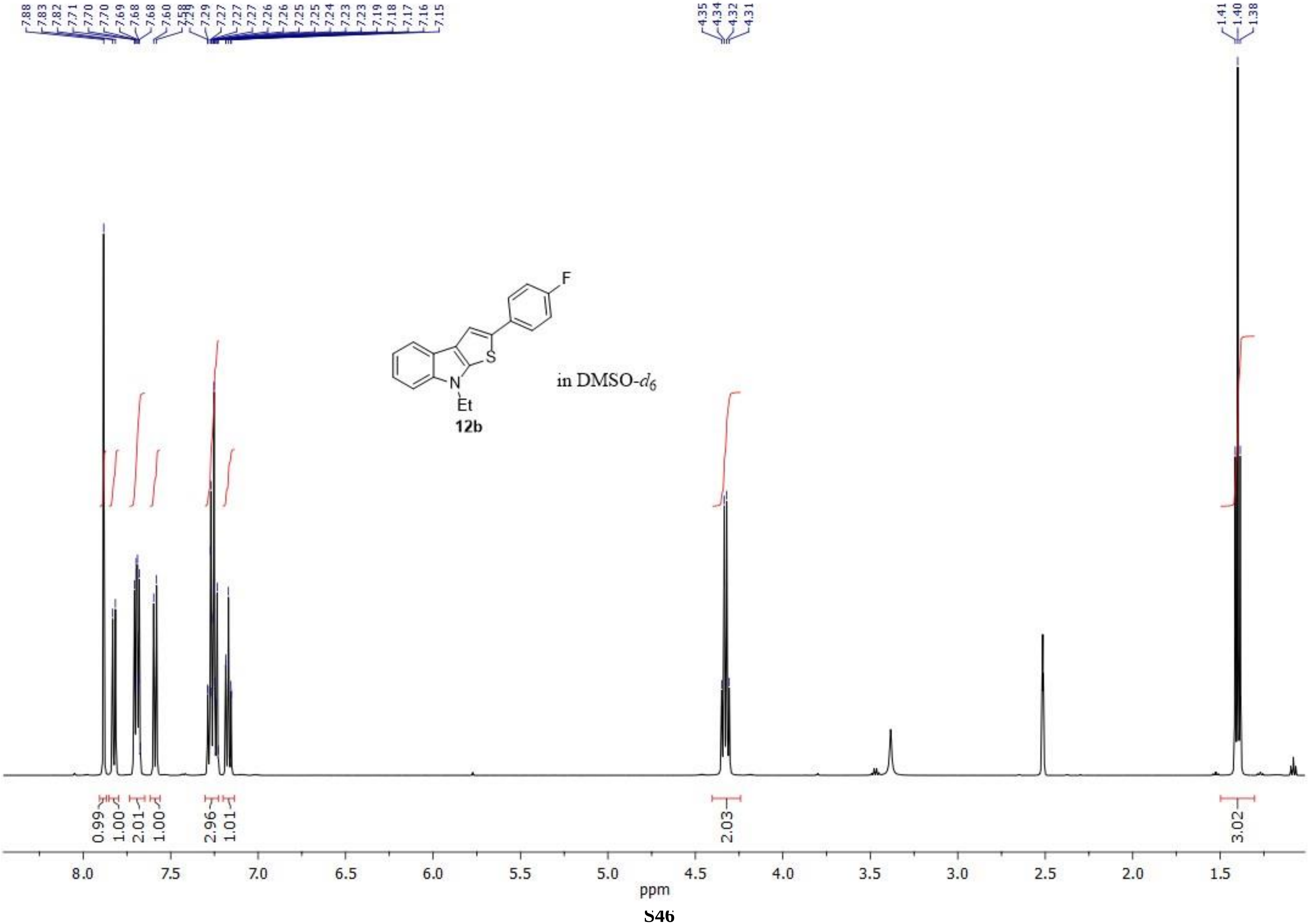
7.88
7.83
7.82
7.71
7.70
7.70
7.69
7.68
7.68
7.60
7.58
7.29
7.27
7.27
7.27
7.26
7.26
7.25
7.25
7.24
7.23
7.23
7.19
7.18
7.17
7.16
7.15
4.35
4.34
4.32
4.31
1.41
1.40
1.38
F
S
N
Et
12b
in DMSO-d_6
0.99
1.00
2.01
1.00
2.96
1.01
2.03
3.02
8.0
7.5
7.0
6.5
6.0
5.5
5.0
4.5
4.0
3.5
3.0
2.5
2.0
1.5
ppm

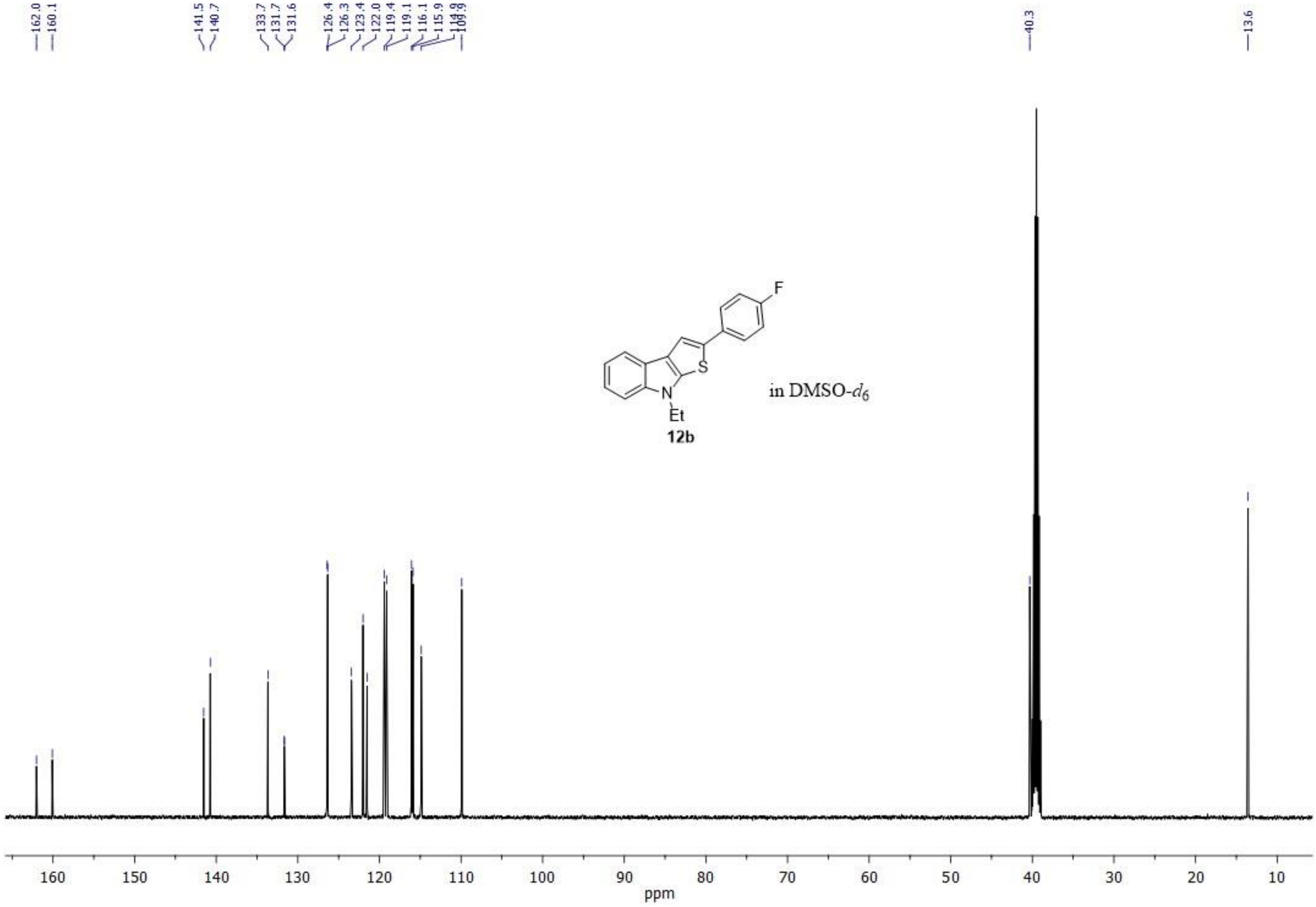

162.0
160.1
141.5
140.7
133.7
131.7
131.6
126.4
126.3
123.4
122.0
119.4
119.1
116.1
115.9
114.9
109.9
40.3
13.6
F
S
N
Et
12b
in DMSO-d_6
160
150
140
130
120
110
100
90
80
70
60
50
40
30
20
10
ppm

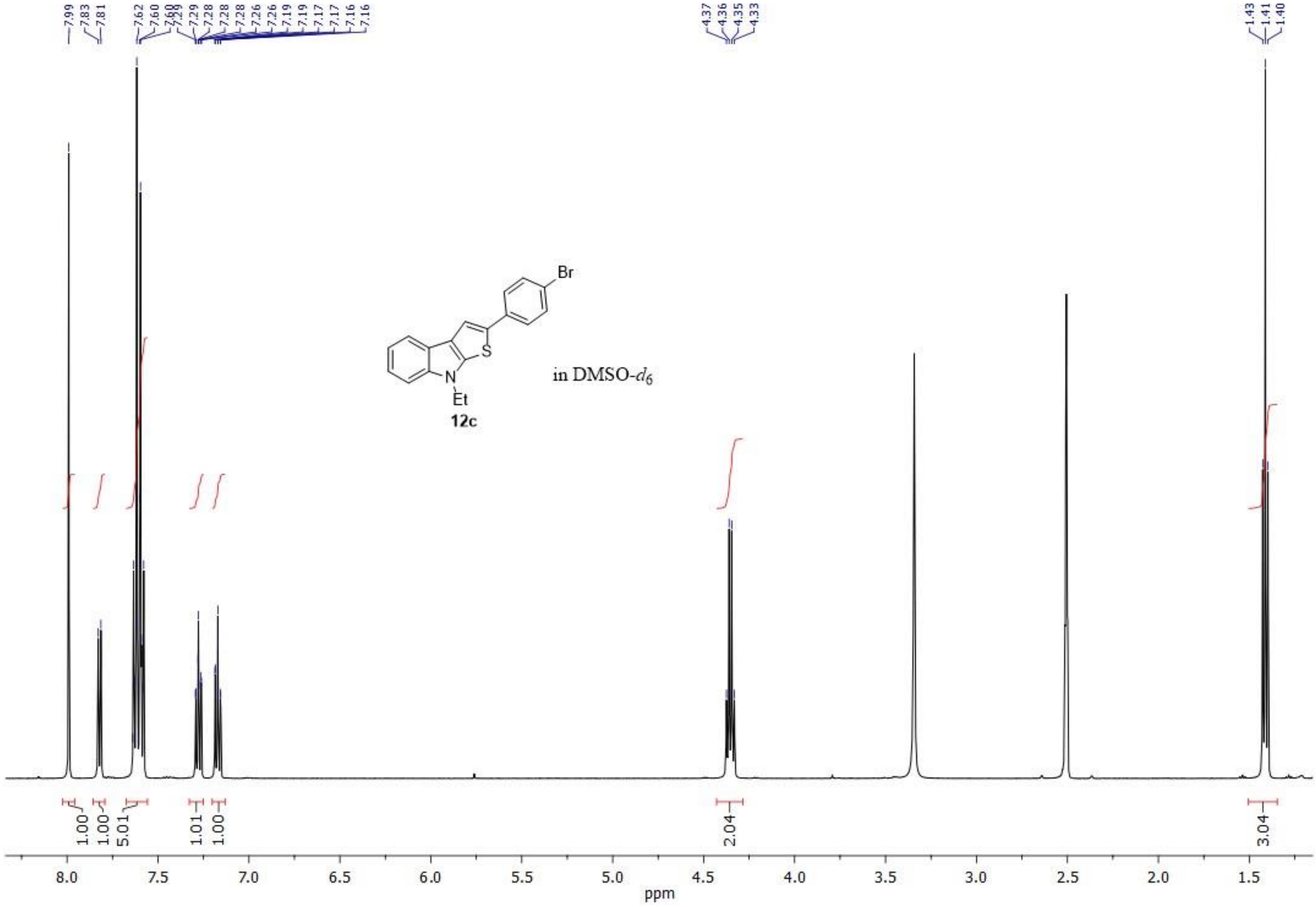
7.99
7.83
7.81
7.62
7.60
7.60
7.59
7.29
7.28
7.28
7.28
7.26
7.26
7.19
7.19
7.17
7.17
7.16
7.16
4.37
4.36
4.35
4.33
1.43
1.41
1.40
Br
S
N
Et
12c
in DMSO-d6
1.00
1.00
5.01
1.01
1.00
2.04
3.04
8.0
7.5
7.0
6.5
6.0
5.5
5.0
4.5
4.0
3.5
3.0
2.5
2.0
1.5
ppm

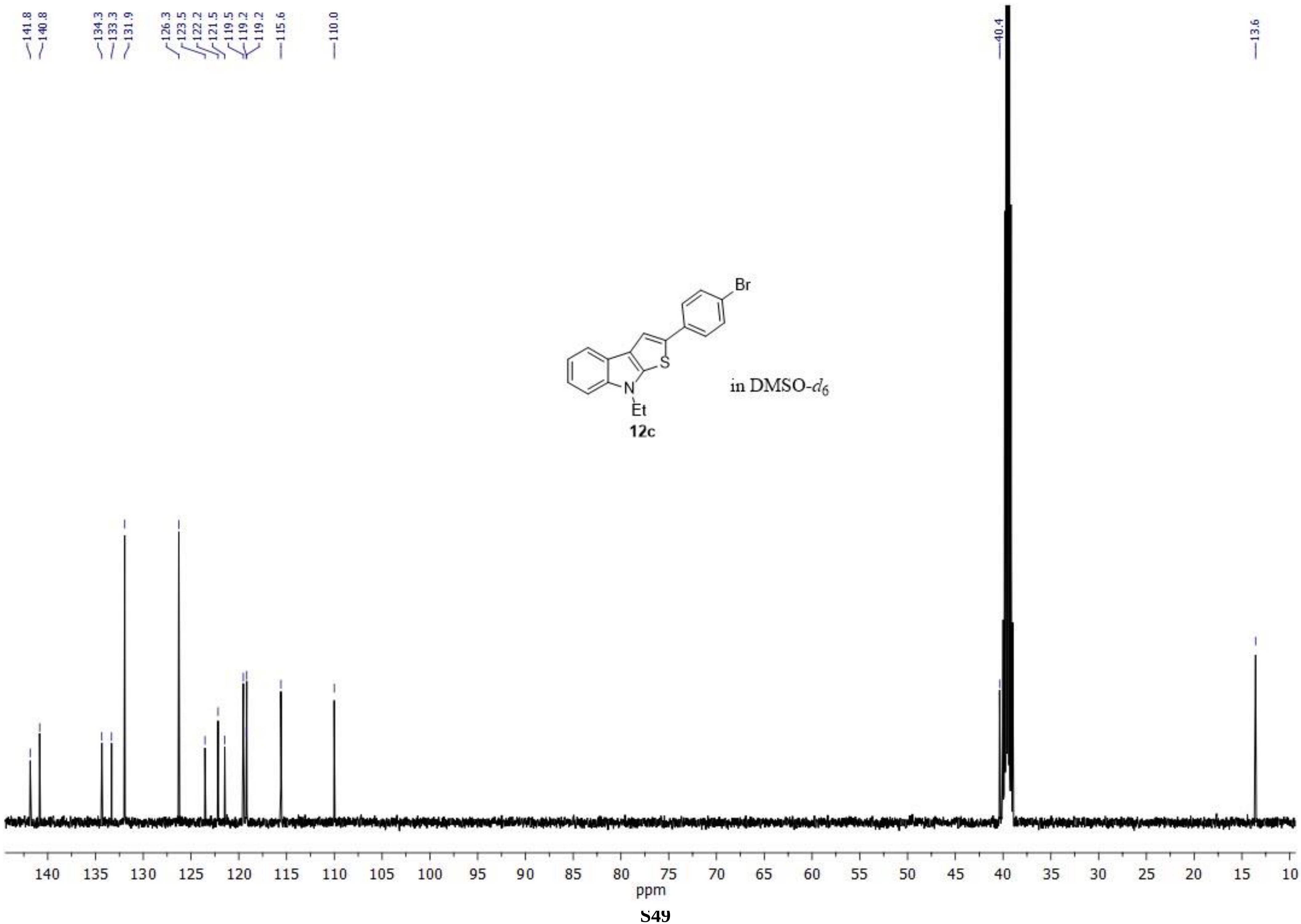

141.8
140.8
134.3
133.3
131.9
126.3
123.5
122.2
121.5
119.5
119.2
119.2
115.6
110.0
40.4
13.6
Br
S
N
Et
in DMSO-d_6
140 135 130 125 120 115 110 105 100 95 90 85 80 75 70 65 60 55 50 45 40 35 30 25 20 15 10
ppm

12c

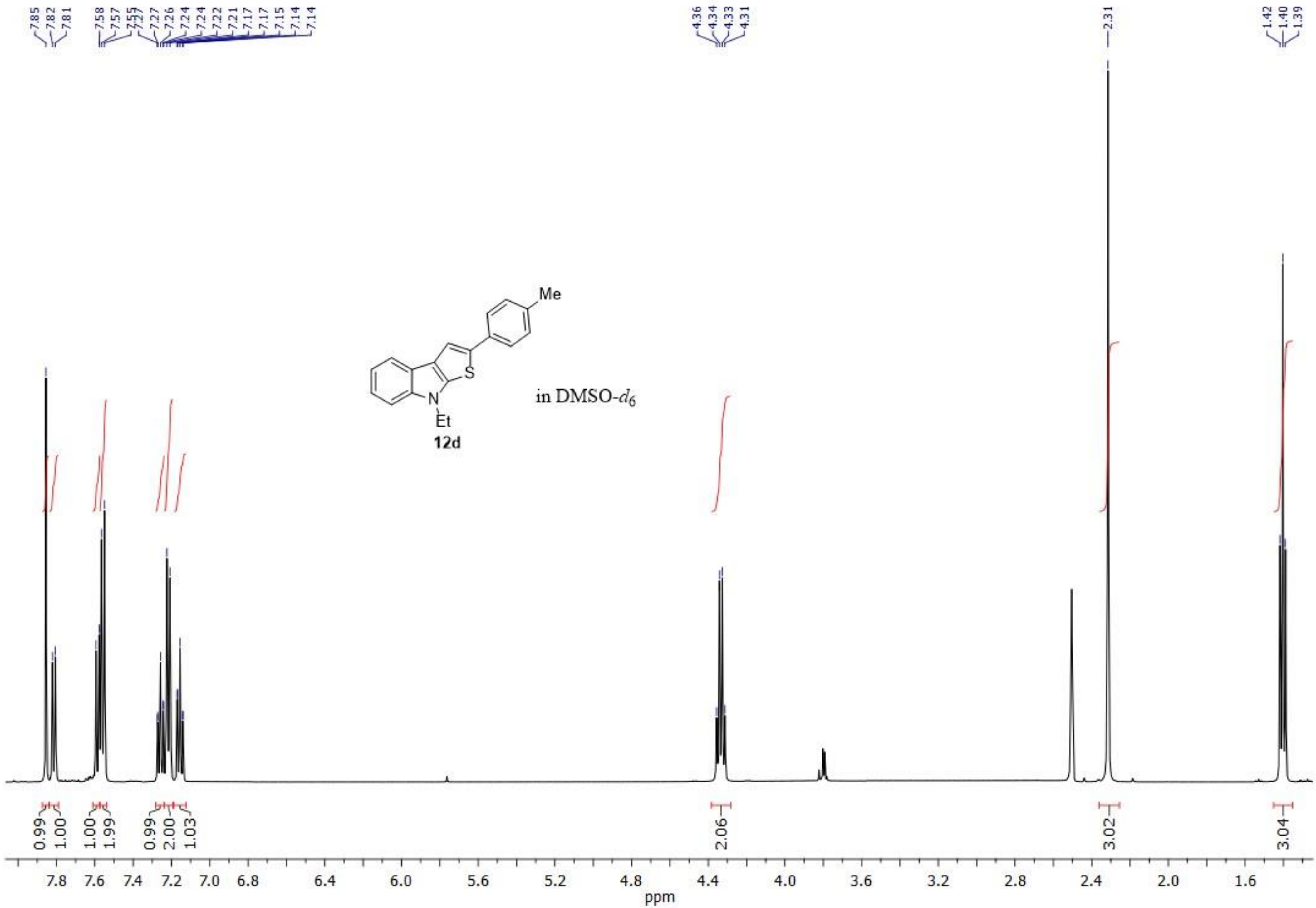
Me
S
N
Et
12d
in DMSO-d_6
ppm

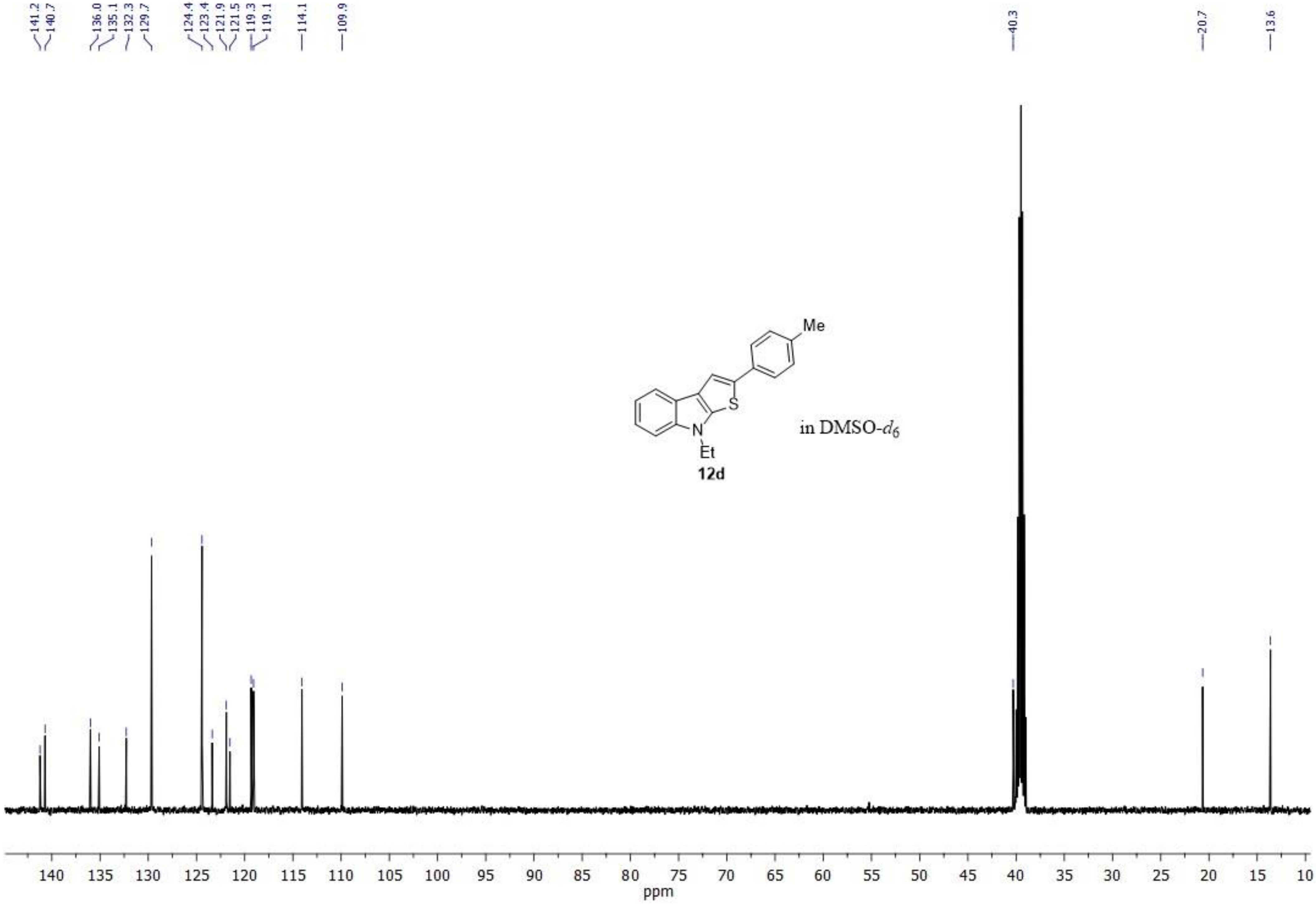
141.2
140.7
136.0
135.1
132.3
129.7
124.4
123.4
121.9
121.5
119.3
119.1
114.1
109.9
40.3
20.7
13.6
Me
S
N
Et
12d
in DMSO-d_6
140 135 130 125 120 115 110 105 100 95 90 85 80 75 70 65 60 55 50 45 40 35 30 25 20 15 10
ppm

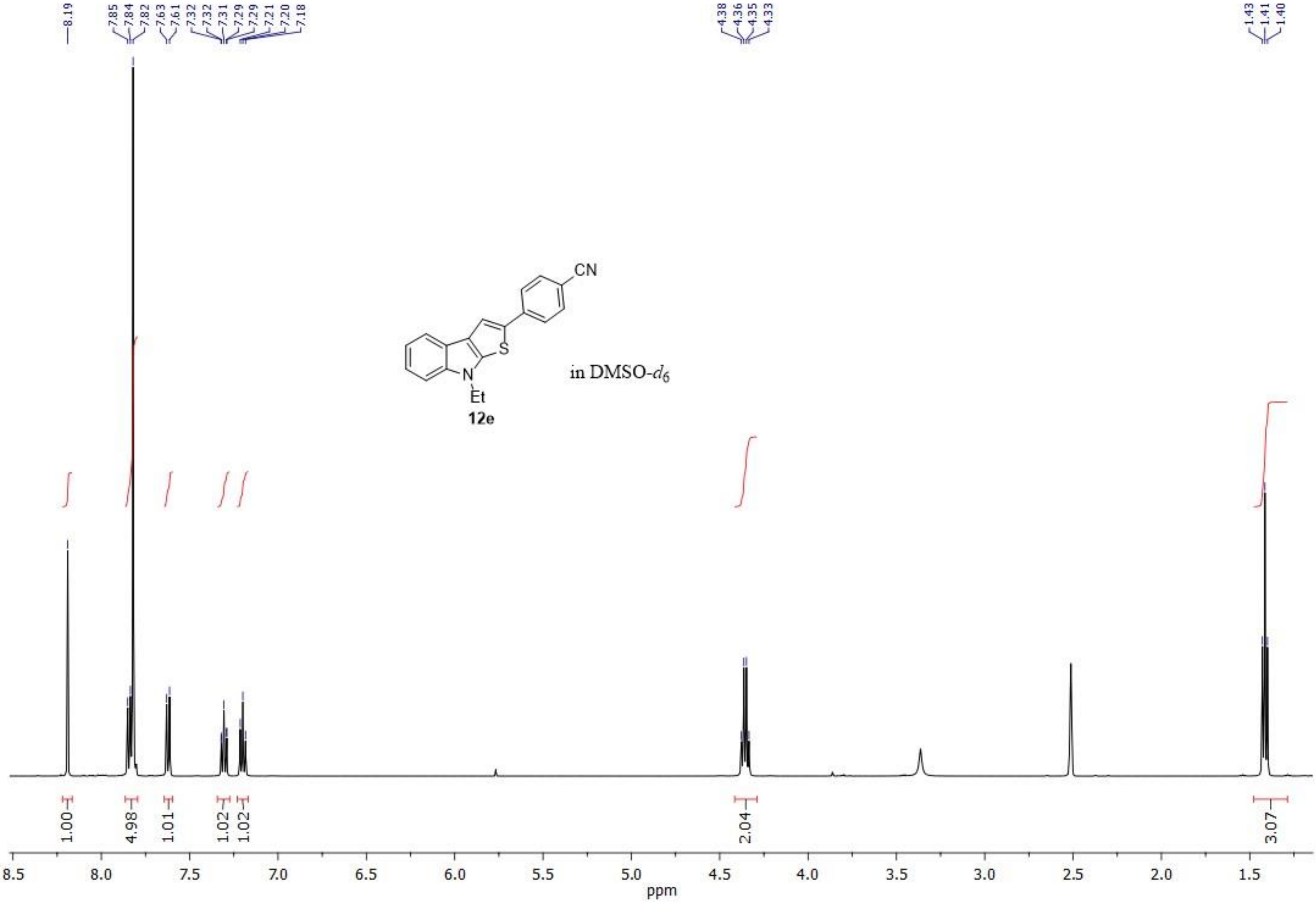
8.19
7.85
7.84
7.82
7.63
7.61
7.32
7.32
7.31
7.29
7.29
7.21
7.20
7.18
4.38
4.36
4.35
4.33
1.43
1.41
1.40
CN
S
N
Et
12e
in DMSO-d_6
1.00
4.98
1.01
1.02
1.02
2.04
3.07
8.5
8.0
7.5
7.0
6.5
6.0
5.5
5.0
4.5
4.0
3.5
3.0
2.5
2.0
1.5
ppm

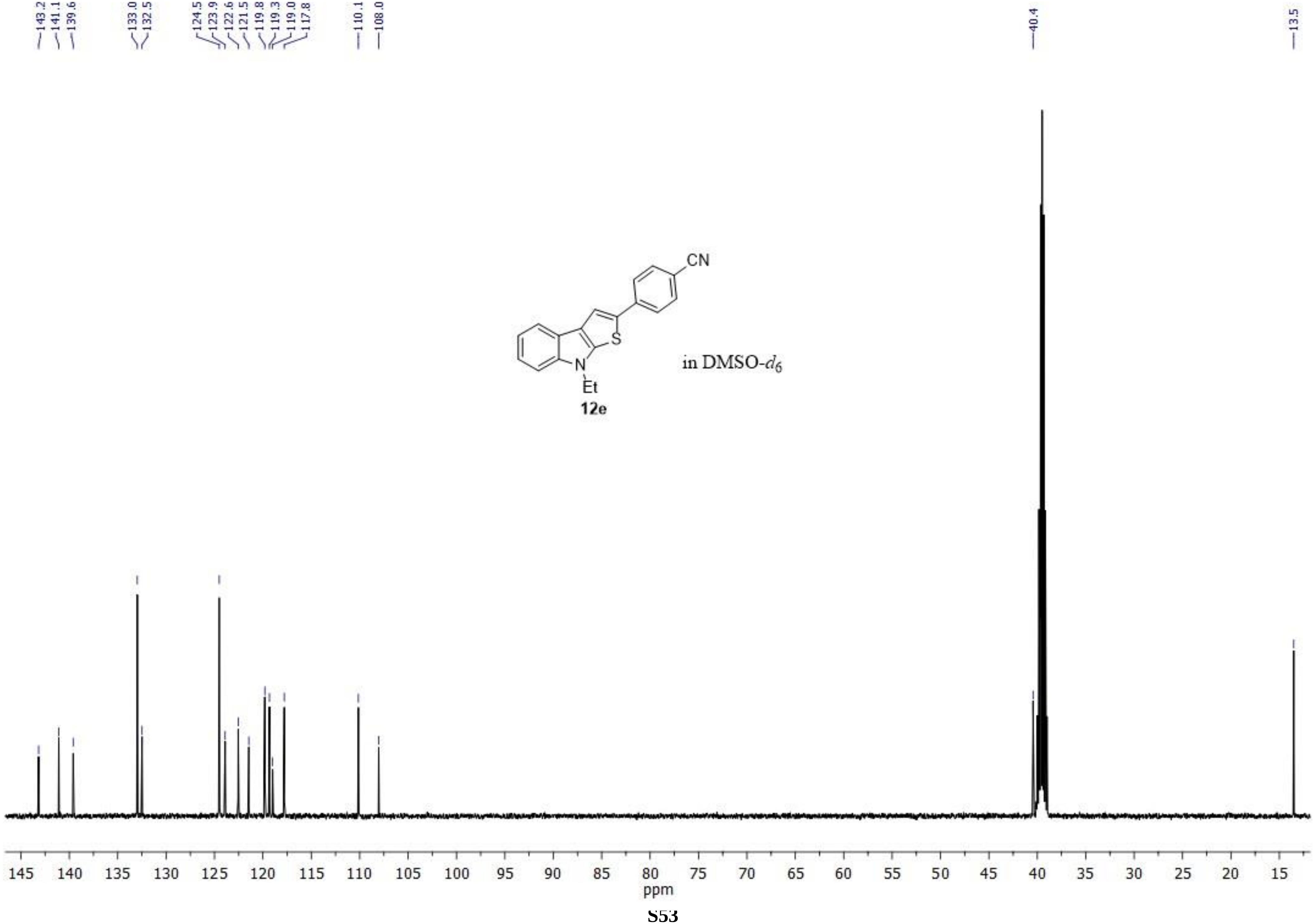
143.2
141.1
139.6
133.0
132.5
124.5
123.9
122.6
121.5
119.8
119.3
119.0
117.8
110.1
108.0
40.4
13.5
CN
S
N
Et
12e
in DMSO-d_6
145 140 135 130 125 120 115 110 105 100 95 90 85 80 75 70 65 60 55 50 45 40 35 30 25 20 15
ppm

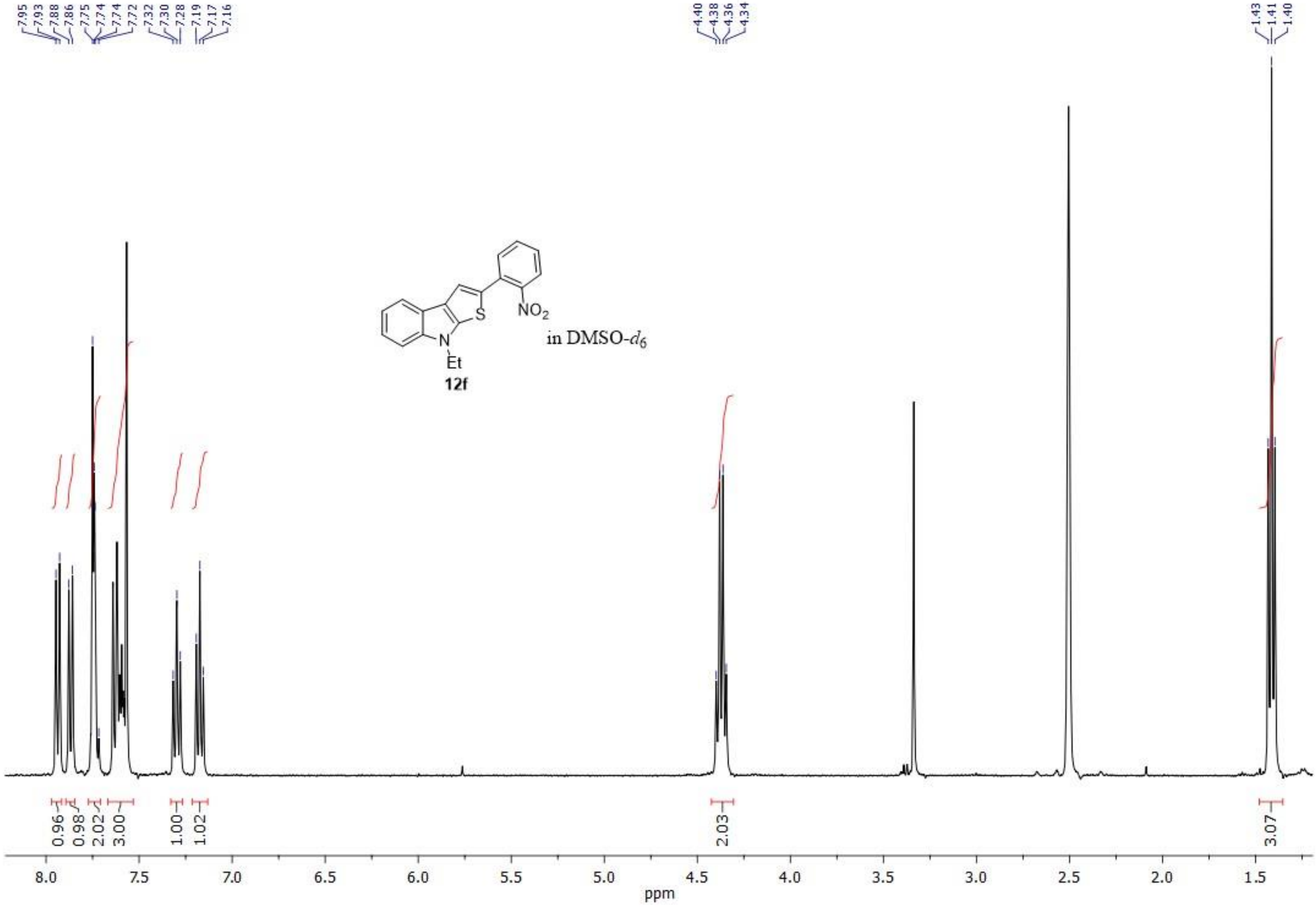
7.95 7.93 7.88 7.86 7.75 7.74 7.74 7.72 7.32 7.30 7.28 7.19 7.17 7.16
4.40 4.38 4.36 4.34
1.43 1.41 1.40
NO2
S
N
Et
12f
in DMSO-d6
0.96
0.98
2.02
3.00
1.00
1.02
2.03
3.07
8.0 7.5 7.0 6.5 6.0 5.5 5.0 4.5 4.0 3.5 3.0 2.5 2.0 1.5
ppm

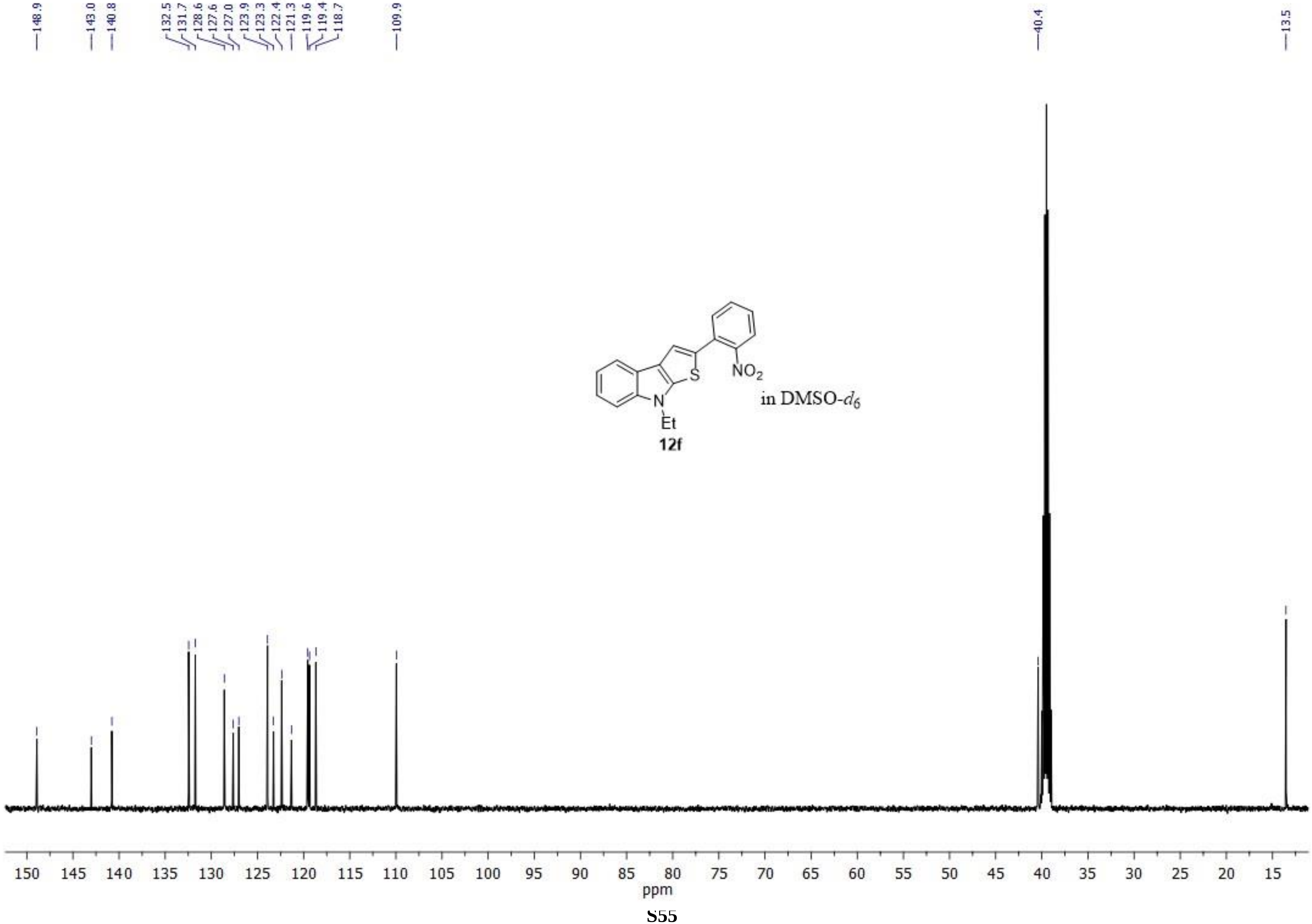
148.9
143.0
140.8
132.5
131.7
128.6
127.6
127.0
123.9
123.3
122.4
121.3
119.6
119.4
118.7
109.9
40.4
13.5
S
NO2
N
Et
12f
in DMSO-d6
150 145 140 135 130 125 120 115 110 105 100 95 90 85 80 75 70 65 60 55 50 45 40 35 30 25 20 15
ppm

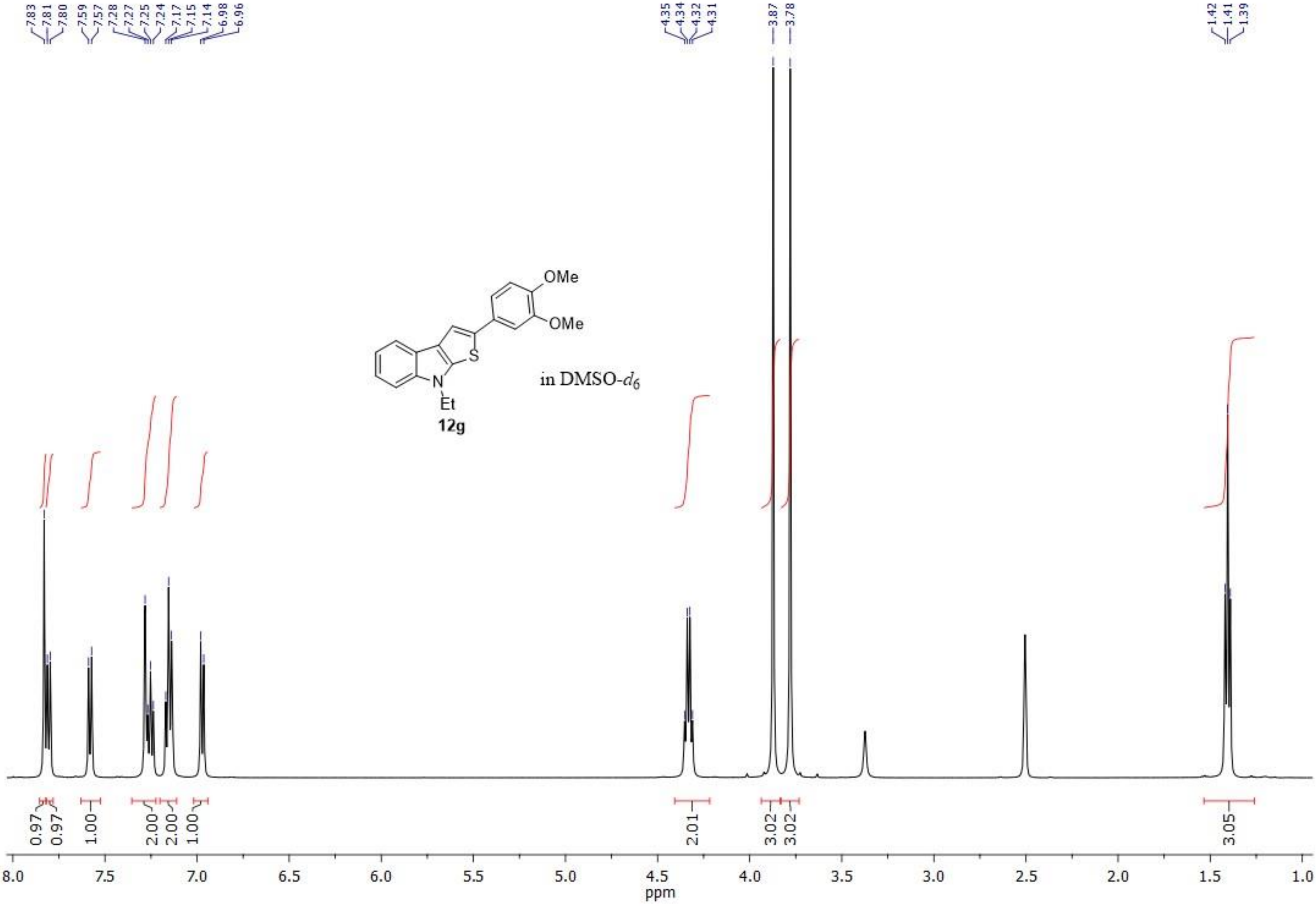
7.83 7.81 7.80 7.59 7.57 7.28 7.27 7.25 7.24 7.17 7.15 7.14 6.98 6.96
4.35 4.34 4.32 4.31
3.87
3.78
1.42 1.41 1.39
OMe
OMe
S
N
Et
12g
in DMSO-d_6
0.97 0.97 1.00 2.00 2.00 1.00
2.01
3.02 3.02
3.05
8.0 7.5 7.0 6.5 6.0 5.5 5.0 4.5 4.0 3.5 3.0 2.5 2.0 1.5 1.0
ppm

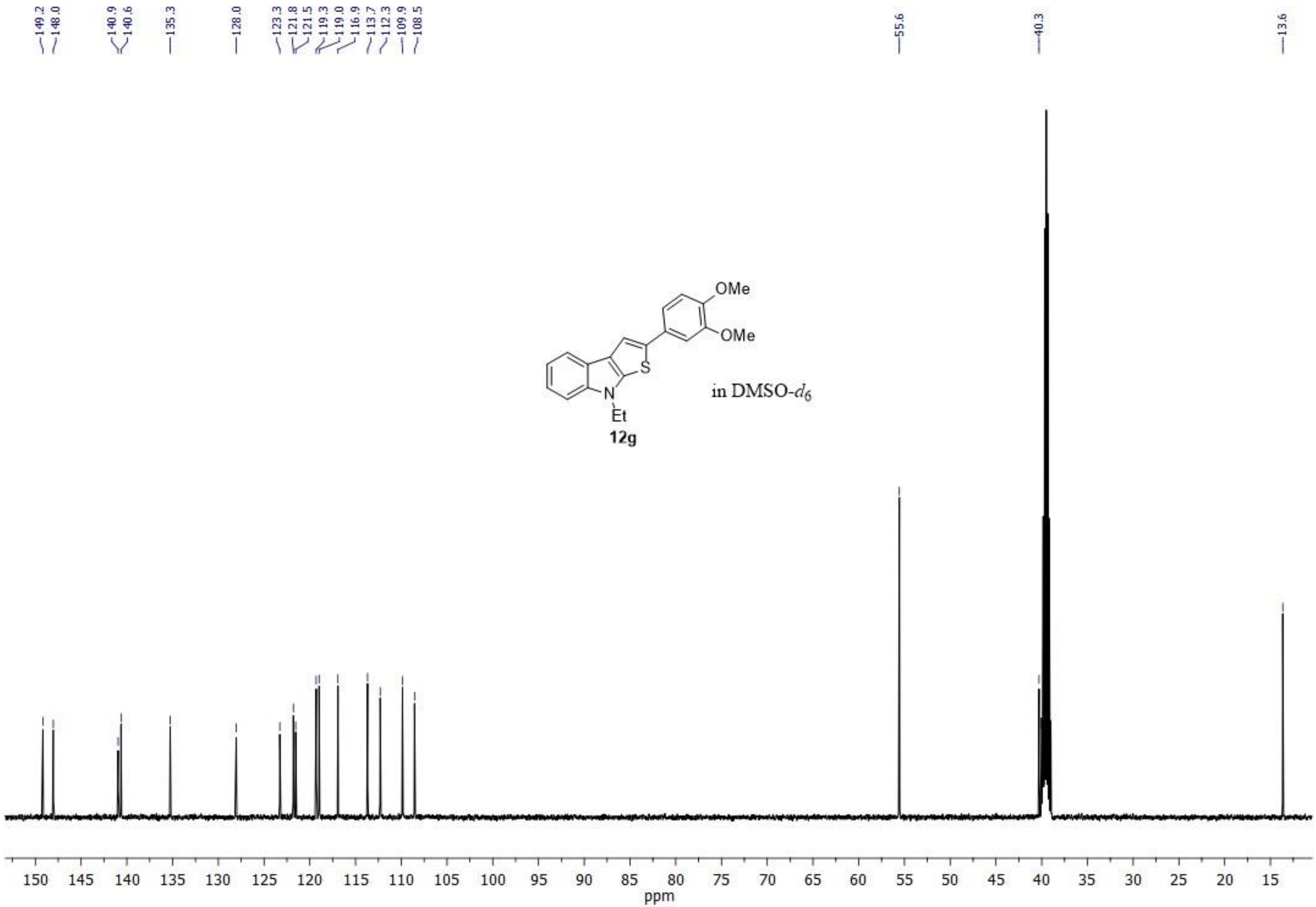
149.2
148.0
140.9
140.6
135.3
128.0
123.3
121.8
121.5
119.3
119.0
116.9
113.7
112.3
109.9
108.5
55.6
40.3
13.6
OMe
OMe
S
N
Et
12g
in DMSO-d_6
150 145 140 135 130 125 120 115 110 105 100 95 90 85 80 75 70 65 60 55 50 45 40 35 30 25 20 15
ppm

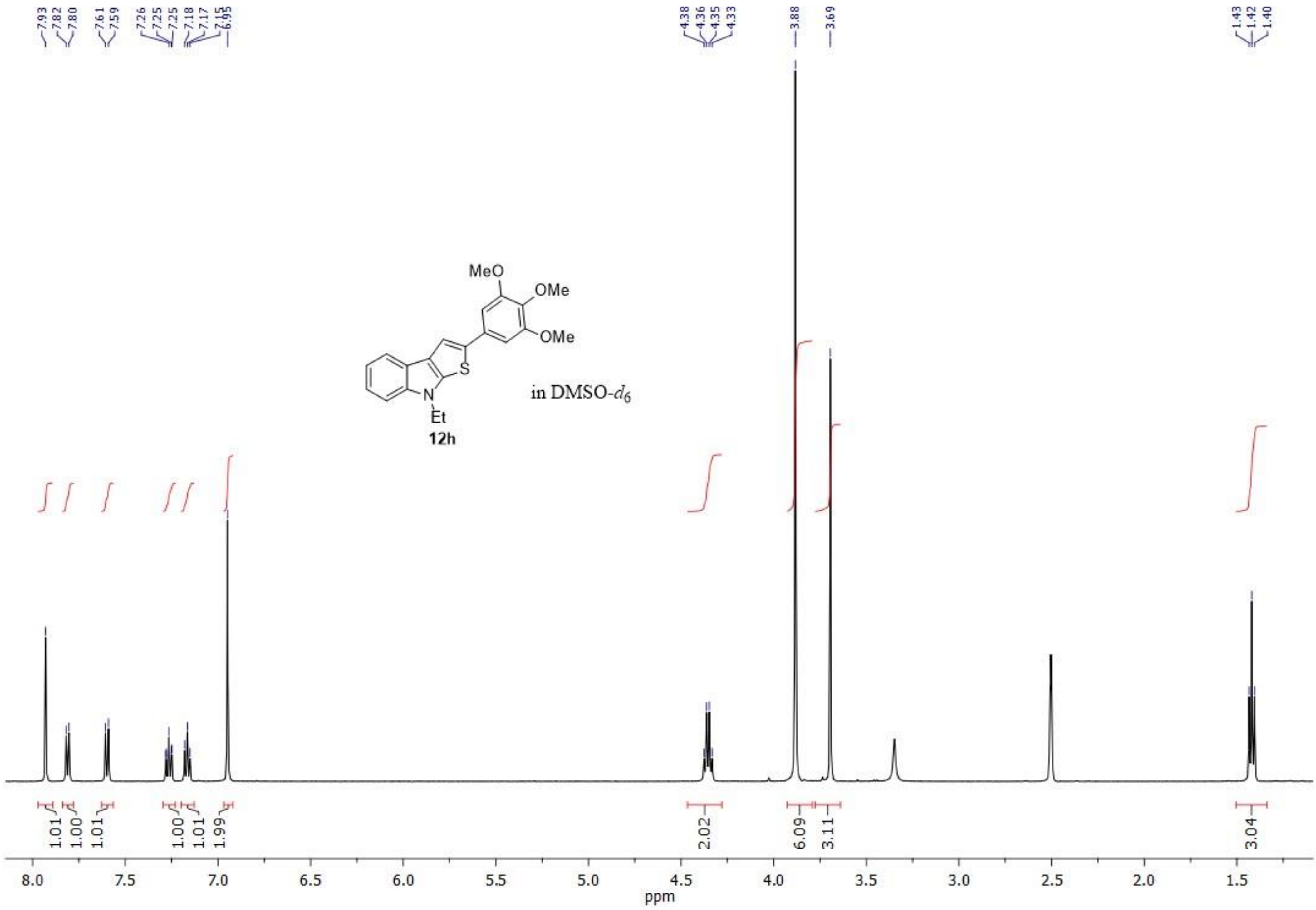
7.93
7.82
7.80
7.61
7.59
7.26
7.25
7.25
7.18
7.17
7.15
6.95
4.38
4.36
4.35
4.33
3.88
3.69
1.43
1.42
1.40
MeO
OMe
OMe
S
N
Et
12h
in DMSO-d6
1.01
1.00
1.01
1.00
1.01
1.99
2.02
6.09
3.11
3.04
8.0
7.5
7.0
6.5
6.0
5.5
5.0
4.5
4.0
3.5
3.0
2.5
2.0
1.5
ppm

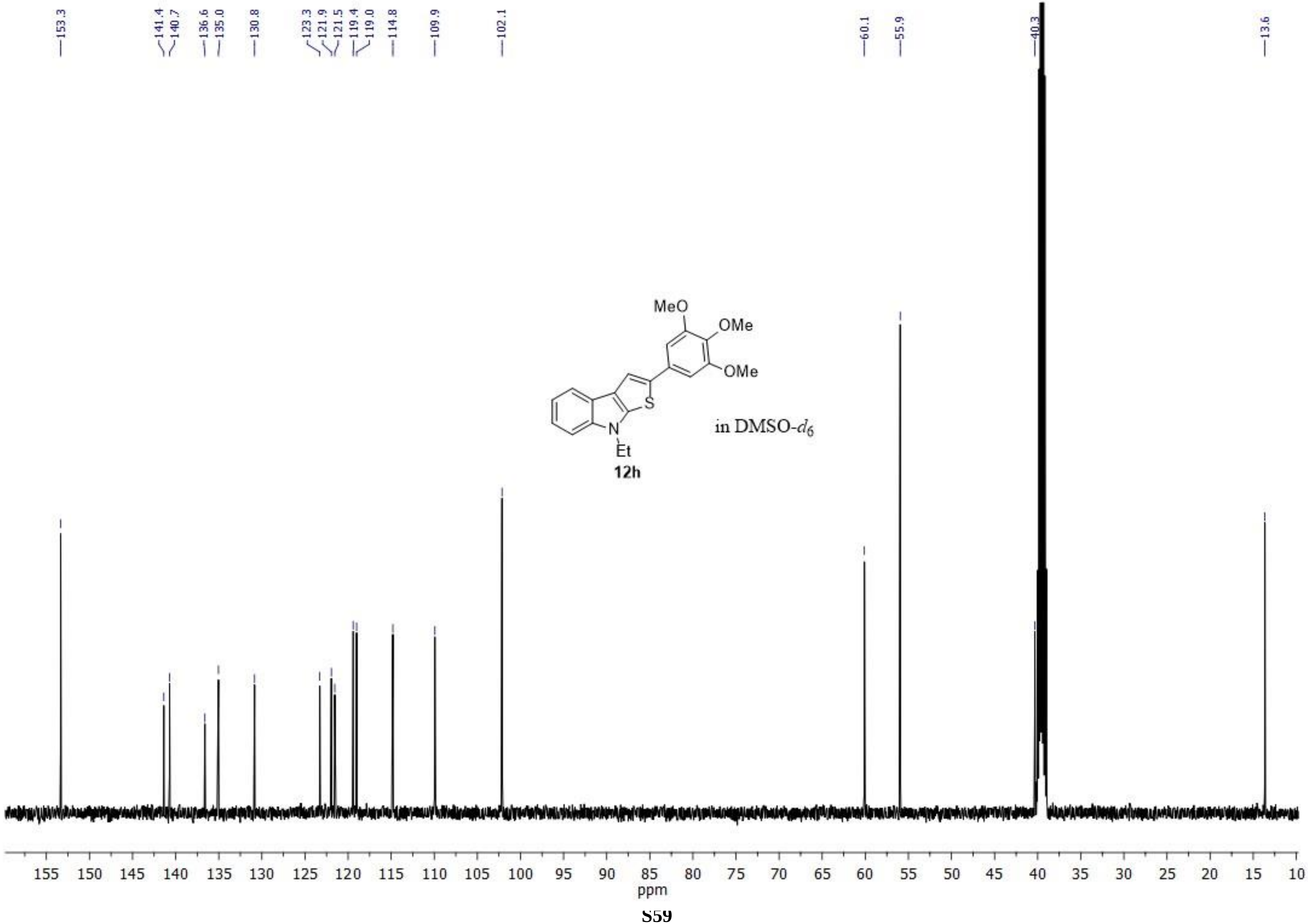
153.3
141.4
140.7
136.6
135.0
130.8
123.3
121.9
121.5
119.4
119.0
114.8
109.9
102.1
60.1
55.9
40.3
13.6
MeO
OMe
OMe
S
N
Et
12h
in DMSO-d_6
155 150 145 140 135 130 125 120 115 110 105 100 95 90 85 80 75 70 65 60 55 50 45 40 35 30 25 20 15 10
ppm

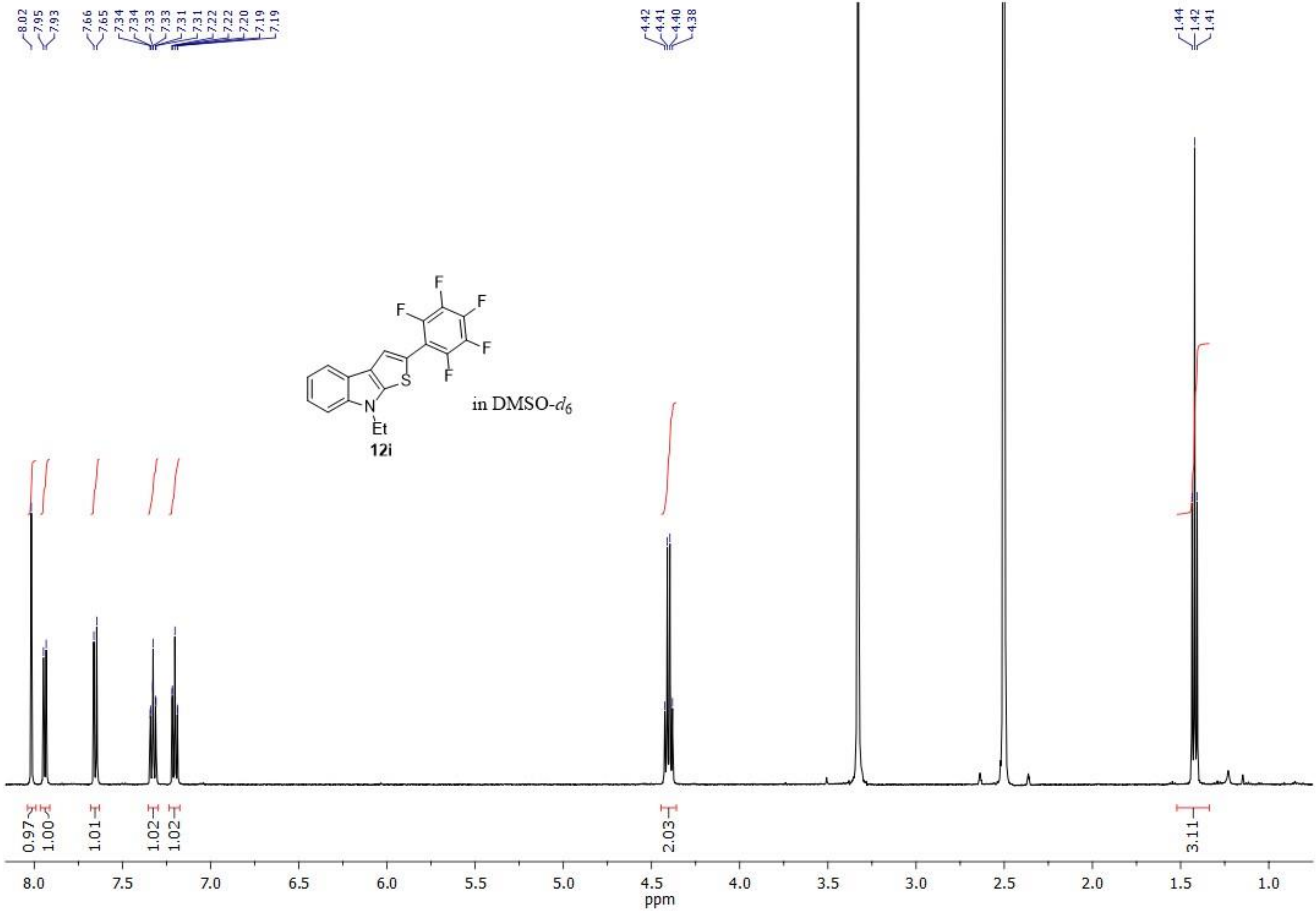
8.02
7.95
7.93
7.66
7.65
7.34
7.34
7.33
7.33
7.31
7.31
7.22
7.22
7.20
7.19
7.19
4.42
4.41
4.40
4.38
1.44
1.42
1.41
F
F
F
F
F
S
N
Et
12i
in DMSO-d6
0.97
1.00
1.01
1.02
1.02
2.03
3.11
8.0
7.5
7.0
6.5
6.0
5.5
5.0
4.5
4.0
3.5
3.0
2.5
2.0
1.5
1.0
ppm

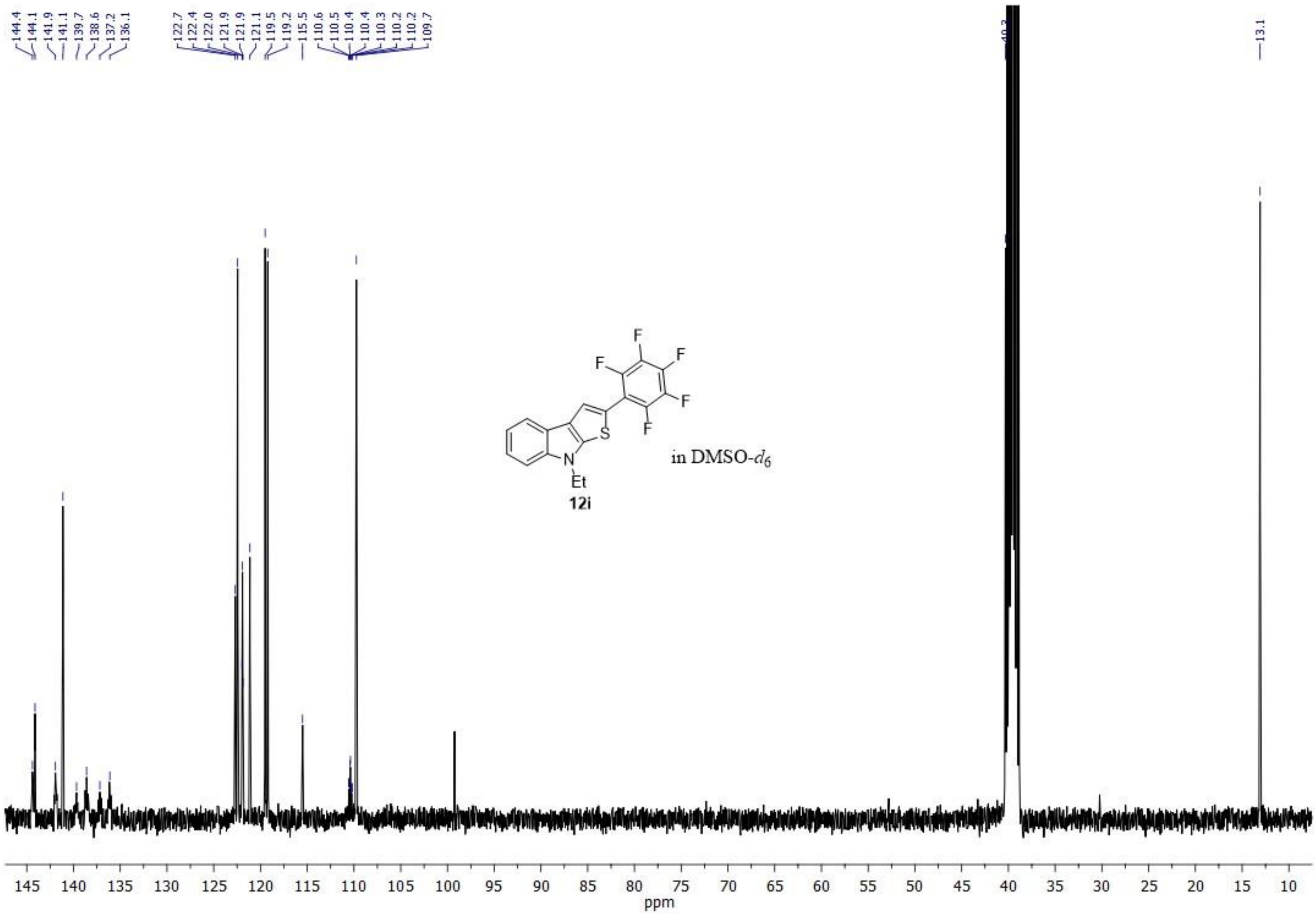
144.4
144.1
141.9
141.1
139.7
138.6
137.2
136.1
122.7
122.4
122.0
121.9
121.9
121.1
119.5
119.2
115.5
110.6
110.5
110.4
110.4
110.3
110.2
110.2
109.7
13.1
F
F
F
F
F
S
N
Et
12i
in DMSO-d_6
145 140 135 130 125 120 115 110 105 100 95 90 85 80 75 70 65 60 55 50 45 40 35 30 25 20 15 10
ppm

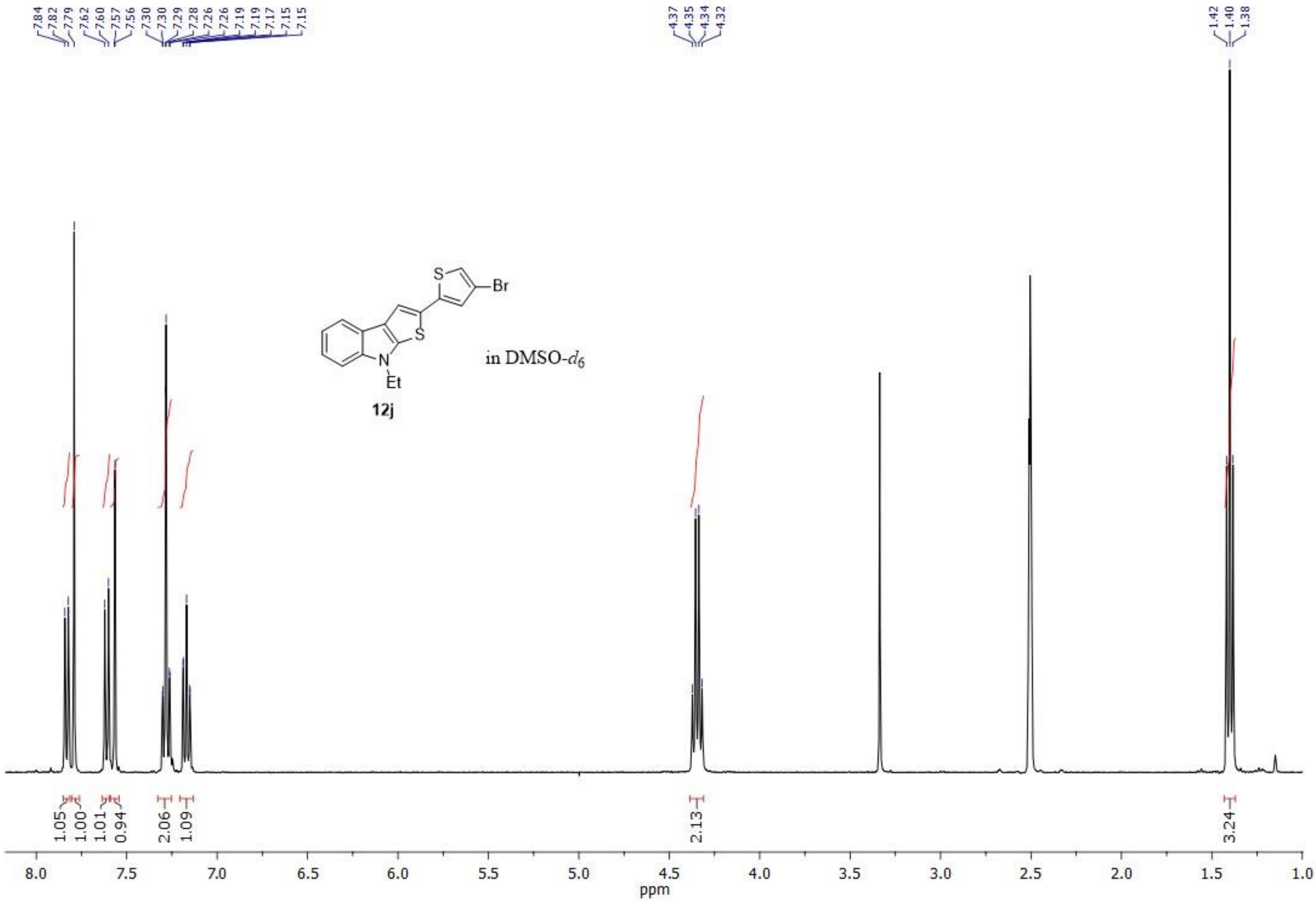
7.84
7.82
7.79
7.62
7.60
7.57
7.56
7.30
7.30
7.29
7.28
7.26
7.26
7.19
7.19
7.17
7.15
7.15
4.37
4.35
4.34
4.32
1.42
1.40
1.38
S
Br
S
N
Et
12j
in DMSO-d_6
1.05
1.00
1.01
0.94
2.06
1.09
2.13
3.24
8.0
7.5
7.0
6.5
6.0
5.5
5.0
4.5
4.0
3.5
3.0
2.5
2.0
1.5
1.0
ppm

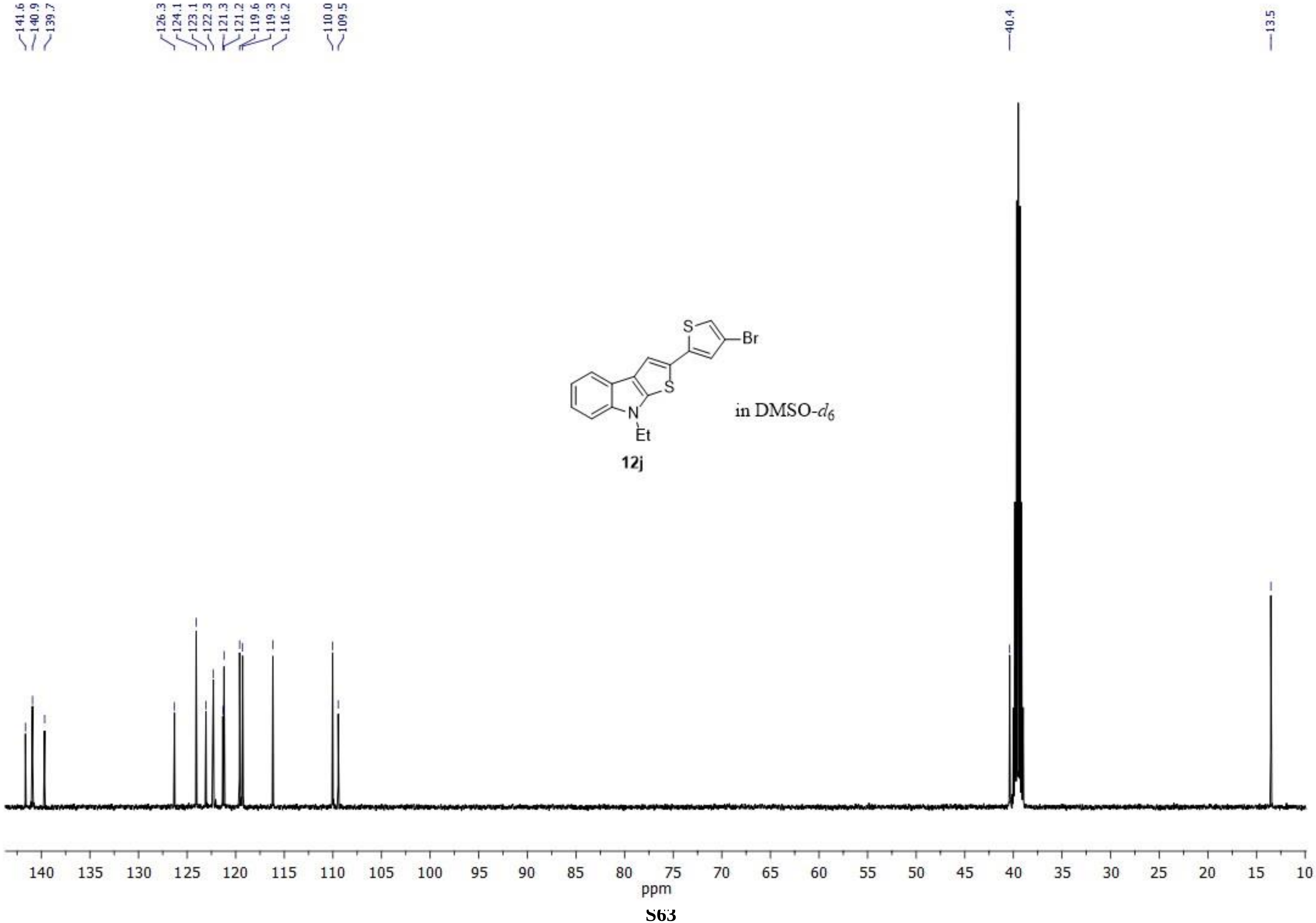
141.6
140.9
139.7
126.3
124.1
123.1
122.3
121.3
121.2
119.6
119.3
116.2
110.0
109.5
40.4
13.5
S
Br
S
N
Et
12j
in DMSO-d_6
140 135 130 125 120 115 110 105 100 95 90 85 80 75 70 65 60 55 50 45 40 35 30 25 20 15 10
ppm

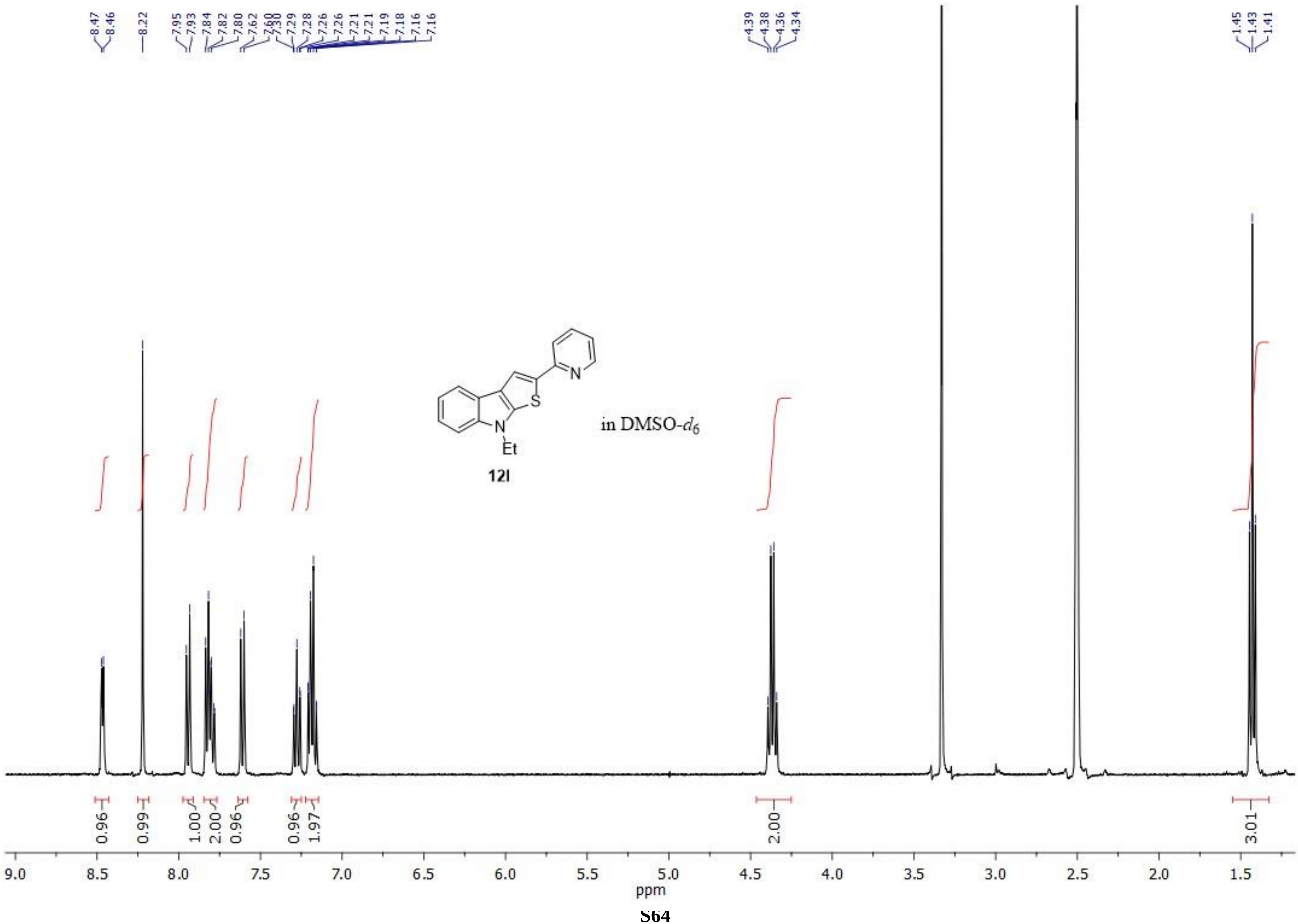
8.47
8.46
8.22
7.95
7.93
7.84
7.82
7.80
7.62
7.60
7.30
7.29
7.28
7.26
7.26
7.21
7.21
7.19
7.18
7.16
7.16
4.39
4.38
4.36
4.34
1.45
1.43
1.41
N
S
N
Et
12l
in DMSO-d_6
0.96
0.99
1.00
2.00
0.96
0.96
1.97
2.00
3.01
9.0
8.5
8.0
7.5
7.0
6.5
6.0
5.5
5.0
4.5
4.0
3.5
3.0
2.5
2.0
1.5
ppm

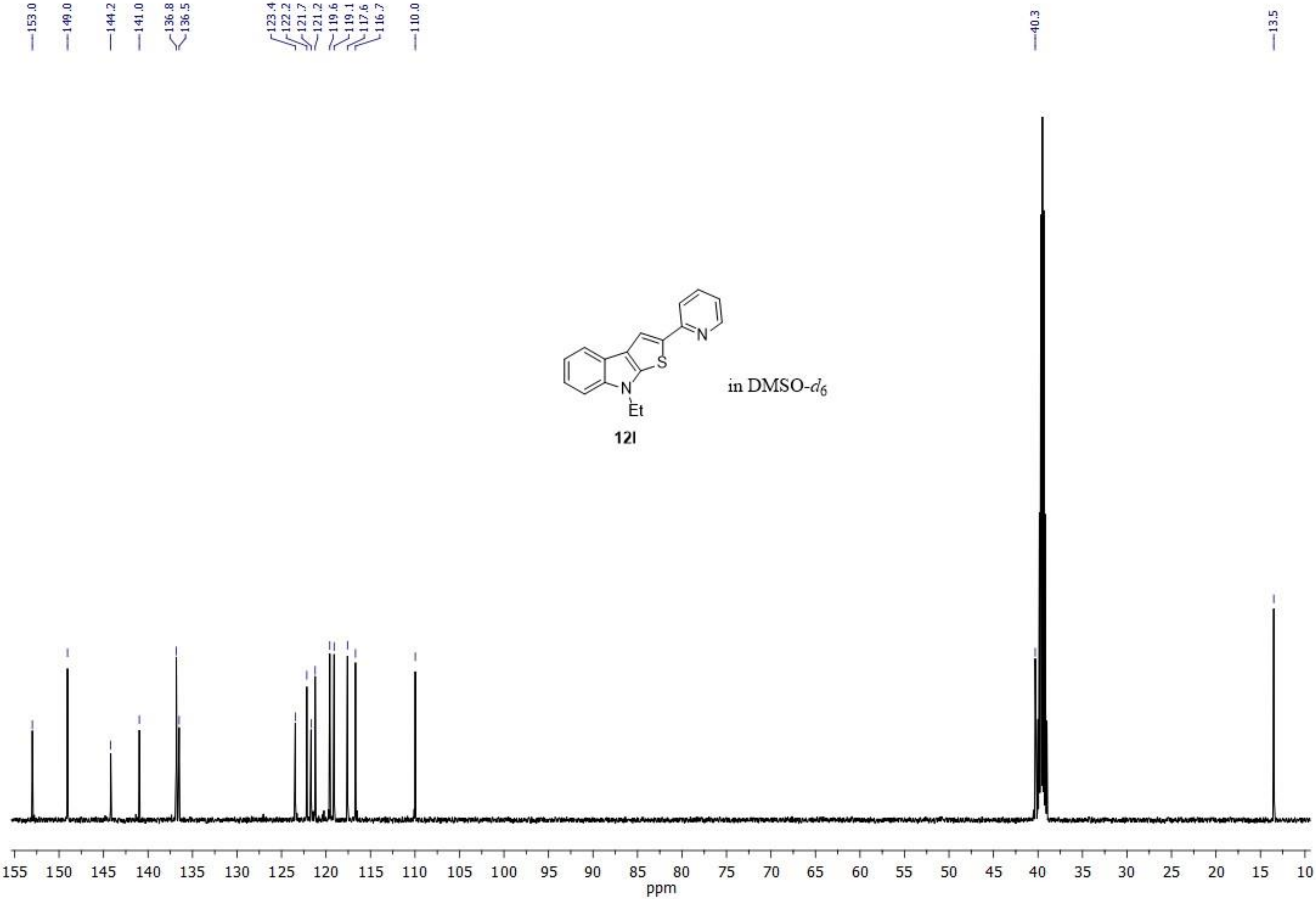
153.0
149.0
144.2
141.0
136.8
136.5
123.4
122.2
121.7
121.2
119.6
119.1
117.6
116.7
110.0
40.3
13.5
S
N
N
Et
12l
in DMSO-d6
155 150 145 140 135 130 125 120 115 110 105 100 95 90 85 80 75 70 65 60 55 50 45 40 35 30 25 20 15 10
ppm

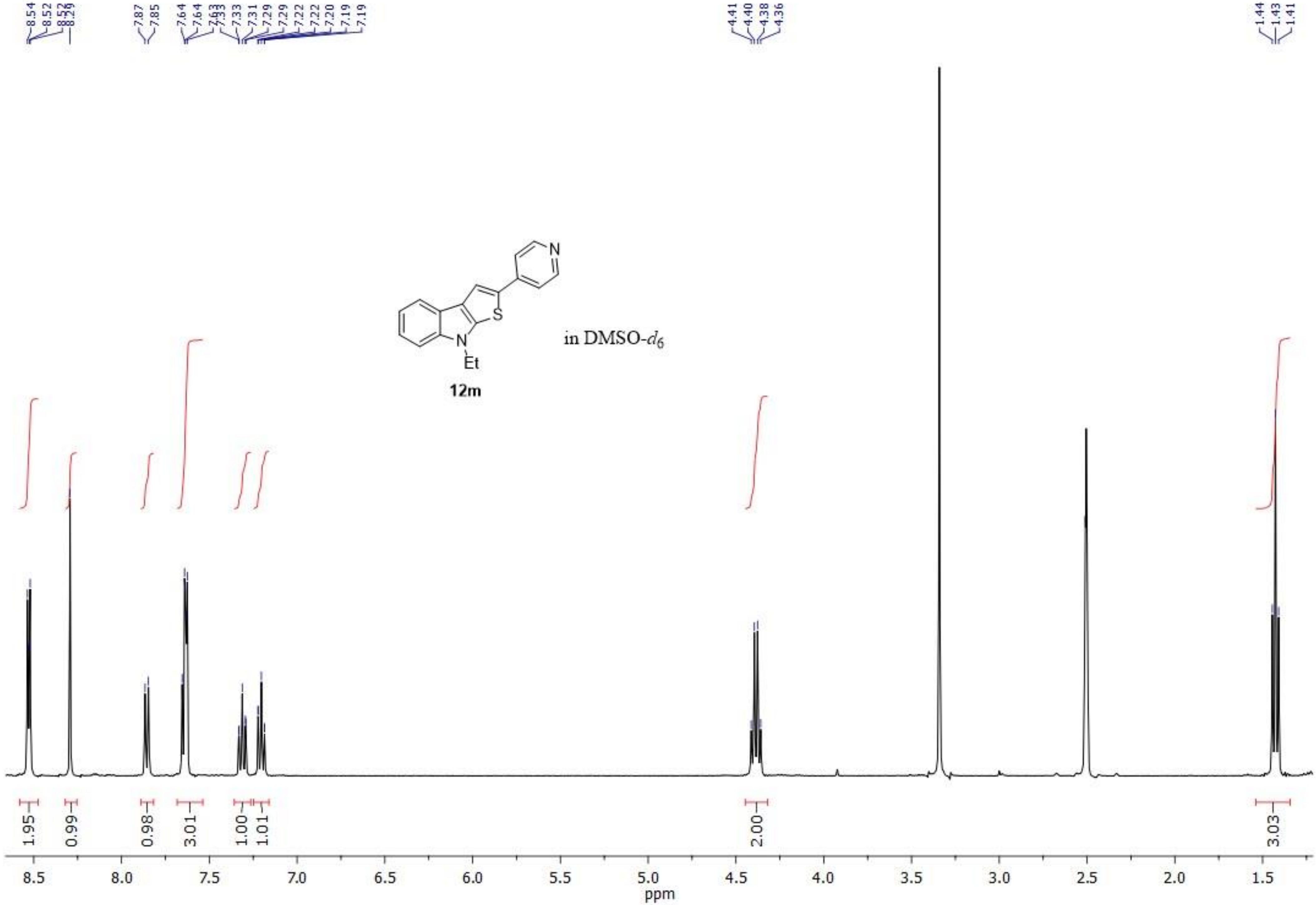
8.54
8.52
8.52
8.29
7.87
7.85
7.64
7.64
7.63
7.33
7.33
7.31
7.29
7.29
7.22
7.22
7.20
7.19
7.19
4.41
4.40
4.38
4.36
1.44
1.43
1.41
N
S
N
Et
12m
in DMSO-d_6
1.95
0.99
0.98
3.01
1.00
1.01
2.00
3.03
8.5
8.0
7.5
7.0
6.5
6.0
5.5
5.0
4.5
4.0
3.5
3.0
2.5
2.0
1.5
ppm

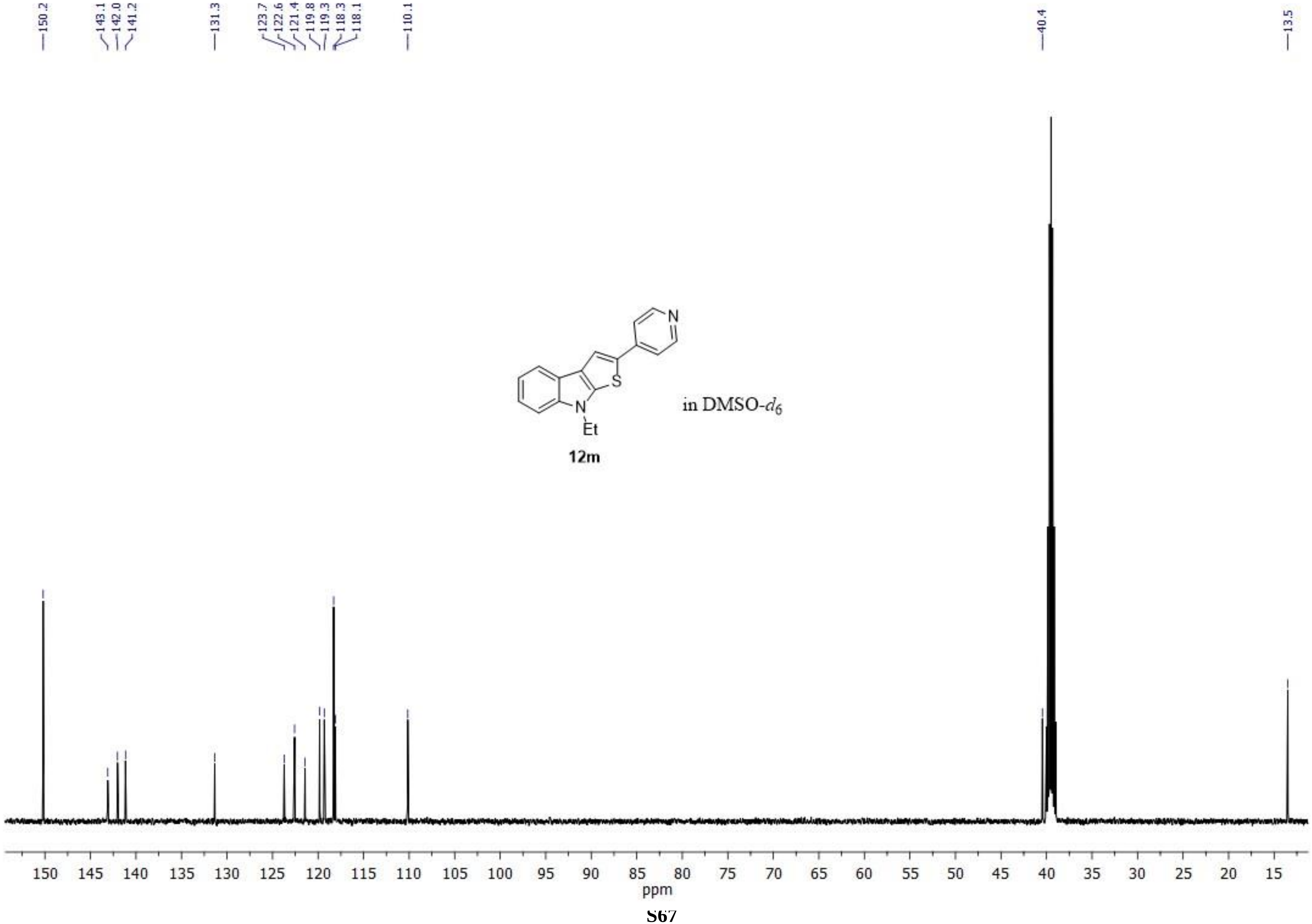
150.2
143.1
142.0
141.2
131.3
123.7
122.6
121.4
119.8
119.3
118.3
118.1
110.1
40.4
13.5
N
S
N
Et
12m
in DMSO-d_6
150 145 140 135 130 125 120 115 110 105 100 95 90 85 80 75 70 65 60 55 50 45 40 35 30 25 20 15
ppm

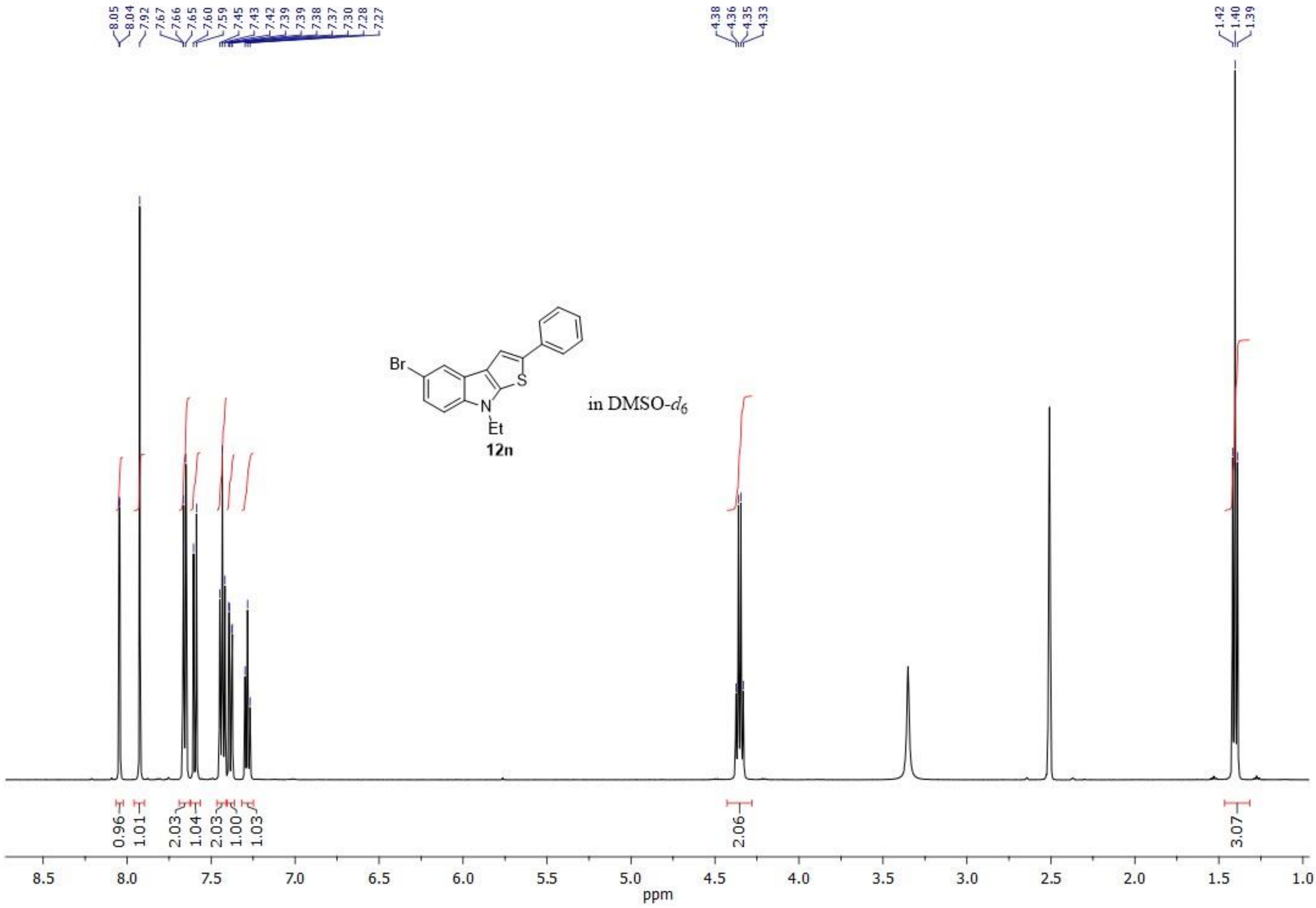
8.05
8.04
7.92
7.67
7.66
7.65
7.60
7.59
7.45
7.43
7.42
7.39
7.39
7.38
7.37
7.30
7.28
7.27
4.38
4.36
4.35
4.33
1.42
1.40
1.39
Br
S
N
Et
12n
in DMSO-d6
0.96
1.01
2.03
1.04
2.03
1.00
1.03
2.06
3.07
8.5
8.0
7.5
7.0
6.5
6.0
5.5
5.0
4.5
4.0
3.5
3.0
2.5
2.0
1.5
1.0
ppm

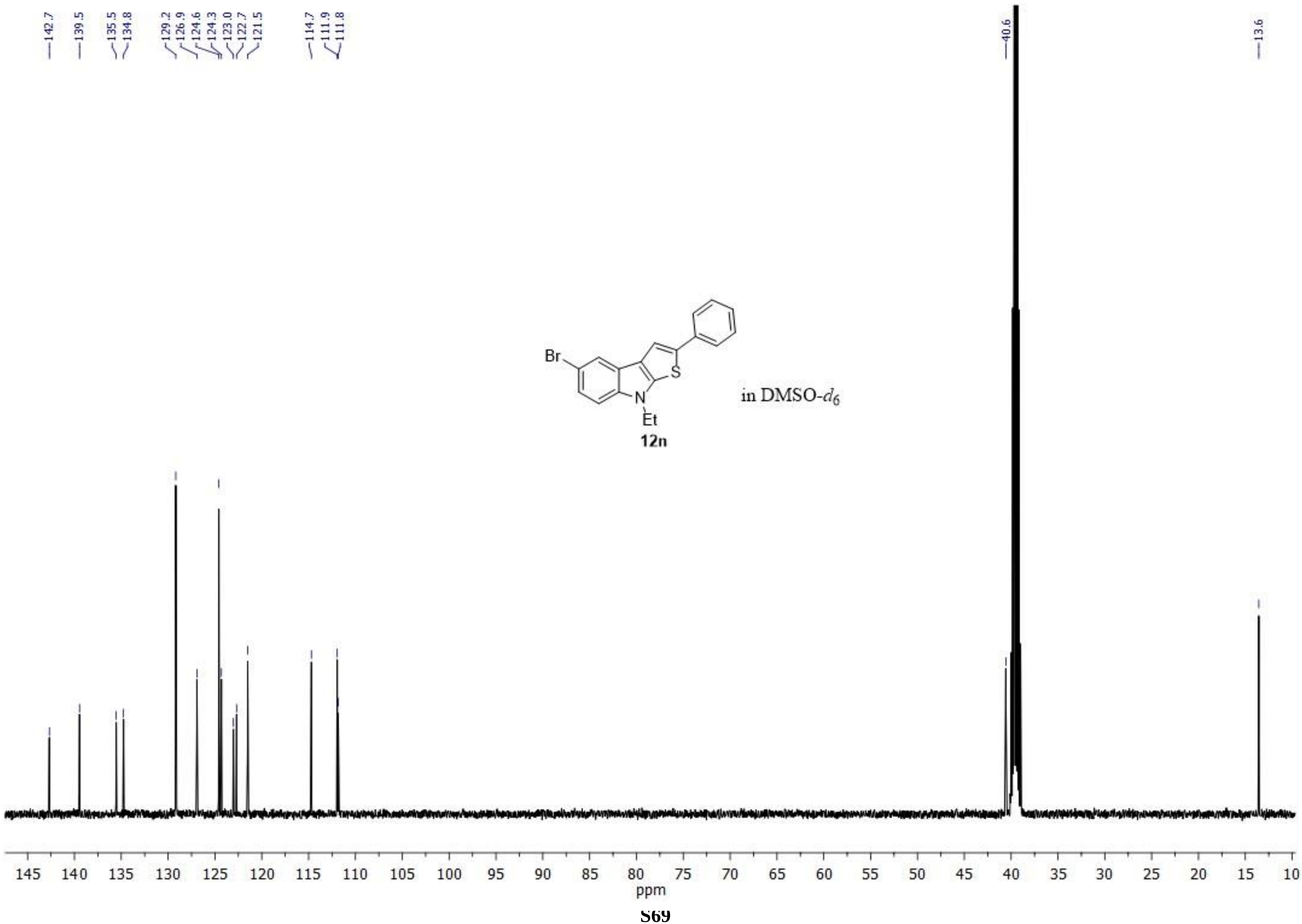
142.7
139.5
135.5
134.8
129.2
126.9
124.6
124.3
123.0
122.7
121.5
114.7
111.9
111.8
40.6
13.6
Br
S
N
Et
12n
in DMSO-d_6
145 140 135 130 125 120 115 110 105 100 95 90 85 80 75 70 65 60 55 50 45 40 35 30 25 20 15 10
ppm

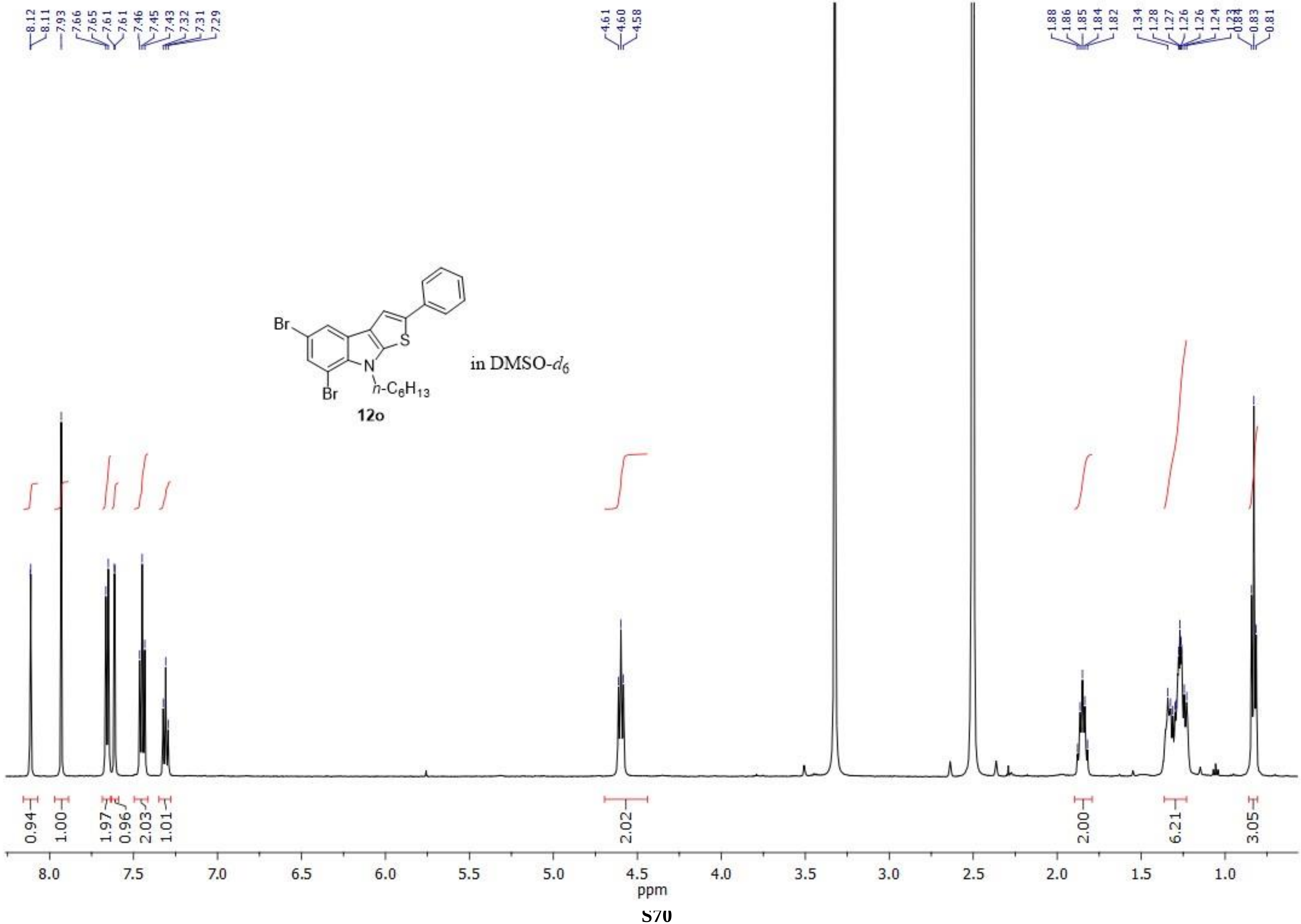

8.12
8.11
7.93
7.66
7.65
7.61
7.61
7.46
7.45
7.43
7.32
7.31
7.29
4.61
4.60
4.58
1.88
1.86
1.85
1.84
1.82
1.34
1.28
1.27
1.26
1.26
1.24
1.23
0.84
0.83
0.81
Br
Br
S
N
n-C6H13
12o
in DMSO-d6
0.94
1.00
1.97
0.96
2.03
1.01
2.02
2.00
6.21
3.05
8.0
7.5
7.0
6.5
6.0
5.5
5.0
4.5
4.0
3.5
3.0
2.5
2.0
1.5
1.0
ppm

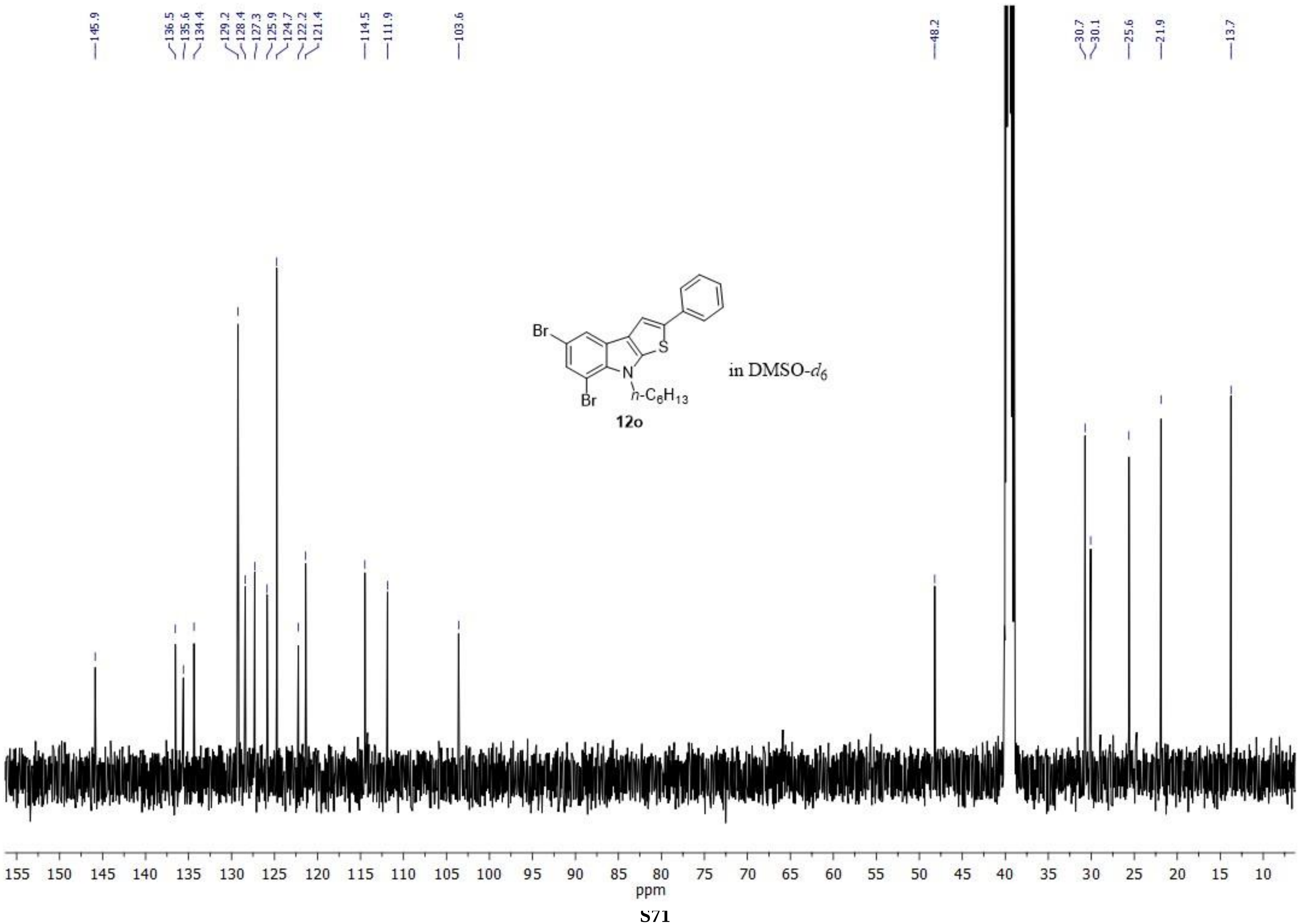
145.9
136.5
135.6
134.4
129.2
128.4
127.3
125.9
124.7
122.2
121.4
114.5
111.9
103.6
48.2
30.7
30.1
25.6
21.9
13.7
Br
Br
S
N
n-C6H13
12o
in DMSO-d6
155 150 145 140 135 130 125 120 115 110 105 100 95 90 85 80 75 70 65 60 55 50 45 40 35 30 25 20 15 10
ppm
